# Plasmonic Coupling Effects in Metal Clusters Supported over TiO_2_: A Theoretical Study

**DOI:** 10.1021/acs.jpcc.5c08085

**Published:** 2026-05-21

**Authors:** Parfaite Senoume Senou, Monica Calatayud, Ardhmeri Alija, Pierpaolo D’Antoni, Daniele Toffoli, Rilinda Plakaj, Mauro Stener

**Affiliations:** † 27063 Sorbonne Université, MONARIS, CNRS-UMR 8233, 4 Place Jussieu, Paris F-75005, France; ‡ Dipartimento di Scienze Chimiche e Farmaceutiche, 9315 Università di Trieste, Via Giorgieri 1, Trieste 34127, Italy

## Abstract

Density functional theory (DFT) and time-dependent density functional theory (TDDFT) have been employed to study the plasmonic coupling effects of silver, gold, and copper clusters supported on titania. The adsorption of metal nanoparticles on the surface of rutile TiO_2_ changes the electronic properties of the system, resulting in the disappearance of the TiO_2_ bandgap. The analysis of photoabsorption spectra allowed us to identify surface plasmons and quantify their contribution to the absorption of visible light. Using specific analysis tools, we evaluated the strength of the plasmonic coupling between metal nanoparticles and TiO_2_, allowing the identification of favorable configurations for the plasmonic coupling.

## Graphical abstract

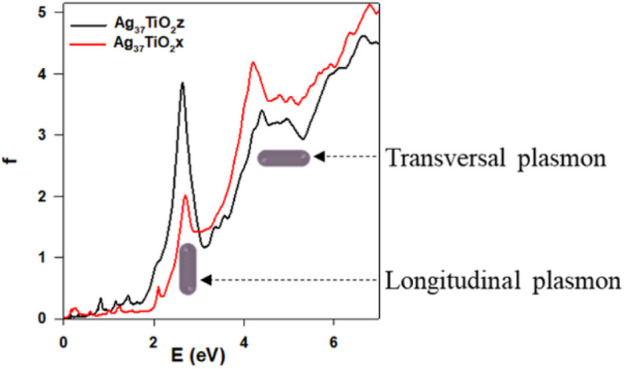

## 1 Introduction

Photocatalysis based on semiconductor materials represents a key strategy for environmental pollutant degradation and sustainable energy production.^1–4^ Among the various candidates, titanium dioxide (TiO_2_) has emerged as a benchmark material thanks to its chemical stability, low cost, and nontoxicity.^5–9^ However, its wide band gap and the quick recombination of photogenerated charge carriers significantly limit its activity under visible light irradiation. Consequently, considerable efforts have been devoted to the development of highly efficient TiO_2_-based photocatalysts through various modification strategies.^10–16^ One of the most widely explored approaches consists of coupling TiO_2_ with plasmonic metal nanoparticles such as Au, Ag, or Cu.^17–26^

More recently, single atoms combined with alloy nanoparticles supported on TiO_2_ have demonstrated strong synergistic effects and remarkable improvements in photocatalytic CO_2_ reduction efficiency.^27^ In such hybrid systems, metal nanostructures do not merely act as passive cocatalysts; rather, they introduce collective electronic excitations,^28^ namely localized surface plasmons,^29–31^ which can substantially modify the optical response of the composite material.^32^ The interaction between metal plasmons and the semiconductor support may result in enhanced light absorption, hot carrier generation, and interfacial charge-transfer processes.^33^ These phenomena are now recognized as key mechanisms in plasmon-driven photocatalysis.^34^

Although numerous experimental and theoretical studies have investigated metal/TiO_2_ heterostructures, most theoretical works have predominantly focused on ground-state properties or simplified descriptions of charge-transfer processes.^35–37^ A comprehensive quantum-mechanical characterization of plasmonic coupling at the cluster–semiconductor interface, particularly including an explicit analysis of the excited-state electronic structure, remains limited. This is especially true for small supported clusters, where quantum size effects may substantially alter the nature of collective excitations.

Recent investigations have shown that cluster size,^23,38^ morphology,^39,40^ and interfacial geometry^41,42^ critically influence plasmon resonance energies and the degree of hybridization between metal and semiconductor states.^43^ Beyond these structural parameters, the chemical nature of the support, its reducibility, the presence of oxygen vacancies, and surface hydroxylation have also been demonstrated to strongly affect nucleation pathways, stability, and electronic properties of supported nanoclusters.^43,44^ These findings highlight the central role of interfacial chemistry in determining the properties of metal–TiO_2_ hybrid systems.

Therefore, further research is needed to deepen our understanding of these phenomena and explore potential applications in areas such as photocatalysis, sensing, and plasmonics.

The main goal of this work is to understand and rationalize the photoabsorption and, in particular, the plasmonic behavior of small nanoparticles supported on TiO_2_, by means of theoretical methods, such as DFT (Density Functional Theory) and TDDFT (Time-Dependent Density Functional Theory). This approach has allowed us to characterize the electronic and optical properties of TiO_2_/metallic nanoparticle systems with great accuracy and with deep detail of the physics that govern this process. This has been possible thanks to the availability of specific analysis tools that have proven to be efficient and informative.

It is worth noting that in this work, it has been possible to employ a very large cluster model for the TiO_2_ surface supporting various metal clusters, thanks to the availability of a very efficient yet accurate TDDFT scheme named polTDDFT.^45^ In order to perform the polTDDFT calculations, it is necessary to have at our disposal a realistic geometrical model of the systems under study. For this reason, we also performed preliminary DFT calculations in order to relax the geometry of metal clusters over the TiO_2_ surface. In order to check the validity of the DFT calculations, we analyzed the results and verified that they guarantee a realistic description of the ground-state properties before calculating the excited states.

The work is organized as follows: in the preliminary stage, (i) we built a structural model accounting for the TiO_2_ support and metal clusters, and then (ii) we optimized the structure at the DFT level. Then, the actual TDDFT calculations of the spectra are performed (iii) using the optimized structure to compute at the TDDFT level the photoabsorption spectra, and (iv) analyzing the spectra in terms of the excited-state electronic structure (composition and fragment analysis).

## 2 Theoretical Method and Computational Details

### 2.1 VASP

In this study, the geometry optimization is performed using the PAW (projector augmented wave) method implemented in the Vienna Ab initio Simulation Package (VASP 6.3.2).^46^ The electron exchange and correlation terms are treated with the Perdew–Burke–Ernzerhof (PBE) functional.^47^ To describe the TiO_2_ properties, the GGA+*U* approach^48^ is used, applying *U* = 6.0 eV and *J* = 0.5 eV^49^ values to treat the 3d electrons of Ti atoms with a plane wave cutoff energy of 600 eV. Core electrons were replaced by PAW projector-augmented pseudopotentials with the following valence electrons: O(6), Ti(4), Ag(11), Au(11), and Cu(11). The electronic and ionic loops were converged within thresholds of 10^–4^ and 10^–3^ eV, respectively. The Brillouin zone was sampled at the Gamma point. Projected density of states analysis was performed by using vaspkit tools in reference 24,50 and all the details for Bader charge analysis can be found in references.^25,26,51,52^

### 2.2 Photoabsorption TDDFT

Photoabsorption calculations were carried out with the complex polarizability TDDFT (pol-TDDFT) algorithm^45,53^ using the ADF engine of the AMS software.^54^ Such a method consists of a linear-response TDDFT formalism in the frequency domain.

The algorithm calculates the oscillator strength as

1 f=2ωε3Im[α̅]

where Im­[α̅] represents the imaginary part of the polarizability tensor averaged over all the orientations, ω is the photon energy, and ε is the imaginary part of the photon energy chosen to be equal to 0.075 eV. The calculations were done with the PW91 xc functional, the TZP basis set (with density fitting set appropriately optimized to be used with pol-TDDFT^55^), and the scalar ZORA^56^ approach. Such a choice has proven to be a convenient compromise between accuracy and computational economy: in particular, both xc functional^57^ and basis set performances^55^ have been assessed in previous work.

Besides the calculation of photoabsorption, we also analyze the results using tools specifically developed to study plasmonic systems in previous work: the Individual Component Map of Oscillator Strength plots (ICM-OS)^58^ and the fragment analysis.^59^ In the fragment analysis, we divided the whole system into two fragments, namely the metal cluster (M) and the surface (S). Hence, it is possible to decompose the total photoabsorption profile into a sum of four different contributions, namely M → M, M → S, S → M, and S → S.

## 3 Results and Discussion

### 3.1 Structural Models

In the first step, periodic slab models were built for the metal–TiO_2_ systems using the most stable rutile (110) among the low-index surfaces of rutile TiO_2_. A four/five-layer-thick model is used to reproduce the (110) surface (Figures 1 and S1). The surface primitive cell has been expanded by five/seven times, along the *x* direction and twice along the *y* direction, building 5 × 2 (see Figure 1 panels a and c) and 7 × 2 (see Figure 1 panel b) supercells, with the lateral size of the supercell around 15 × 13 Å^2^ (a and c in Figure 1) and 21 × 13 Å^2^ (b in Figure 1). The supercell contains a total of 260, 337, and 373 atoms, respectively, for a, c, and b in Figure 1. The tetrahedral M_20_ cluster was chosen due to its high symmetry (Td) and stability,^60^ and the nanorod M_37_ was chosen to maximize the optical response along a specific direction. Initial positions for the cluster adsorbed on TiO_2_ were chosen such that the metal cluster atoms in the bottom plane connect to bridging oxygen atoms of the TiO_2_ (110) surface in order to maximize the number of direct M–TiO_2_ interactions.

**Figure 1:**
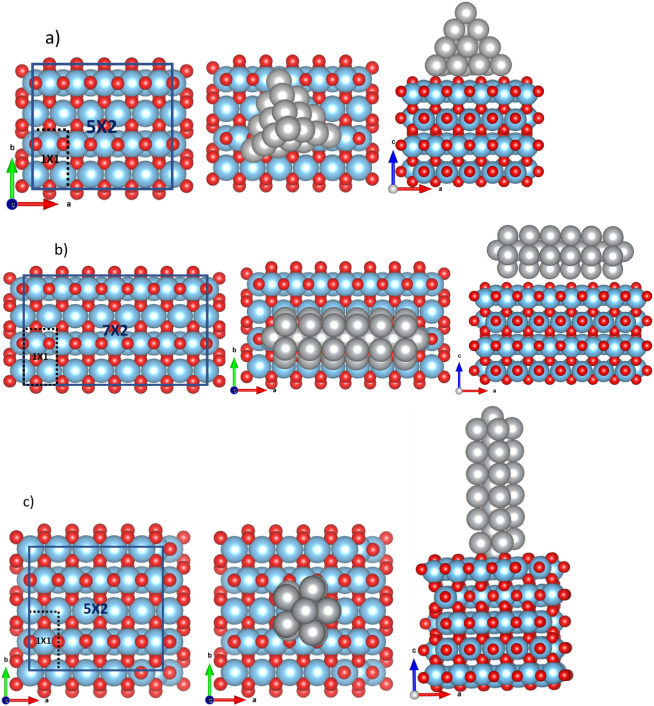
(a–c) Optimized geometries of M = Ag, Au, Cu metal clusters adsorbed on rutile TiO_2_ (110). Blue atoms: Ti, red atoms: O, grey atoms: M.

To be able to capture the local environment and interactions around the region of interest accurately, a suitable cluster model is then cut from the periodic slab, and oxygen dangling bonds are saturated for the TDDFT calculations. By selecting a cluster size that includes the essential atoms and bonding environment, we can approximate the electronic structure and optical properties of the larger system such as nanoparticles supported on TiO_2_. In addition, cluster models offer a balance between accuracy and computational cost. While they may slowly converge to capture the long-range effects or bulk behavior of the material, they provide insights into the local interactions and excitations that are crucial for understanding the plasmonic coupling effect in supported metal clusters on TiO_2_.

To avoid artifacts due to the employment of limited-size TiO_2_ clusters to model the surface, we used the saturation of dangling bond states approach to obtain suitable boundary conditions as demonstrated by Casarin et al.^61,62^ Three models have been adopted (Figure S1): the first is [Ti_54_O_155_M_20_H_137_]^+4/3^, M = Ag, Au, Cu; the second is [Ti_54_O_155_Ag_37_H_137_]^+7/3^; and the third is [Ti_58_O_160_Ag_37_H_136_]^+5/3^. We employed pseudohydrogen saturators, which are linked to O atoms with a nuclear charge *Z*_H_ = 2/3. The fractional total charge of the clusters was finally adjusted in order to obtain a closed-shell electron configuration, in order to run the TDDFT program, which requires this condition. The determination of the nuclear charge of the saturators was done as follows. In the rutile TiO_2_ structure, Ti atoms are 6-fold coordinated, while O ones are 3-fold coordinated. This means that each Ti shares its four valence electrons with six oxygens, thus contributing with 4/6 = 2/3 electrons to each metal–oxygen bond. Therefore, if we want to correctly saturate the cluster, we have to provide 2/3 electrons to each O dangling bond. This result can be achieved by using pseudohydrogen saturators with proper fractional nuclear charges. Notice that this embedding scheme has proven very efficient to simulate surface properties with rather small cluster sizes,^61^ as well as successful to simulate localized phenomena like core electron excitations in the simulation of NEXAFS spectra.^62^ In particular, this scheme is suitable for TiO_2_ due to the covalent nature of the Ti–O chemical bond, which prevents the employment of a simpler embedding scheme like the optimized point charges.

The interatomic O–H distance is taken to be equal to 1.0104 Å, obtained by energy minimization on OH_3_ pseudomolecules using the local density approximation (LDA) with the VWN exchange-correlation (xc) potential.

In summary, the slab structures were first optimized under periodic boundary conditions, keeping the two bottom atomic layers frozen while allowing the remaining atoms to relax, and then we cut out a cluster model that we saturated with pseudo-H bonds. Such saturation has been done automatically with a homemade program. The cluster model was not reoptimized.

### 3.2 Geometries, Energetics, and Electronic Structure

Although for the following plasmon photoabsorption calculations (next Section 3.3), we need only the ground-state geometry, we also checked the energetics and the electronic structures of the systems under study in order to be safely assured that the so obtained structures are realistic. Moreover, since there is a lot of active research in the community to describe the electronic structure of TiO_2_ bulk and surfaces of all its allotropic forms, we do not have the goal to give a contribution in this field but rather to have reliable geometries to study the plasmons, which are the main objective of the present work.

Table 1 presents the results of the optimized distances between the atoms of the metallic clusters (M) and the oxygen surface atoms (O) for the studied systems on rutile. Since upon adsorption, there are many nonequivalent new bonds that are formed, we report all the optimized distances in order to have an idea of the distribution of the bond lengths. In Table 1, the adsorption energies (*E*_ads_) for our systems calculated with the eq 2 are presented as well.

2 $$E_{ads}=E_{M/{TiO}_{2}}-E_{M}-E_{{TiO}_{2}}$$

**Table 1:** Interaction Distances (Å) between TiO_2_ Rutile (110) Surface and Cluster Metal Atoms after Geometry Optimization and Adsorption Energy (*E*_ads_)­^a^

System	Ag_20_TiO_2_	Au_20_TiO_2_	Cu_20_TiO_2_	Ag_37_TiO_2_ *z*	Ag_37_TiO_2_ *x*
*d*_M‑O_ (Å)	2.23	2.16	1.88	2.24 (twice)	2.19 (4 times)
	2.25	2.19	1.89	2.40	2.20 (twice)
	2.29	2.26	1.90 (twice)	2.41	
	2.31	2.30	1.92	2.48	
	2.48	2.57	2.04	2.50	
*E*_ads_ (eV)	–2.41	–0.54	–5.41	–3.67	–6.58

a *d*_M‑O_ are the distances between metal-surface oxygen atoms.

In eq 2, *E*_M_ negative values of *E*_ads_ correspond to stable adsorption geometries. When comparing the *E*_ads_ values for the M_20_TiO_2_ systems, a significant variation is observed. The most negative (highest absolute value) adsorption energy is observed for copper, followed by silver, and then the gold system. This suggests that the adsorption of copper exhibits greater energetic stability on the TiO_2_ surface compared with gold and silver. For Ag_37_TiO_2_ *x* (geometry along the *x*-axis), a highly negative *E*_ads_ value was observed in comparison with Ag_20_TiO_2_, obviously due to the high number of chemical bonds formed. These results indicate a strong adsorption of Ag_37_ clusters on the TiO_2_ surface in the *x* direction. In relation to the average interaction distances (*d*_M‑O_), it is possible to analyze the *E*_ads_ results. In fact, a shorter interaction distance suggests stronger adsorption, and it is interesting to see a correlation between the interaction distances and the *E*_ads_ values for each studied system.

The present calculations are consistent with a previous computational study where similar clusters (Au_38_ and Cu_38_) are adsorbed over the TiO_2_ surface.^43^ For Cu_38_, an adsorption energy (called interaction in ref 43) of about 6 eV is found, in excellent agreement with the present work. For Au_38_, a much weaker energy is found (about half), in qualitative agreement with the present work, where for gold, a much weaker adsorption energy is found as well.

A recent work^63^ reports interaction energies for Au_10_Cu_3_ mixed clusters on anatase reduced slabs in the range of −4 to −6 eV with a clear preference for the Cu atoms in contact with the oxide support. Of course, the nuclearity of the clusters is different and the reduction state of the support too, so although a direct comparison is difficult, the general trend is fully consistent with the present results.

It is worth noting that in the case of Ag_37_TiO_2_ *z*, the optimized geometry is very sensitive to the geometry initial guess. In fact, if the initial geometry is taken in order to have a large enough distance between the surface and the metal cluster, the relaxation is modest, and the metal cluster keeps its initial shape. On the other hand, if the initial geometry is too compressed, then the relaxation is stronger and the cluster structure is subject to a reconstruction near the surface; therefore, the shape of the cluster changes considerably. Since the goal of the present work is to study the effect of the rutile TiO_2_ surface on the plasmon of the metal cluster, we considered it more significant to take as a reference the geometrical model with the minimal distortion with respect to the free cluster. This will allow us to identify more clearly the mutual coupling between the surface and the plasmonic metal cluster.

The pDOS (partial density of states) analysis is reported in Figure 2. Starting with the pure TiO_2_ surface alone in Figure 2a (from ADF) and Figure 2g (from VASP), a band gap is observed, indicating the absence of available electronic states within a certain energy range. This band gap is typically characteristic of the semiconductor properties of TiO_2_. The band gap from the VASP calculation is larger than that from ADF because of the U parameter used in VASP. The observed difference in the band gap width between the pDOS from the GGA+*U* (VASP) calculation and the pure DFT (ADF) calculation reflects a well-known deficiency of the LDA and GGA xc potential in predicting accurate band gaps. The GGA+*U* method incorporates a correction for strong electron–electron interactions, particularly in materials with highly correlated electronic structures. This correction is intended to improve the description of localized electronic states and can have an impact on the energy and localization of these states, ultimately affecting the band gap width. The fact that the pDOS from the GGA+*U* calculation exhibits a larger band gap confirms that the inclusion of the *U* correction strengthens the effects of electronic correlation, resulting in a more accurate representation of the electronic properties of the material. This usually leads to a better alignment with experimental observations, particularly in systems with strong electron–electron interactions or localized electronic states. The DOS results are in agreement with previous results in the literature.^64^

**Figure 2:**
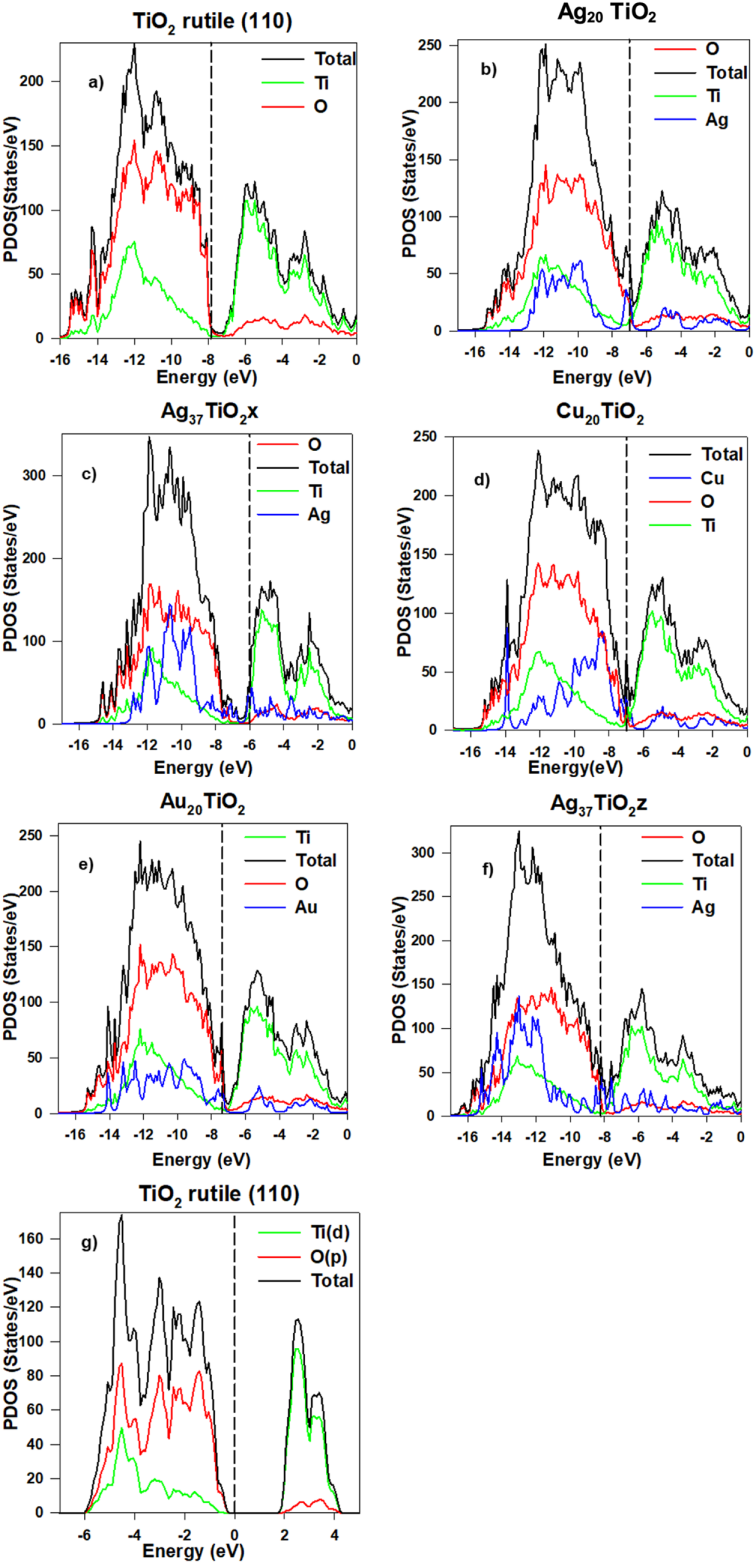
(a–g) Atom-projected density of states (PDOS) for different clusters shown for all atoms (black), Ti d-states (green), O p-states (red), and metal states (blue). Rutile TiO_2_ (110)-(a) results from ADF, whereas rutile TiO_2_ (110)-(g) results from VASP. The Fermi level is shown by short vertical dashed lines.

However, when a metal cluster is adsorbed onto this surface, the gap disappears as shown in Figure 2b–f, since the presence of metal nanoparticles onto the TiO_2_ surface creates additional electronic states that fill the gap. A detailed discussion about the Bader charges is reported in the Supporting Information.

### 3.3 Optical Properties

Figure 3 shows the photoabsorption profile for all of the systems investigated in this study. The Ag_20_ absorption spectrum is shown in Figure 3a. The spectrum of the free cluster Ag_20_ is characterized by a sharp and intense peak at 3.58 eV as a result of the plasmonic resonance. The nature of the plasmonic resonance is confirmed by the ICM-OS plots in Figure 4a, which is characterized by several off-diagonal intensity spots visible for the *X*, *Y*, and *Z* components. In fact, the plasmonic nature of a resonance is characterized by a collective nature where single excitations are strongly coupled: such couplings show up as off-diagonal spots in the ICM-OS plots. In the same Figure 3a, we can compare the spectrum of Ag_20_ with that of Ag_20_ adsorbed on the TiO_2_ surface with one *C*_3_ axis perpendicular to the surface, as well as that of the surface considered separately.

**Figure 3:**
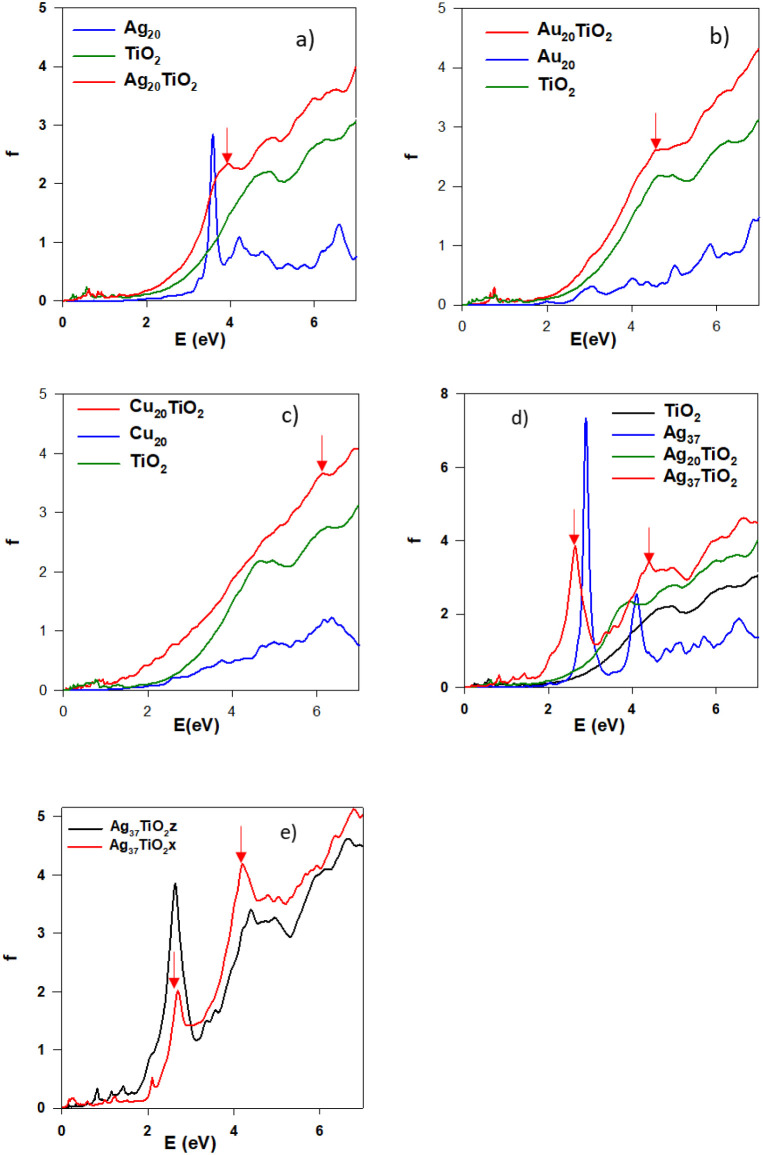
(a–e) TDDFT photoabsorption spectra of [Ti_54_O_155_M_20_H_137_]^+1/3^, [Ti_54_O_155_Ag_37_H_137_]^+4/3^, M_20_, Ag_37_^+^, and Ti_54_O_155_ calculated with the complex polarizability algorithm, M = Au, Ag, Cu. The arrows indicate the most prominent spectral features at 4.54, 3.94, and 6.14 eV for the three clusters M_20_TiO_2_, respectively. For the Ag_37_TiO_2_ *z* and Ag_37_TiO_2_ *x* features, see Table 2.

**Figure 4:**
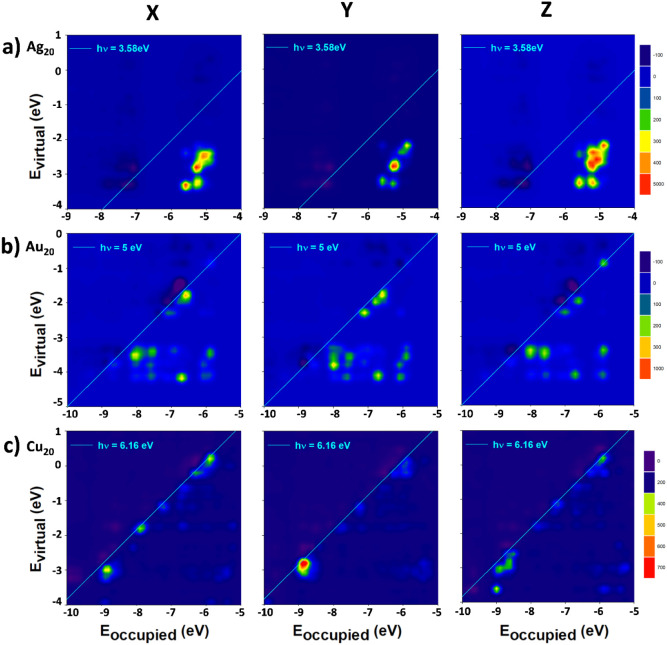
Two-dimensional ICM-OS plots for *X*, *Y*, and *Z* components of (a) Ag_20_, (b) Au_20_, and (c) Cu_20_.

Notice that, while for the calculations of the energetics in the previous section we employed periodic calculations, the TDDFT calculations of the spectra were carried out employing finite-size cluster models terminated by pseudohydrogens, as reported in Figure S1.

The spectrum of the adsorbed cluster is characterized by an absorption peak at 3.94 eV, while the isolated surface displays a peak at 4.65 eV. The adsorbed cluster peak can be attributed to the longitudinal plasmon (perpendicular to the surface). The nature of this plasmon has been confirmed by the ICM-OS plots in Figure S3a. The peak at 3.94 eV shows a very strong off-diagonal signal in the *Z* component only, confirming the existence of the longitudinal plasmon in the presence of the surface. There is also a weak signal in the *y* direction, but it is clear that the system is not plasmonic in this direction at this energy value because most of the spots lie on the diagonal, indicating only a marginal plasmonic nature.

The photoabsorption spectrum of Au_20_ is plotted in Figure 3b. This spectrum shows several low-intensity absorption peaks. In the same graph, the spectrum of the adsorbed cluster on the TiO_2_ surface shows a weak absorption peak at 4.58 eV. This indicates that Au_20_ and Au_20_TiO_2_ are less plasmonic than Ag_20_ and Ag_20_TiO_2_. This can be confirmed by the ICM-OS plots in Figure 4b. The characteristic peak of Au_20_ at 5 eV shows a mixture of signals in all three directions, while for the peak of the supported cluster at 4.54 eV in Figure S3b, all the spots lie on the diagonal. We can conclude that the system is not plasmonic, in fact, it is well-known that gold clusters start to show plasmonic behavior at much larger sizes.

In the case of Cu_20_, no signal of plasmon nature was observed (Figure 3c). A low peak was observed at 6.16 and 6.14 eV, respectively, for Cu_20_ and Cu_20_TiO_2_; and the ICM-OS plots in Figures 4c and S3c show only the spots on the diagonal. We can also conclude that Cu_20_ is not plasmonic.

In Figure 3d, the absorption spectrum of [Ag_37_]^+^ is reported. The free [Ag_37_]^+^ cluster spectrum is characterized by a sharp and intense peak at 2.88 eV followed by a less intense peak at 4.10 eV. Both features can be ascribed to plasmon resonances: the most intense at lower energy as the longitudinal plasmon and the less intense at higher energy to the transversal plasmon. This assignment is quite natural, since it is well-known that the plasmon resonance energy decreases and its intensity increases as the cluster size increases. In the present case, the size increases along the *C*_5_ axis of the [Ag_37_]^+^ metal cluster, which corresponds to the *z* direction (longitudinal). The nature of the plasmonic resonance is confirmed by the ICM-OS plots of Figure 5a: two off-diagonal spots with high intensity at 2.88 eV are apparent only for the *Z* component (longitudinal). The other two components (*X* and *Y*) are not plasmonic, since the spots lie all on the diagonal, with intensity 1 order of magnitude smaller than in the *Z* component. For the 4.10 eV energy point presented in Figure 5b, the plasmon resonance is present in the *X* and *Y* components, while in the *z* direction, no plasmon is observed. In the same Figure 3d, it is interesting to compare the spectrum of the [Ag_37_]^+^ cluster with that of the cluster adsorbed over the TiO_2_ surface with the *C*_5_ axis perpendicular to the surface, as well as the clean surface. In fact, we can say that the spectrum of the adsorbed cluster corresponds to the sum of the free TiO_2_ surface and the free cluster but with much more broadened plasmonic resonances. Moreover, as reported in Table 2, we observe that the energy of the longitudinal plasmon at 2.88 eV in the free cluster is slightly lowered at 2.63 eV as a consequence of the adsorption. This finding can be compared with a similar red shift observed in the experiment for gold nanoparticles with an average diameter of about 10 nm adsorbed over the TiO_2_ surface.^28^ In this case, the plasmon shifts from 522 nm for the free nanoparticles to 536 nm for the adsorbed ones, corresponding to a red shift of 0.06 eV. Although the elements are different and the sizes are much larger, the shift directions are consistent between theory and experiment.

**Figure 5:**
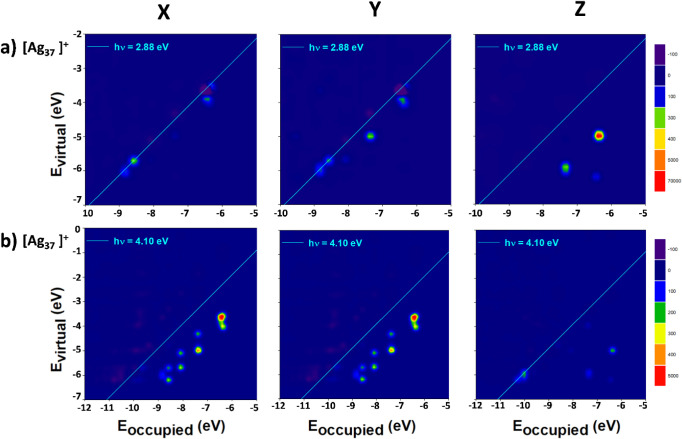
(a, b) Two-dimensional ICM-OS plots for *X*, *Y*, and *Z* components of [Ag_37_]^+^.

**Table 2:** Energy (*E*) and Intensity (*f*) for the Most Intense Absorption Peaks of Ag_37_, Ag_37_TiO_2_ *z*, and Ag_37_TiO_2_ *x*

System	*E* (eV)	*f*
Ag_37_	2.88	7.35
Ag_37_	4.10	2.55
Ag_37_TiO_2_ *z*	2.63	3.86
Ag_37_TiO_2_ *z*	4.39	3.40
Ag_37_TiO_2_ *x*	2.69	2.01
Ag_37_TiO_2_ *x*	4.19	4.18

On the other hand, for the transversal plasmon, the opposite behavior is found: the transversal plasmon at 4.10 eV in the free cluster shifts to 4.39 eV in the TiO_2_-supported cluster. This is a consequence of the Coulomb interaction between the induced dipoles: when the cluster and the surface are in the same direction with respect to the dipole, the interaction is between opposite charges; therefore, the energy is decreased. On the other hand, when the dipole is perpendicular to the surface-cluster direction, the interaction is between charges of the same sign and therefore the energy is increased. The comparison between the [Ag_37_]^+^ cluster adsorbed in two different orientations with respect to the TiO_2_ surface, perpendicular (*z*) or parallel (*x*) is reported in Figure 3e. In the parallel orientation, the low-energy longitudinal plasmon (just above 2 eV) is reduced in intensity, while the transversal plasmon (around 4 eV) is slightly amplified.

The mechanism of such energy and intensity modulation can be easily explained with simple arguments, as shown in Scheme 1. The situation a) corresponds to the longitudinal plasmon with Ag_37_ along *z*: the closer induced charges are near the surface and have opposite signs, promoting a red shift of the plasmon. Moreover, the transition dipoles are along the same direction, so we expect an intensity enhancement. Both consequences (red shift and intensity increase) were actually found for the longitudinal plasmon of TiO_2_Ag_37_ along *z*. The situation b) corresponds to the transversal plasmon with Ag_37_ along *z*: the closer induced charges are near the surface and have the same sign, promoting a blue shift of the plasmon. Moreover, the transition dipoles are along two different parallel directions, so we expect an intensity reduction. Both consequences (blue shift and intensity reduction) were actually found for the transversal plasmon of TiO_2_Ag_37_ along *z*. The situations c) and d) lead to the same conclusions as in a) and b), respectively, and are consistent with the previous findings of the transversal and longitudinal plasmon of TiO_2_Ag_37_ along *x*, respectively.

**Scheme 1:**
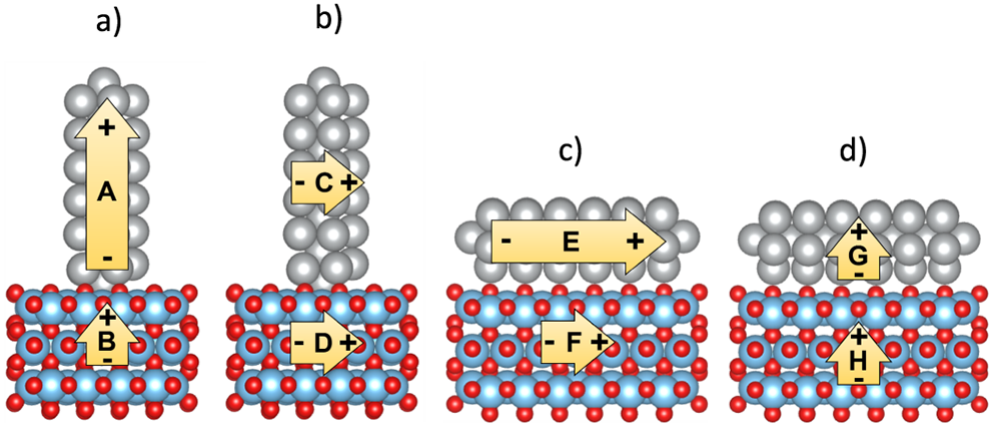
TiO_2_ Surface and the Ag_37_ Cluster along the *z* Direction in a) and b) and along the *x* Direction in c) and d), the Arrows Represent the Transition Electric Dipole Moment and +/– Designate the Induced Charged Due to the Transition Dipole; The Length of the Arrows Is Proportional with the Transition Electric Dipole Moment According to the Following Ratios: A:B = 5.9:1, A:C = 1.7:1, C:D = 1.18:1, A:E = 1:1, E:F = 5.9:1, E:G = 1.7:1, G:H = 1.18:1

In order to better analyze the plasmon interaction with the TiO_2_ surface, we can analyze the ICM-OS plots of Figure 6, where the Ag_37_ cluster is perpendicular to the surface. The feature at 2.63 eV gives a very strong off-diagonal signal only in the *z*-component, confirming the existence of the longitudinal plasmon also in the presence of the surface. On the other hand, the feature at 4.39 eV displays plasmonic behavior only along the *x* and *y* directions (transversal plasmon), while along the *z* direction, most of the spots lie on the diagonal, indicating a very low plasmonic nature. Quite interestingly, the opposite pattern is observed in the Ag_37_TiO_2_ *x* geometry (see Figure 7): in this case, the ICM-OS at 2.69 eV shows plasmonic behavior only along the *x* direction (now the longitudinal plasmon), while at 4.19 eV, the plasmonic behavior is found along the two transversal directions (*y* and *z*).

**Figure 6:**
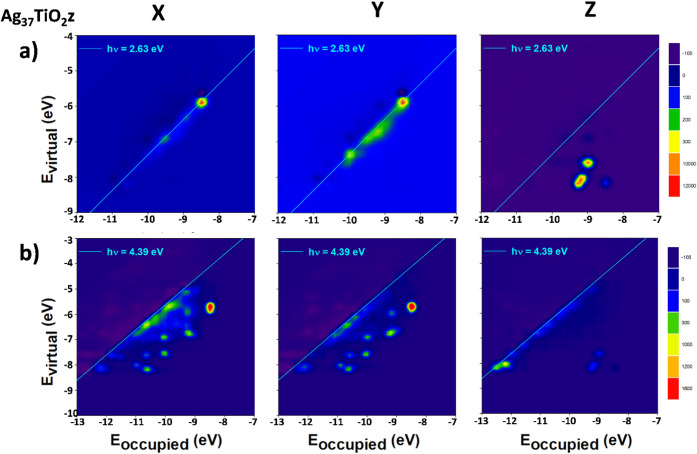
(a, b) Two-dimensional ICM-OS plots for *X*, *Y*, and *Z* components of [Ti_54_O_155_Ag_37_H_137_]^+4/3^ at the energy corresponding to the absorption maxima (see values in Figure 4).

**Figure 7:**
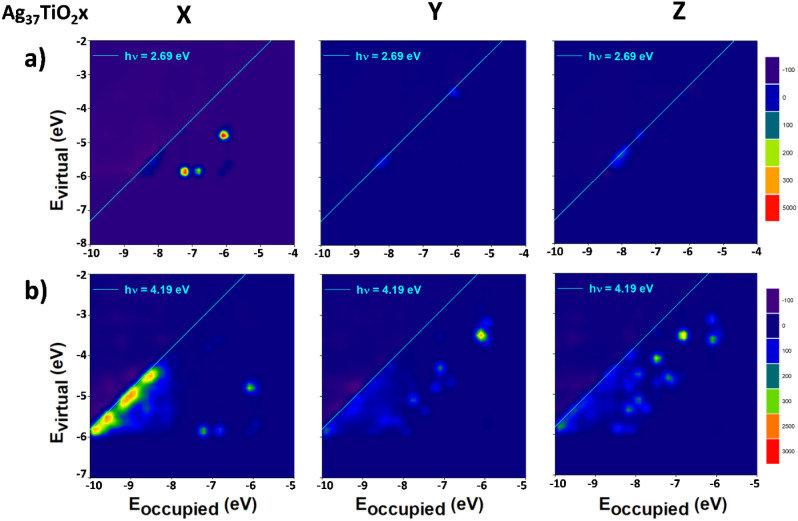
(a, b) Two-dimensional ICM-OS plots for *X*, *Y*, and *Z* components of [Ti_58_O_160_Ag_37_H_136_]^+5/3^ at the energy corresponding to the absorption maxima (see values in Figure 4).

### 3.4 Fragment Analysis and Transition Densities

The fragment analysis of the photoabsorption spectra of the systems investigated is reported in Figure 8. The absorption intensity is split according to the fragments involved in the transition. For such systems, it is natural to split the whole systems in two fragments: the TiO_2_ surface (S) and the metal cluster (M). Starting with the Ag_20_TiO_2_ geometry, the longitudinal plasmon at 3.94 eV is mainly due to the contribution of metal → surface transitions, which is supported by a background with prominent S → S nature. This indicates that the plasmonic transition is injecting a small charge from the cluster to the surface. Going along the series Au_20_TiO_2_ and Cu_20_TiO_2_ reported in Figure 8b and d, the S → S smooth contribution plays the major role, while the M → S contribution is less important, but while it supports a weak structure in Au_20_TiO_2_, it is completely structureless in Cu_20_TiO_2_. For the Ag_37_TiO_2_ *z* geometry, it is apparent that the longitudinal plasmon at 2.88 eV is mainly due to the S → M contribution. This indicates that the transition corresponds to a partial charge transfer from the surface to the cluster. On the other hand, for the Ag_37_TiO_2_ *x* geometry, the longitudinal plasmon at 2.65 eV is not only quenched but also is contributed mainly by M → M transitions. This is easily rationalized since the longitudinal plasmon in Ag_37_TiO_2_ *x* geometry is parallel to the surface, and since it does not point toward the surface, it is less coupled to it, so it keeps its M → M nature. Finally, in Figure 9, we consider the transition densities calculated for the various geometries and energies: it is well apparent that while the metal cluster in general is able to keep its plasmonic shape, which consists of a dipole charge distribution, the TiO_2_ surface displays a very irregular density distribution. In any case, the longitudinal plasmon is the most apparent, as in d) *z* component and e) *x* component for Ag_37_TiO_2_ systems. It is worth noting that in d) *z*, the induced density in the surface acquires a more structured, less irregular shape. This is consistent with the mechanism of plasmon enhancement already observed and discussed in the previous Section 3.2. On the other hand in e) *x*, although the surface-induced density is irregular, we observe that there is an accumulation of density in the interface between the cluster and the surface, indicating at least some interaction, due to the strong longitudinal plasmon. In fact, if we consider the weaker transversal plasmon in e) *y*, it is apparent that there is no charge accumulation in the interface.

**Figure 8:**
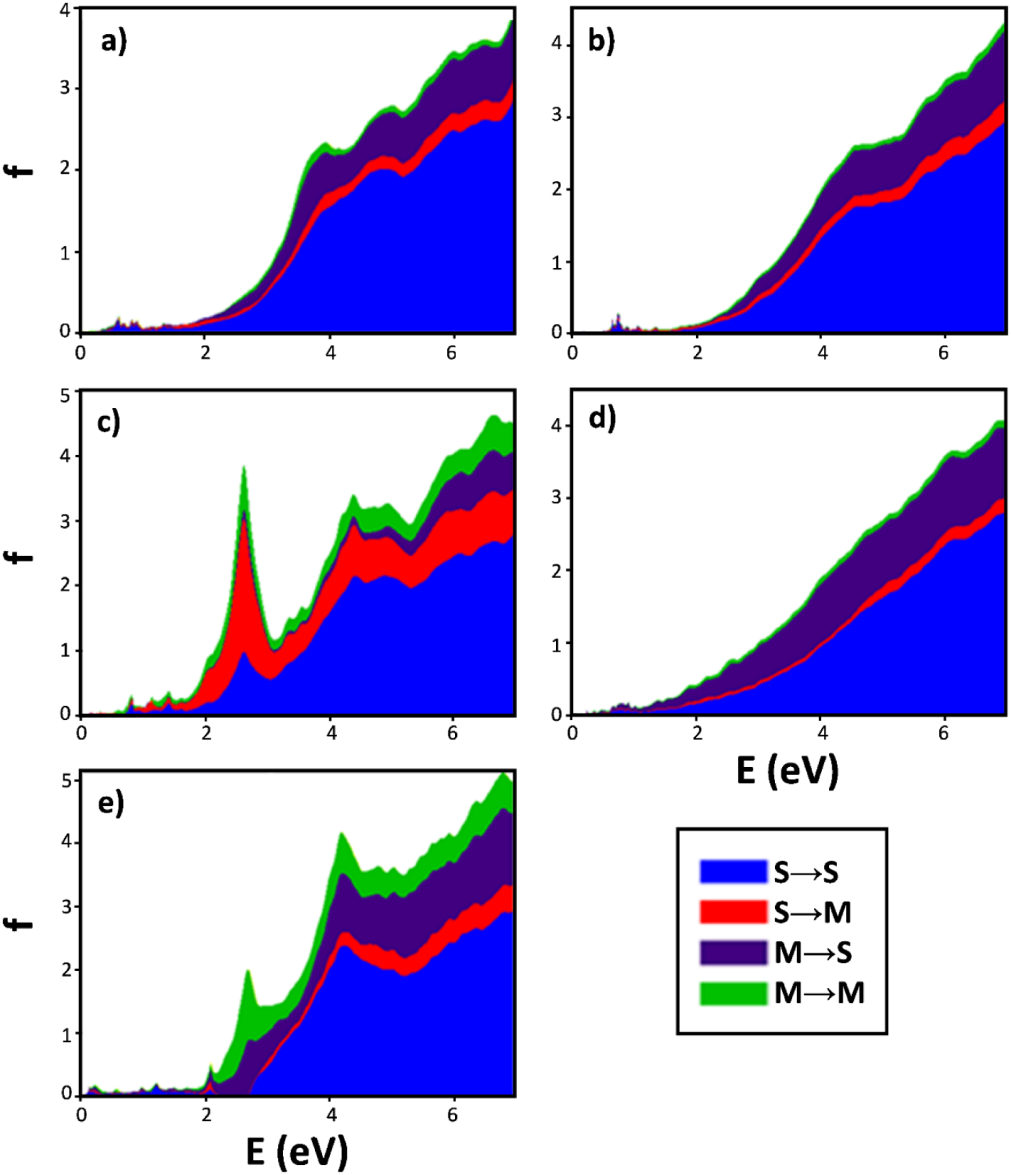
Fragment analysis of the photoabsorption spectra of [Ti_54_O_155_M_20_H_137_]^+1/3^ and [Ti_54_O_155_Ag_37_H_137_]^+4/3^, M = Ag, Au, Cu. (a) Ag_20_TiO_2_. (b) Au_20_TiO_2_. (c) Ag_37_TiO_2_ *z*. (d) Cu_20_TiO_2_. (e) Ag_37_TiO_2_ *x*.

**Figure 9:**
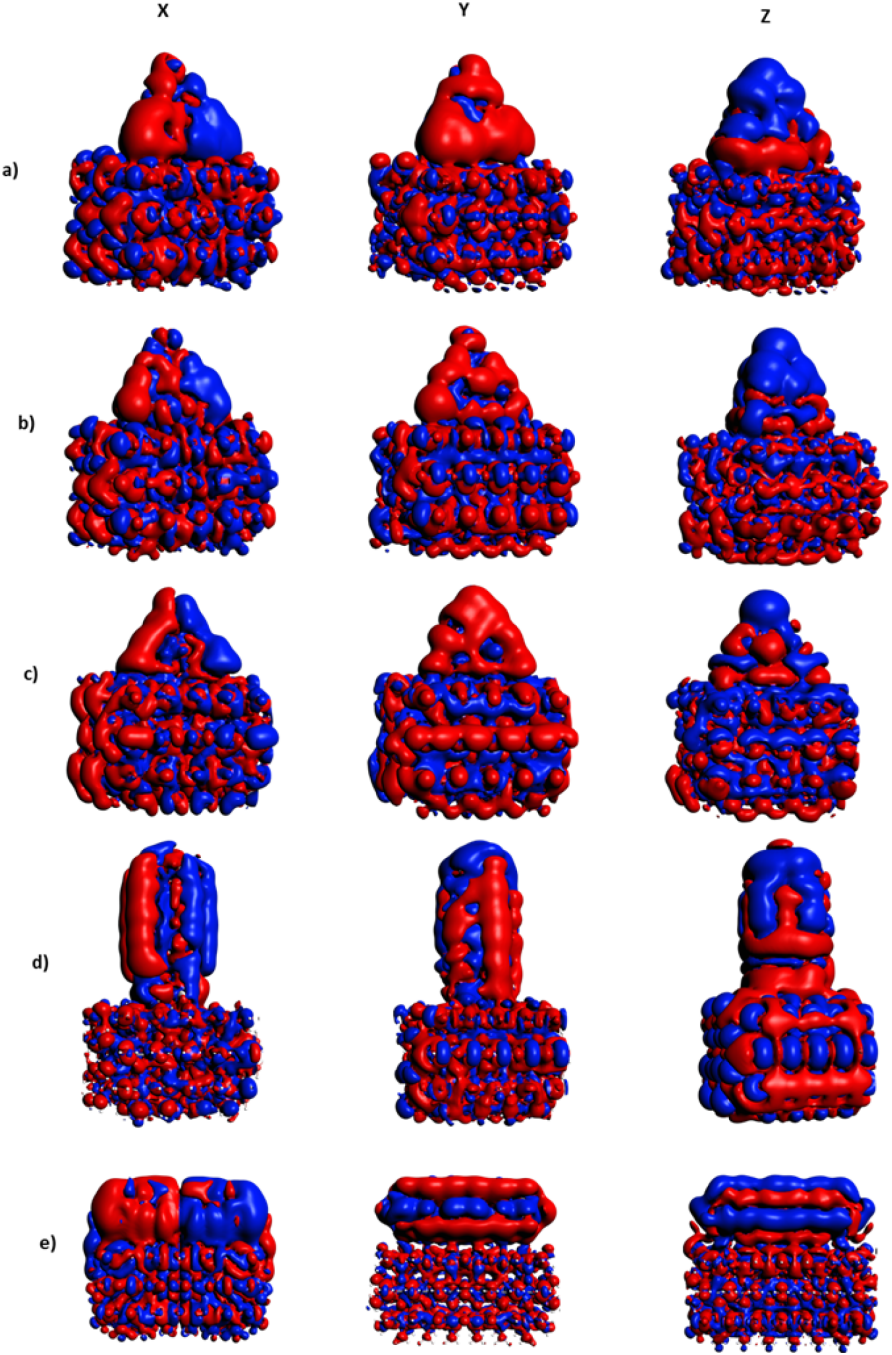
Transition densities calculated with the complex polarizability algorithm at the energy corresponding to the absorption maxima (see values in Figure 3): *X*, *Y*, and *Z*-dipole components of (a)–(c) [Ti_54_O_155_M_20_H_137_]^+1/3^ with M= Ag, Au, Cu, respectively, (d) [Ti_54_O_155_Ag_37_H_137_] ^+4/3^, and (e) [Ti_58_O_160_Ag_37_H_136_]^+5/3^.

### 3.5 Effects of Dispersion Forces

Up to now, we considered only the GGA functional since it is the default choice for the calculation of photoabsorption spectra and is therefore useful for comparison with previous calculations. On the other hand, for such systems, it is likely important to include dispersion forces as well, which are supposed to be relatively large especially regarding the interaction between the metal cluster and the TiO_2_ surface. In order to check the most critical system, we opted for Ag_37_ along *z* since it is the system for which we expect the largest effect. In order to understand the different role of the dispersion forces, we employed the Grimme D3-BJ functional^65,66^ to perform the following analysis: a) a rigid translation of the metal cluster toward the surface, but relaxing neither the surface nor the cluster; b) starting from the PBE-optimized slab geometry, a reoptimization adding D3-BJ with VASP, then cutout the cluster and saturating; c) a full geometry optimization of the bulk rutile structure with VASP D3-BJ, then building and reoptimizing the slab, followed by the construction of the finite-size cluster model. Then, for each of the previous structural models, we calculated the photoabsorption spectra, in order to compare them with the original one calculated at the optimized GGA geometry. In Figure S4 of the Supporting Information, we report the D3-BJ energetic profile of the rigid translation of the Ag_37_ fragment toward the TiO_2_ surface. The starting point is the GGA-PW91 equilibrium geometry with a Ag–O distance of 2.239 Å. As expected, the inclusion of the D3-BJ correction promotes stabilization for shorter distances; in fact, the minimum energy is obtained at 2.166 Å, giving a moderate shortening of only 0.073 Å. Also, the energetic gain is modest, being only 2 kcal/mol. In the following Figure S5 of the Supporting Information, we compare the original TDDFT spectrum calculated at the GGA-PW91 level (black curve) with the one calculated at the optimized distance by rigid translation (blue curve): the two spectra are almost perfectly overlapped indicating that the modest shortening does not have any consequence on the spectrum. In order to check in a more complete way the effect of the dispersion forces, we also calculated the spectrum at the optimized geometry of the finite-size cluster model (red curve in Figure S5) as well as after the geometry optimization of the cluster adsorbed over the surface by a periodic method (VASP) followed by the construction of the finite-size cluster model (green curve in Figure S5). For both cases, the result is only very slightly different with respect to the original model (black curve) with a shift of only 0.1 eV. From such an analysis, we can conclude by saying that although the dispersion forces have an important role to define the geometry of the system, their impact on the photoabsorption spectra, and on plasmons in particular, is negligible. This also corroborates our previous analysis based on the GGA-PW91 functional.

## 4 Conclusions

Density functional theory (DFT) and time-dependent density functional theory (TDDFT) have been employed to study the plasmonic coupling effects of silver, gold, and copper clusters supported on TiO_2_. The adsorption of metal nanoparticles on the surface of TiO_2_ changes the electronic properties of the system, resulting in the disappearance of the bandgap. Using DFT and polTDDFT calculations, we characterized the photoabsorption spectra of TiO_2_/metal NP systems.

In addition, we used analysis tools, including ICM-OS plots, fragment analysis, and transition density, to deepen our understanding of the studied systems. These spectra revealed absorption peaks corresponding to electronic transitions induced by metallic nanoparticles. The analysis of photoabsorption spectra allowed us to identify surface plasmons and quantify their contribution to the absorption of visible light. Using ICM-OS plots, we evaluated the strength of the plasmonic coupling between metal nanoparticles and TiO_2_, allowing the identification of favorable configurations for the plasmonic coupling. This approach highlighted the specific effects of silver nanoparticles on visible-light absorption. Finally, detailed information on the charge-transfer mechanisms in the studied systems was revealed by transition density analysis. By identifying the main electronic states involved in these processes, we could understand how plasmonic coupling influences charge carrier dynamics and improve the photoabsorption performance.

In conclusion, our work used a combination of advanced theoretical calculations and original analysis techniques such as ICM-OS plots, fragment analysis, and transition density to study the photoabsorption spectra of metal nanoparticles on TiO_2_ and to provide valuable information on underlying mechanisms and structure–property relationships. These results confirmed the silver cluster as the most plasmonic and opened up promising avenues for the design of improved TiO_2_/silver NP photocatalytic materials. However, it is important to note that our results are based on theoretical calculations, and experimental validation is required. Further experimental studies, such as spectroscopic measurements and photocatalytic tests, are required to confirm and supplement our theoretical results. It should also be interesting to consider more realistic systems, such as the presence of O vacancies and larger metal clusters, in a future study.

## Supplementary Material



## Notes

H-saturated models, Bader charge analysis, and additional ICM-OS analysis; Geometries (XYZ format) of the systems considered in this study: TiO_2_, Ag_20_, Au_20_, Cu_20_, Ag_37_^+^, Ag_20_TiO_2_, Au_20_TiO_2_, Cu_20_TiO_2_, Ag_37_TiO_2_ *x*, Ag_20_TiO_2_ *z* (PDF)

## References

[1] Ahmad I., Zou Y., Yan J., Liu Y., Shukrullah S., Naz M. Y., Hussain H., Khan W. Q., Khalid N. R. (2023). Semiconductor
Photocatalysts: A Critical Review Highlighting the Various Strategies
to Boost the Photocatalytic Performances for Diverse Applications. Adv. Colloid Interface Sci.

[2] Wang Z., Lin Z., Shen S., Zhong W., Cao S. (2021). Advances in Designing
Heterojunction Photocatalytic Materials. Chin.
J. Catal.

[3] Lin Z., Wang C., Wang Z., Liu Q., Le C., Lin B., Chen S. (2019). The Role of Conductivity and Phase Structure in Enhancing
Catalytic Activity of CoSe for Hydrogen Evolution Reaction. Electrochim. Acta.

[4] Zhao C., Zhou A., Dou Y., Zhou J., Bai J., Li J.-R. (2021). Dual MOFs Template-Directed
Fabrication of Hollow-Structured Heterojunction
Photocatalysts for Efficient CO2 Reduction. Chem. Eng. J.

[5] Zhang R., Elzatahry A. A., Al-Deyab S. S., Zhao D. (2012). Mesoporous Titania:
From Synthesis to Application. Nano Today.

[6] Ni M., Leung M. K. H., Leung D. Y. C., Sumathy K. (2007). A Review and Recent
Developments in Photocatalytic Water-Splitting Using TiO2 for Hydrogen
Production. Renewable Sustainable Energy Rev.

[7] Carp O. (2004). Photoinduced
Reactivity of Titanium Dioxide. Prog. Solid
State Chem.

[8] Mor G. K., Varghese O. K., Paulose M., Shankar K., Grimes C. A. (2006). A Review
on Highly Ordered, Vertically Oriented TiO2 Nanotube Arrays: Fabrication,
Material Properties, and Solar Energy Applications. Sol. Energy Mater. Sol. Cells.

[9] Bruce P. G., Scrosati B., Tarascon J. (2008). Nanomaterials
for Rechargeable Lithium
Batteries. Angew. Chem., Int. Ed.

[10] Liu L., Gu X., Sun C., Li H., Deng Y., Gao F., Dong L. (2012). In Situ Loading
of Ultra-Small Cu2O Particles on TiO2 Nanosheets
to Enhance the Visible-Light Photoactivity. Nanoscale.

[11] Kong M., Li Y., Chen X., Tian T., Fang P., Zheng F., Zhao X. (2011). Tuning the
Relative Concentration Ratio of Bulk Defects to Surface
Defects in TiO_2_ Nanocrystals Leads to High Photocatalytic
Efficiency. J. Am. Chem. Soc.

[12] Yang H. G., Sun C. H., Qiao S. Z., Zou J., Liu G., Smith S. C., Cheng H. M., Lu G. Q. (2008). Anatase
TiO2 Single
Crystals with a Large Percentage of Reactive Facets. Nature.

[13] Tian Y., Tatsuma T. (2005). Mechanisms and Applications
of Plasmon-Induced Charge
Separation at TiO_2_ Films Loaded with Gold Nanoparticles. J. Am. Chem. Soc.

[14] Hamad H., Elsenety M. M., Sadik W., El-Demerdash A.-G., Nashed A., Mostafa A., Elyamny S. (2022). The Superior
Photocatalytic
Performance and DFT Insights of S-Scheme CuO@TiO2 Heterojunction Composites
for Simultaneous Degradation of Organics. Sci.
Rep.

[15] Lin L., Bi X., Gu Y., Wang F., Ye J. (2021). Surface-Enhanced Raman
Scattering Nanotags for Bioimaging. J. Appl.
Phys.

[16] Prakash J., Sun S., Swart H. C., Gupta R. K. (2018). Noble Metals-TiO2 Nanocomposites:
From Fundamental Mechanisms to Photocatalysis, Surface Enhanced Raman
Scattering and Antibacterial Applications. Appl.
Mater. Today.

[17] Kumar A., Choudhary P., Kumar A., Camargo P. H. C., Krishnan V. (2022). Recent Advances
in Plasmonic Photocatalysis Based on TiO_2_ and Noble Metal
Nanoparticles for Energy Conversion, Environmental Remediation, and
Organic Synthesis. Small.

[18] Cushing S. K., Li J., Meng F., Senty T. R., Suri S., Zhi M., Li M., Bristow A. D., Wu N. (2012). Photocatalytic Activity Enhanced
by Plasmonic Resonant Energy Transfer from Metal to Semiconductor. J. Am. Chem. Soc.

[19] Meng A., Zhang L., Cheng B., Yu J. (2019). Dual Cocatalysts in
TiO_2_ Photocatalysis. Adv. Mater.

[20] Albiter E., Hai Z., Alfaro S., Remita H., Valenzuela M. A., Colbeau-Justin C. (2013). A Comparative
Study of Photo-Assisted Deposition of
Silver Nanoparticles on TiO_2_. J.
Nanosci. Nanotechnol.

[21] He J., Kumar A., Khan M., Lo I. M. C. (2021). Critical Review
of Photocatalytic Disinfection of Bacteria: From Noble Metals- and
Carbon Nanomaterials-TiO2 Composites to Challenges of Water Characteristics
and Strategic Solutions. Sci. Total Environ.

[22] Sankar S., Gopchandran K. G. (2013). Rutile
TiO2­(101) Based Plasmonic Nanostructures. Ceram.
Int.

[23] Yoo S. M., Rawal S. B., Lee J. E., Kim J., Ryu H.-Y., Park D.-W., Lee W. I. (2015). Size-Dependence
of Plasmonic Au Nanoparticles
in Photocatalytic Behavior of Au/TiO 2 and Au@SiO 2/TiO 2. Appl. Catal. Gen.

[24] Ma J., Wang J., Gao S. (2022). Effect of Light Polarization on Plasmon-Induced
Charge Transfer. J. Chem. Phys.

[25] Ma J., Gao S. (2019). Plasmon-Induced Electron–Hole
Separation at the Ag/TiO _2_ (110) Interface. ACS Nano.

[26] Ma X.-C., Dai Y., Yu L., Huang B.-B. (2016). Energy Transfer in Plasmonic Photocatalytic
Composites. Light Sci. Appl.

[27] Yu Y., Dong X., Chen P., Geng Q., Wang H., Li J., Zhou Y., Dong F. (2021). Synergistic Effect of Cu Single Atoms
and Au–Cu Alloy Nanoparticles on TiO _2_ for Efficient
CO _2_ Photoreduction. ACS Nano.

[28] Lin Z., Wang X., Liu J., Tian Z., Dai L., He B., Han C., Wu Y., Zeng Z., Hu Z. (2015). On the Role
of Localized Surface Plasmon Resonance in UV-Vis Light Irradiated
Au/TiO_2_ Photocatalysis Systems: Pros and Cons. Nanoscale.

[29] Yan J., Wu G., Guan N., Li L. (2013). Synergetic Promotion of the Photocatalytic
Activity of TiO2 by Gold Deposition under UV-Visible Light Irradiation. Chem. Commun.

[30] Kowalska E., Mahaney O. O. P., Abe R., Ohtani B. (2010). Visible-Light-Induced
Photocatalysis through Surface Plasmon Excitation of Gold on Titania
Surfaces. Phys. Chem. Chem. Phys.

[31] Giannini V., Fernández-Domínguez A. I., Heck S. C., Maier S. A. (2011). Plasmonic
Nanoantennas: Fundamentals and Their Use in Controlling the Radiative
Properties of Nanoemitters. Chem. Rev.

[32] Lettieri S., Pavone M., Fioravanti A., Santamaria Amato L., Maddalena P. (2021). Charge Carrier Processes and Optical
Properties in
TiO2 and TiO2-Based Heterojunction Photocatalysts: A Review. Materials.

[33] Cushing S. K., Li J., Bright J., Yost B. T., Zheng P., Bristow A. D., Wu N. (2015). Controlling Plasmon-Induced Resonance Energy Transfer and Hot Electron
Injection Processes in Metal@TiO_2_ Core–Shell Nanoparticles. J. Phys. Chem. C.

[34] Zhang Z., Zhang C., Zheng H., Xu H. (2019). Plasmon-Driven Catalysis
on Molecules and Nanomaterials. Acc. Chem. Res.

[35] Abdelfattah I., El-Shamy A. M. (2024). A Comparative Study for Optimizing Photocatalytic Activity
of TiO2-Based Composites with ZrO2, ZnO, Ta2O5, SnO, Fe2O3, and CuO
Additives. Sci. Rep.

[36] Jameel M. H., Wang H., Du J., Kousar S., Bin Mayzan M. Z. H. (2025). A Comparative
Experimental and Theoretical DFT Study of Hydrothermally Synthesized
Transition Metal Oxides (V2O5 and TiO2) Nanomaterials for Photocatalytic
Application. J. Inorg. Organomet. Polym. Mater.

[37] Eidsvåg H., Bentouba S., Vajeeston P., Yohi S., Velauthapillai D. (2021). TiO2 as a
Photocatalyst for Water Splitting—An Experimental and Theoretical
Review. Molecules.

[38] Du X., Huang Y., Pan X., Han B., Su Y., Jiang Q., Li M., Tang H., Li G., Qiao B. (2020). Size-Dependent Strong Metal-Support Interaction in
TiO_2_ Supported Au Nanocatalysts. Nat. Commun.

[39] Lee K.-S., El-Sayed M. A. (2006). Gold and
Silver Nanoparticles in Sensing and Imaging:
Sensitivity of Plasmon Response to Size, Shape, and Metal Composition. J. Phys. Chem. B.

[40] Noguez C. (2007). Surface Plasmons
on Metal Nanoparticles: The Influence of Shape and Physical Environment. J. Phys. Chem. C.

[41] Volokh M., Mokari T. (2020). Metal/Semiconductor
Interfaces in Nanoscale Objects:
Synthesis, Emerging Properties and Applications of Hybrid Nanostructures. Nanoscale Adv.

[42] Kale M. J., Christopher P. (2015). Plasmons at the Interface. Science.

[43] Dhifallah M., Iachella M., Dhouib A., Di Renzo F., Loffreda D., Guesmi H. (2019). Support Effects Examined by a Comparative Theoretical
Study of Au, Cu, and CuAu Nanoclusters on Rutile and Anatase Surfaces. J. Phys. Chem. C.

[44] Iachella M., Wilson A., Naitabdi A., Bernard R., Prévot G., Loffreda D. (2016). Promoter Effect of
Hydration on the Nucleation of Nanoparticles:
Direct Observation for Gold and Copper on Rutile TiO _2_ (110). Nanoscale.

[45] Baseggio O., Fronzoni G., Stener M. (2015). A New Time
Dependent Density Functional
Algorithm for Large Systems and Plasmons in Metal Clusters. J. Chem. Phys.

[46] Kresse G., Furthmüller J. (1996). Efficient
Iterative Schemes for *Ab Initio* Total-Energy Calculations
Using a Plane-Wave Basis Set. Phys. Rev. B.

[47] Perdew J. P., Burke K., Ernzerhof M. (1996). Generalized Gradient Approximation
Made Simple. Phys. Rev. Lett.

[48] Dudarev S. L., Botton G. A., Savrasov S. Y., Humphreys C. J., Sutton A. P. (1998). Electron-energy-loss spectra and
the structural stability
of nickel oxide: An LSDA+U study. Phys. Rev.
B.

[49] Long R., Prezhdo O. V. (2014). Instantaneous Generation
of Charge-Separated State
on TiO _2_ Surface Sensitized with Plasmonic Nanoparticles. J. Am. Chem. Soc.

[50] Wang, V. ; Xu, N. ; Liu, J. C. ; Tang, G. ; Geng, W.-T. VASPKIT: A User-Friendly Interface Facilitating High-Throughput Computing and Analysis Using VASP Code. arXiv 2019.

[51] Sanville E., Kenny S. D., Smith R., Henkelman G. (2007). Improved Grid-Based
Algorithm for Bader Charge Allocation. J. Comput.
Chem.

[52] Tang W., Sanville E., Henkelman G. (2009). A Grid-Based Bader Analysis Algorithm
without Lattice Bias. J. Phys.: Condens. Matter.

[53] Baseggio O., Toffoli D., Fronzoni G., Stener M., Sementa L., Fortunelli A. (2016). Extension
of the Time Dependent Density Functional
complex polarizability algorithm to circular dichroism: implementation
and applications to Ag_8_ and Au_38_(SC_2_H_4_C_6_H_5_)_24_. J. Phys. Chem. C.

[54] Baerends E. J. (2025). The Amsterdam Modeling Suite. J. Chem. Phys.

[55] Medves M., Fronzoni G., Stener M. (2022). Optimization
of density fitting auxiliary
Slater-type basis functions for time-dependent density functional
theory *J*. Comput. Chem.

[56] Wang F., Ziegler T., van Lenthe E., van Gisbergen S., Baerends E. J. (2005). The calculation of excitation energies
based on the
relativistic two-component zeroth-order regular approximation and
time-dependent density-functional with full use of symmetry. J. Chem. Phys.

[57] Baseggio O., De Vetta M., Fronzoni G., Toffoli D., Stener M., Sementa L., Fortunelli A. (2018). Time-dependent
density-functional
study of the photoabsorption spectrum of Au_25_(SC_2_H_4_C_6_H_5_)_18_ anion: Validation
of the computational protocol. Int J. Quantum
Chem.

[58] Theivendran S., Chang L., Mukherjee A., Sementa L., Stener M., Fortunelli A., Dass A. (2018). Principles of Optical Spectroscopy
of Aromatic Alloy Nanomolecules: Au_36–x_Ag_x_(SPh-tBu)_24_. J. Phys. Chem. C.

[59] Sementa L., Barcaro G., Baseggio O., De Vetta M., Dass A., Aprà E., Stener M., Fortunelli A. (2017). Ligand-Enhanced
Optical Response of Gold Nanomolecules and Its Fragment Projection
Analysis: The Case of Au_30_(SR)_18_. J. Phys. Chem. C.

[60] Aikens C. M., Li S., Schatz G. C. (2008). From Discrete Electronic
States to Plasmons: TDDFT
Optical Absorption Properties of Ag*
_n_
* (*n* = 10, 20, 35, 56, 84, 120) Tetrahedral Clusters. J. Phys. Chem. C.

[61] Casarin M., Maccato C., Vittadini A. (1999). Density Functional
Studies of Molecular
Chemisorption on TiO_2_. Appl. Surf.
Sci.

[62] De
Francesco R., Stener M., Fronzoni G. (2011). Computational Investigation
of the L2,3-Edge Spectra of Bulk and (110) Surface of Rutile TiO_2_. Surf. Sci.

[63] Araiza D. G., Hellmer A., Ramírez-Cruz I., Lares-Rangel L. A., Portales-Martínez B., Calatayud M., Torres A., Zanella R. (2026). Integrating experiments and theory:
In-situ spectroscopic and DFT study of propane oxidation over AuCu/TiO_2_ catalysts. Chem. Eng. J.

[64] Griego C. D., Saravanan K., Keith J. A. (2019). Benchmarking Computational Alchemy
for Carbide, Nitride, and Oxide Catalysts. Adv.
Theory Simul.

[65] Grimme S., Antony J., Ehrlich S., Krieg H. (2010). A consistent
and accurate
ab initio parametrization of density functional dispersion correction
(DFT-D) for the 94 elements H-Pu. J. Chem. Phys.

[66] Grimme S., Ehrlich S., Goerigk L. (2011). Effect of the Damping Function in
Dispersion Corrected Density Functional Theory. J. Comput. Chem.

