# Supplementary material for: Plasmonic Coupling Effects in Metal Clusters Supported over TiO2: A Theoretical Study
Source: J Phys Chem C Nanomater Interfaces. 2026 May 21;130(22):7649–63. doi: 10.1021/acs.jpcc.5c08085 (PMC13244799; doi:10.1021/acs.jpcc.5c08085)
Supplement: Supplementary file 1 [file jp5c08085_si_001.pdf]

SUPPORTING INFORMATION FOR:

Plasmonic Coupling Effects in Metal Clusters Supported over TiO_2: a Theoretical Study

Parfaite Senoume Senou[1], Monica Calatayud[1]*

[1] *Sorbonne Universite, 4, Pl. Jussieu, 75252 Paris, France.*

Ardhmeri Alija[2], Pierpaolo D'Antoni[2], Daniele Toffoli[2] , Rilinda Plakaj[2] and Mauro Stener[2]*

[2]*Dipartimento di Scienze Chimiche e Farmaceutiche, Università di Trieste, Via Giorgieri 1, 34127 Trieste, Italy*

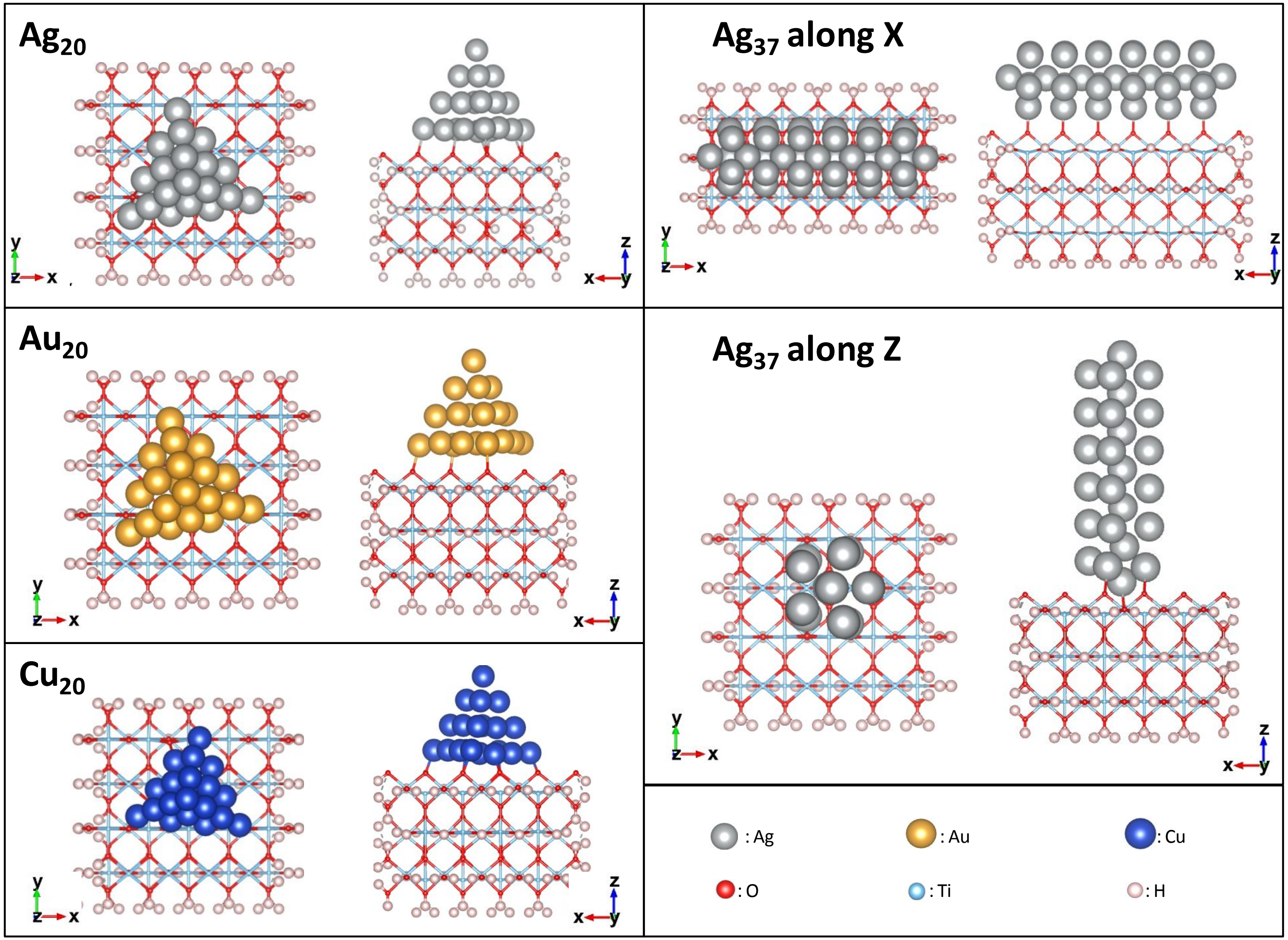

Ag$_{20}$
Au$_{20}$
Cu$_{20}$
Ag$_{37}$ along X
Ag$_{37}$ along Z
: Ag
: Au
: Cu
: O
: Ti
: H

Figure S1. Left, H-saturated models with Z_H=2/3 of M=Ag, Au, Cu TiO_2(110)-supported metal clusters used for the calculation of photoabsorption spectra with pol-TDDFT $[Ti_{54}O_{155}M_{20}H_{137}]^{+1/3}$ Right, Ag_{37} clusters $[Ti_{58}O_{160}Ag_{37}H_{136}]^{+5/3}$ geometry along x, and $[Ti_{54}O_{155}Ag_{37}H_{137}]^{+4/3}$ along z.

Bader charges analysis

The Bader charges results obtained in the studied systems reveal different charges for each type of atom. The calculated atomic charges are shown in table S1. These results correctly reproduce a difference in charges between titanium (positive) and oxygen (negative) atoms, as well as between different metal cluster atoms. In all the $Ag_{20}TiO_2$ and $Ag_{37}TiO_2x$ systems, some of Ti atoms in the second layer frozen during the geometry optimization have a slightly lower charge than the other layers while in $Au_{20}TiO_2$ and $Cu_{20}TiO_2$ systems they appear not only in the second layer but also in the surface as shown in figure S1. For the oxygen atoms on the surface of the rutile (110), oxygen atoms have slightly lower charges than the other oxygen atoms in all $M_{20}TiO_2$ but there is no significant change in the oxygen atoms in the $Ag_{37}TiO_2x$ systems. The adsorbed metal nanoparticles on the TiO_2 surface displayed positive and negative charges. Some atoms had a neutral charge (positive and negative ~ 0.04|e|), while other exhibited a slightly higher positive charge ~ 0.2|e|. $Ag_{37}TiO_2z$ showed quite different behavior regarding the Ti atoms with low charges. Here they appear in the second layer that was relaxed during the geometry optimization but O and Ag atoms behaved similarly with previous systems. This difference in Ti atoms behavior could be due to the difference in the number of layers in the slab considered. $Ag_{37}TiO_2z$ contains five layers compared to four layers in the other systems. It is interesting to see a charge variation in our systems which can be due to the interaction between metallic nanoparticles and the TiO_2 surface. Metal nanoparticles tend to transfer a small amount of negative charge to nearby oxygen atoms, which explains the differences observed. In addition, oxygen atoms on the surface of the rutile may be slightly negatively charged due to their interaction with metallic nanoparticles. In summary these results suggest that the presence of metallic nanoparticles can alter the distribution of electronic charges on the surface of TiO_2, in general some cluster atoms assume a positive charge, suggesting a net, although weak, electronic transfer from the metal cluster to the TiO_2 surface.

Table S1 : Ion charges using Bader analysis calculated by VASP. M=Ag, Au, Cu. Indexes 1 and 2 are used to distinguish between different types of the same atomic species, see Fig. S1.

System	Bader charges					
	**Ti1**	**Ti2**	**O1**	**O2**	**M1**	**M2**
**$Ag_{20}TiO_2$**	2.33	2.71	-1.28	-1.31	+/- 0.04	0.19
**$Au_{20}TiO_2$**	2.39	2.71	-1.26	-1.36	+/- 0.04	0.17
**$Ag_{37}TiO_2z$**	2.35	2.68	-1.27	-1.35	+/- 0.04	0.20
**$Cu_{20}TiO_2$**	2.31	2.71	-1.30	-1.35	+/- 0.02	0.32
**$Ag_{37}TiO_2x$**	2.35	2.71	-1.28	-1.35	+/- 0.01	0.28

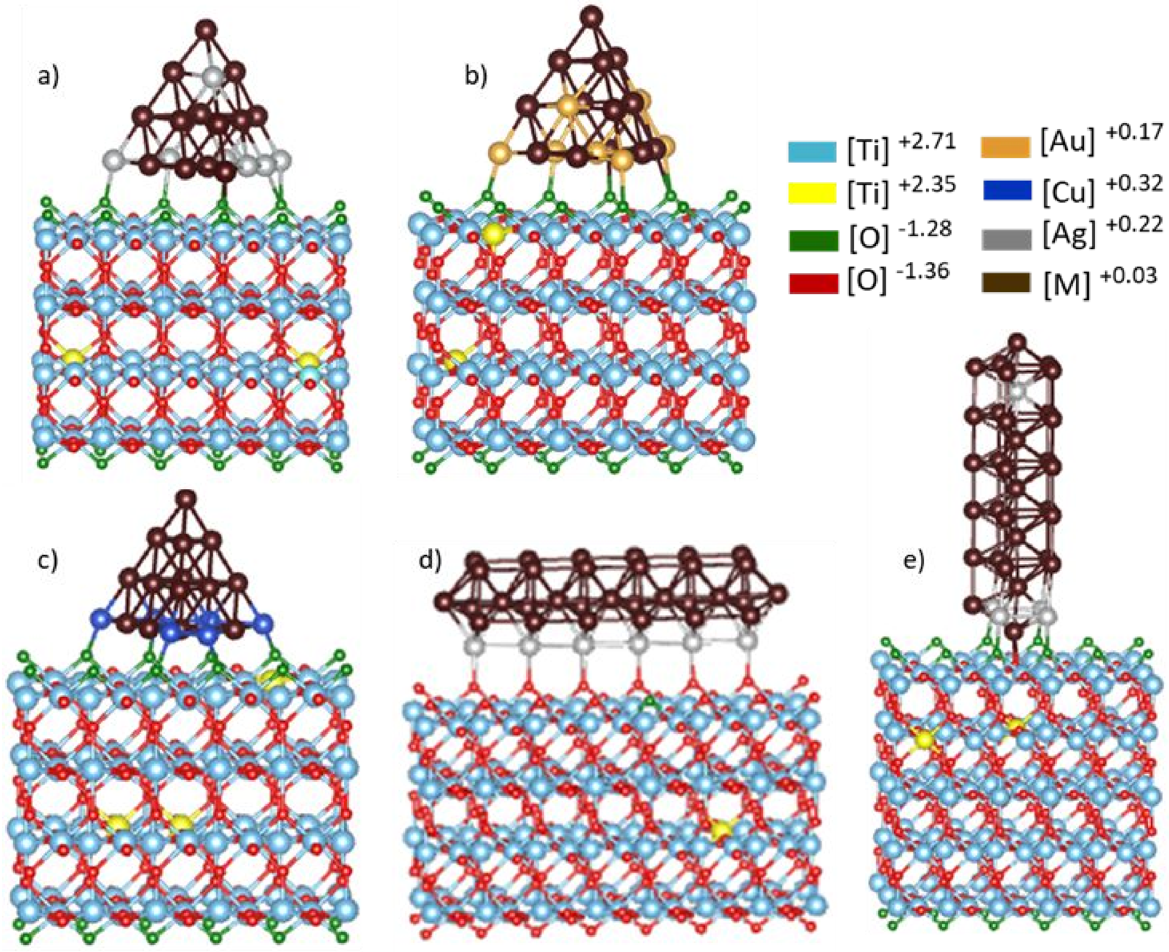

Figure S2.

Figure S2. Ion charges using Bader analysis calculated by VASP. a) b) and c) $M_{20}TiO_2$ respectively for M = Ag, Au, Cu, d) $Ag_{37}TiO_2x$ and e) $Ag_{37}TiO_2z$.

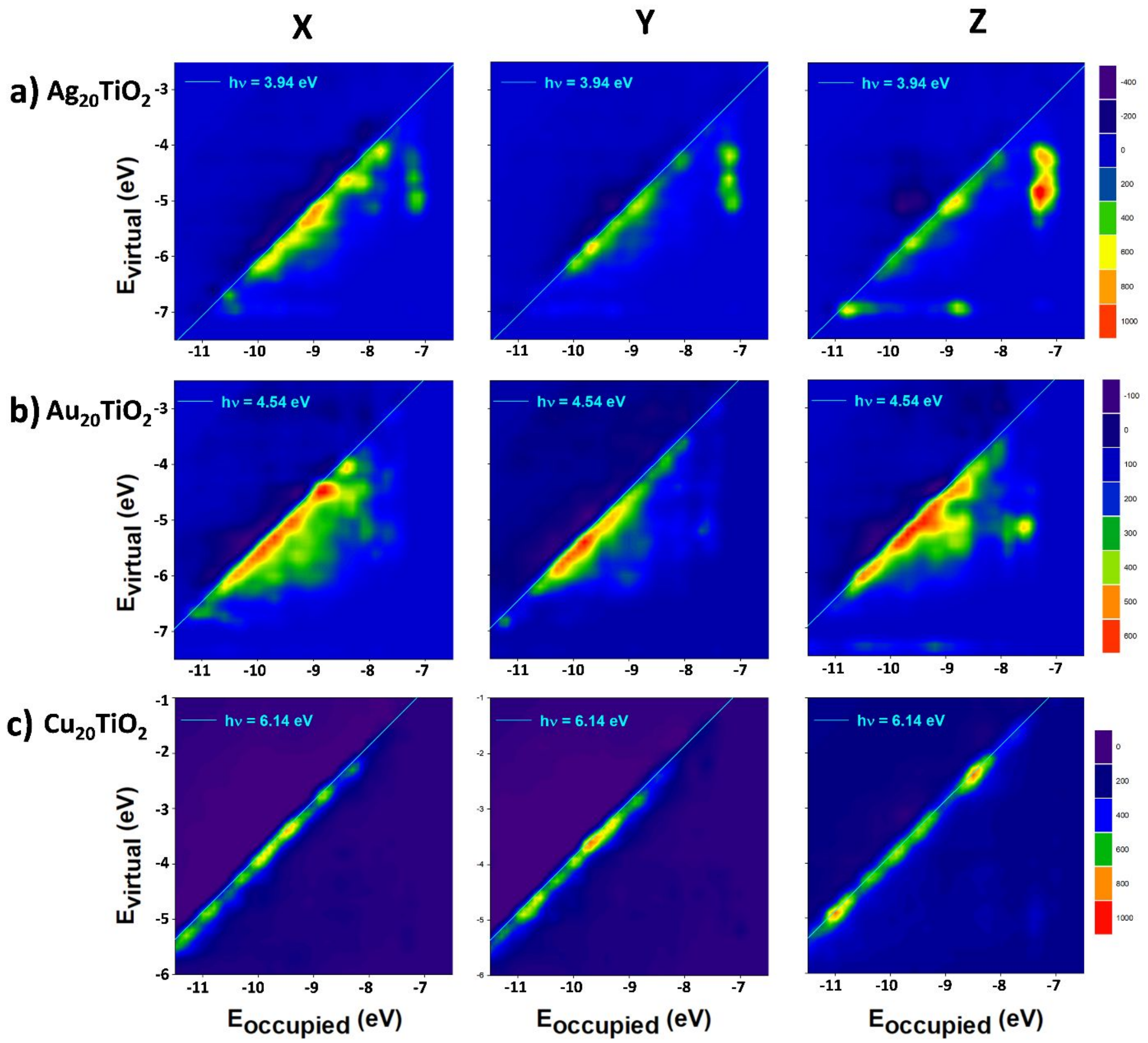

Figure S3. Two-dimensional ICM-OS plots for X, Y and Z components of $[Ti_{54}O_{155}M_{20}H_{137}]^{+1/3}$ at the energy corresponding to the absorption maxima (see values in Figure 4), M = Ag, Au, Cu

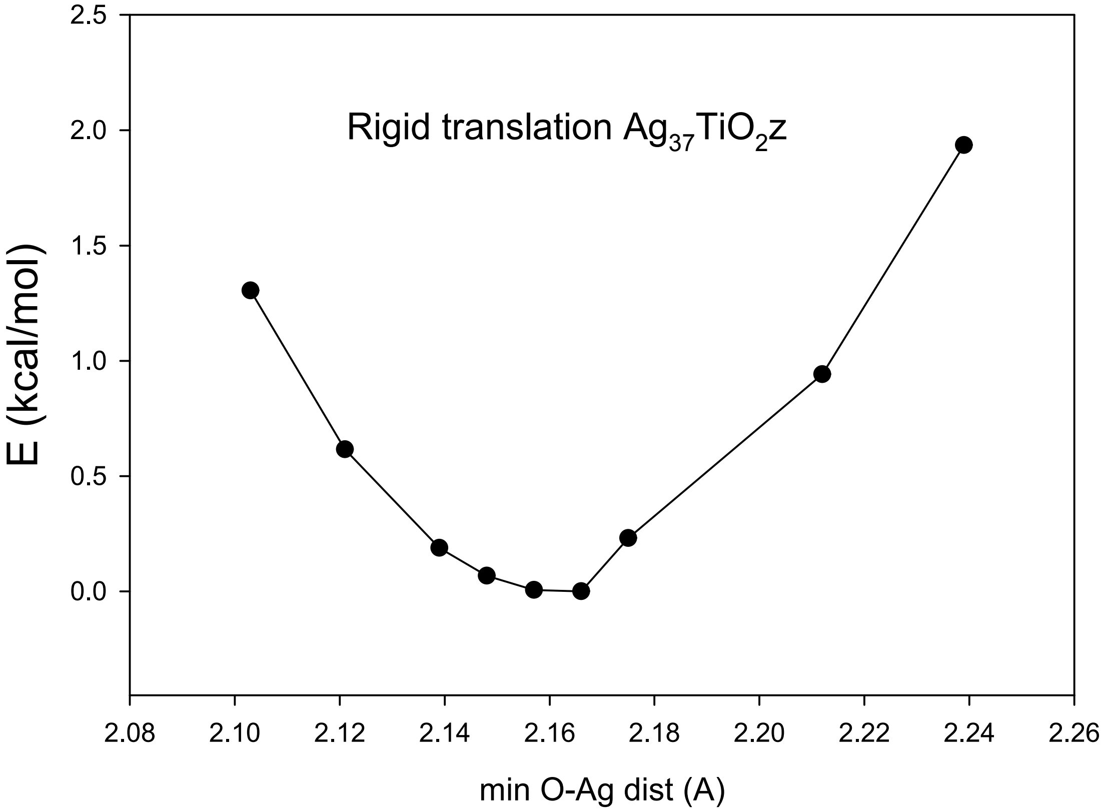

Figure S4. Energy profile calculated including dispersion forces at the PBE-D3BJ level. Energy points are obtained by a rigid translation of the Ag_{37} fragment towards the TiO_2 surface.

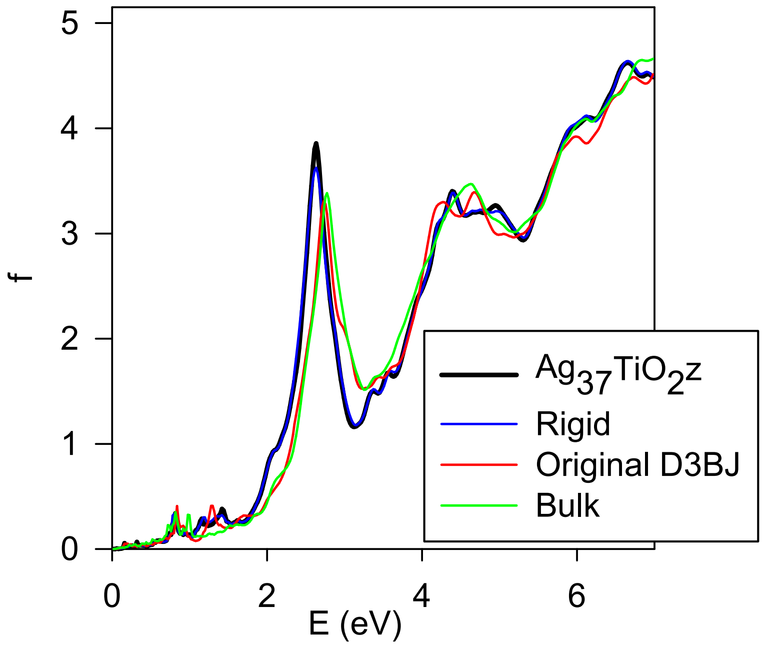

Figure S5. TDDFT photoabsorption spectra of $[Ti_{54}O_{155}Ag_{37}H_{137}]^{+4/3}$ calculated with the complex polarizability algorithm, with different cluster geometries including dispersion forces. Black curve: original geometry from Figure 3 without D3 correction. Blue curve: geometry minimum obtained by rigid translation of the Ag_{37} fragment towards the TiO_2 surface including D3BJ correction (see also Figure S4). Red curve: geometry optimized including D3BJ correction by relaxing the original slab model. Green curve: geometry optimized including D3BJ correction for the slab obtained by D3BJ optimization of the bulk TiO_2, then cutting the cluster.

Table S2. Cartesian coordinate of cluster Ag_{20}-TiO_2

Ti	2.2908217736743168	5.6536768942841782	11.503097411000001
Ti	5.2438602616743175	5.6424982632841783	11.452191973000000
Ti	8.2275007566743170	5.6304802332841781	11.456505352000001
Ti	11.224084933674316	5.6330455892841798	11.501040406000000
Ti	14.215264071674316	5.6448573052841802	11.539325075000001
Ti	2.2938887456743160	12.241852640284179	11.553267732000000
Ti	5.2522871316743149	12.246523567284179	11.531059259999999
Ti	8.2562572686743181	12.245325773284179	11.522520374999999
Ti	11.218657323674318	12.236526226284180	11.565211022000000
Ti	14.182472699674317	12.239060930284179	11.557185207000000
Ti	2.2998447776743163	5.6875193072841803	4.7132988659999997
Ti	5.2698445496743176	5.6875193072841803	4.7132988659999997
Ti	8.2398443226743154	5.6875193072841803	4.7132988659999997
Ti	11.209846749674316	5.6875193072841803	4.7132988659999997
Ti	14.179843866674318	5.6875193072841803	4.7132988659999997
Ti	2.2998447776743163	12.280582905284181	4.7132988659999997
Ti	5.2698445496743176	12.280582905284181	4.7132988659999997
Ti	8.2398443226743154	12.280582905284181	4.7132988659999997
Ti	11.209846749674316	12.280582905284181	4.7132988659999997
Ti	14.179843866674318	12.280582905284181	4.7132988659999997
Ti	2.2961962766743156	2.3801831892841800	7.8169181649999997
Ti	5.2692457606743162	2.3795842922841803	7.8094729789999997
Ti	8.2429033276743162	2.3686956782841797	7.8102066790000002
Ti	11.213173949674317	2.3665783172841799	7.8108799060000003
Ti	14.179007419674317	2.3695786982841796	7.8231997309999999
Ti	2.2976978976743148	8.9533051562841806	7.8217502840000002
Ti	5.2746348676743153	8.9529208252841812	7.8264912219999996
Ti	8.2436574576743169	8.9623239842841791	7.8460785949999998
Ti	11.205120160674316	8.9647808782841807	7.8343535729999996
Ti	14.175526207674316	8.9617816752841790	7.8246831930000003
Ti	3.7825543926743173	2.3540533462841804	11.125862365000000
Ti	6.7546519206743163	2.3446714082841797	11.120785522000000
Ti	9.7305572726743179	2.3428723582841791	11.123242211000001
Ti	12.700102973674316	2.3427179182841797	11.136539515000001
Ti	3.7824402116743165	8.9525812922841794	11.138816676999999
Ti	6.7529684056743164	8.9453733042841783	11.158766884000000
Ti	9.7253925456743175	8.9430555242841798	11.182241496000000
Ti	12.696613795674317	8.9438737032841793	11.148229577000000
Ti	3.7824933186743159	5.6653966542841800	8.0230561349999991
Ti	6.7638621296743153	5.6647706412841785	8.0625559209999995
Ti	9.7292428566743148	5.6651561522841796	8.1068887699999994
Ti	12.692354554674317	5.6647152312841804	8.1300118860000001
Ti	3.7842219756743170	12.258635912284181	8.1315945610000000
Ti	6.7522487956743156	12.257190542284178	8.1379432129999998
Ti	9.7247977386743152	12.258598186284178	8.1365117189999996
Ti	12.695992435674317	12.257765860284181	8.1488887089999995
Ti	3.7848433366743173	2.3909869192841793	4.3119036970000000
Ti	6.7548444356743147	2.3909869192841793	4.3119036970000000
Ti	9.7248455356743158	2.3909869192841793	4.3119036970000000
Ti	12.694845307674317	2.3909869192841793	4.3119036970000000
Ti	3.7848433366743173	8.9840516952841796	4.3119036970000000
Ti	6.7548444356743147	8.9840516952841796	4.3119036970000000
Ti	9.7248455356743158	8.9840516952841796	4.3119036970000000
Ti	12.694845307674317	8.9840516952841796	4.3119036970000000
O	2.3031918926743167	3.5904084642841809	11.541611686000000
O	5.2739033086743170	3.5903742752841801	11.517063690000001
O	8.2417030926743173	3.5847613812841814	11.498681983999999
O	11.212451684674317	3.5872937262841802	11.506070005000000
O	14.177467294674315	3.5889383362841798	11.516799123000000
O	2.2985263786743175	10.185798093284181	11.514773300000000
O	5.2712651816743161	10.187116139284178	11.530801307999999
O	8.2357882186743154	10.185093092284180	11.526486983000000
O	11.214846843674316	10.178533516284180	11.525060214000000
O	14.176814069674318	10.181777936284181	11.517589043999999
O	2.2724292356743163	4.3556919422841780	8.0339061980000004
O	5.2948914866743166	4.3542501092841803	8.0274573890000003

O	8.2660981326743155	4.3554160722841786	8.0360208409999991
O	11.210492008674315	4.3622456242841814	8.0099714300000002
O	14.153667062674316	4.3562684402841789	8.0466412980000008
O	2.2993840686743177	10.963388440284181	7.9914980709999996
O	5.2716926996743148	10.964341017284180	7.9916038970000001
O	8.2436415256743167	10.966008026284179	7.9986111309999997
O	11.208981093674318	10.964824379284181	7.9949119240000002
O	14.176333444674317	10.963659594284181	7.9948542859999998
O	2.2998447776743163	3.6882562592841808	4.5247139409999999
O	5.2698445496743176	3.6882562592841808	4.5247139409999999
O	8.2398443226743154	3.6882562592841808	4.5247139409999999
O	11.209846749674316	3.6882562592841808	4.5247139409999999
O	14.179843866674318	3.6882562592841808	4.5247139409999999
O	2.2998447776743163	10.281322214284181	4.5247139409999999
O	5.2698445496743176	10.281322214284181	4.5247139409999999
O	8.2398443226743154	10.281322214284181	4.5247139409999999
O	11.209846749674316	10.281322214284181	4.5247139409999999
O	14.179843866674318	10.281322214284181	4.5247139409999999
O	2.3030670896743182	1.1118719492841791	11.486361687000000
O	2.3030670896743182	14.297999143284180	11.486361687000000
O	5.2673989386743152	1.1118247922841800	11.478069416000000
O	5.2673989386743152	14.297954344284179	11.478069416000000
O	8.2441194946743153	1.1098713022841800	11.486950348000001
O	8.2441194946743153	14.295999675284179	11.486950348000001
O	11.209055444674316	1.1087536742841788	11.506375201000001
O	11.209055444674316	14.294882048284180	11.506375201000001
O	14.183996891674315	1.1101884342841792	11.496110019000000
O	14.183996891674315	14.296315629284180	11.496110019000000
O	2.3013981796743153	7.7050192502841810	11.494588761999999
O	5.2669873536743168	7.6895563822841808	11.457926453000001
O	8.2358187556743161	7.6790709632841789	11.468571476999999
O	11.211933883674316	7.6848854542841813	11.525350292000001
O	14.178447132674318	7.6986459452841807	11.511735508999999
O	2.2745362856743156	6.9775181752841782	8.0624236370000002
O	5.2951450766743164	6.9759749542841796	8.0642009580000007
O	8.2642606046743161	6.9732645902841810	8.0712374830000009
O	11.209002336674317	6.9674854662841810	8.0372057209999994
O	14.153035080674318	6.9756872942841781	8.0660803249999997
O	2.2992712146743166	0.37191546028418010	8.0122570960000008
O	2.2992712146743166	13.558042655284179	8.0122570960000008
O	5.2718626436743179	0.37200859628418037	8.0109200900000008
O	5.2718626436743179	13.558136969284181	8.0109200900000008
O	8.2415809456743148	0.37025080928417964	8.0093572569999996
O	8.2415809456743148	13.556378003284181	8.0093572569999996
O	11.210048558674316	0.36999733828417902	8.0051591529999993
O	11.210048558674316	13.556123354284178	8.0051591529999993
O	14.177894812674317	0.36996432828417980	8.0127134729999998
O	14.177894812674317	13.556092702284179	8.0127134729999998
O	2.2998447776743163	1.0937164002841797	4.5247139409999999
O	2.2998447776743163	14.279845953284180	4.5247139409999999
O	5.2698445496743176	1.0937164002841797	4.5247139409999999
O	5.2698445496743176	14.279845953284180	4.5247139409999999
O	8.2398443226743154	1.0937164002841797	4.5247139409999999
O	8.2398443226743154	14.279845953284180	4.5247139409999999
O	11.209846749674316	1.0937164002841797	4.5247139409999999
O	11.209846749674316	14.279845953284180	4.5247139409999999
O	14.179843866674318	1.0937164002841797	4.5247139409999999
O	14.179843866674318	14.279845953284180	4.5247139409999999
O	2.2998447776743163	7.6867811772841783	4.5247139409999999
O	5.2698445496743176	7.6867811772841783	4.5247139409999999
O	8.2398443226743154	7.6867811772841783	4.5247139409999999
O	11.209846749674316	7.6867811772841783	4.5247139409999999
O	14.179843866674318	7.6867811772841783	4.5247139409999999
O	0.82567090467431647	5.6449975982841814	12.738059093000000
O	15.675671092674317	5.6449975982841814	12.738059093000000
O	3.7963411616743166	5.6685927382841790	12.754448990000000
O	6.7597290206743175	5.6254108272841812	12.729137530999999
O	9.7072509426743174	5.5897846892841798	12.729271704000000

O	12.689290238674317	5.6174589322841797	12.760744729000001
O	0.81818005767431679	12.249923606284181	12.733003037000000
O	15.668177590674315	12.249923606284181	12.733003037000000
O	3.7977551546743165	12.251161485284179	12.729371861000001
O	6.7591859936743148	12.300346516284179	12.753777180000000
O	9.7069561946743157	12.229117820284181	12.739136257000000
O	12.694494796674316	12.247950075284180	12.738316100000000
O	0.80418616867431680	5.6431832222841791	10.113624141000001
O	15.654186357674316	5.6431832222841791	10.113624141000001
O	3.7769249726743155	5.6271521092841787	10.114887445999999
O	6.7580415216743148	5.6261806692841780	10.096545426000000
O	9.7420524426743178	5.6382034152841811	10.103084944000001
O	12.690933923674315	5.6467577432841800	10.120139092000001
O	0.81452226267431627	12.238829859284181	10.099460382000000
O	15.664521122674316	12.238829859284181	10.099460382000000
O	3.7871070536743154	12.238261614284180	10.096546370000000
O	6.7561880616743153	12.237155776284180	10.103038645000000
O	9.7289534196743155	12.245620506284180	10.100396759000001
O	12.690763978674315	12.241885650284178	10.103584785000001
O	3.7848433366743173	2.3909869192841793	2.4691270660000000
O	6.7548444356743147	2.3909869192841793	2.4691270660000000
O	9.7248455356743158	2.3909869192841793	2.4691270660000000
O	12.694845307674317	2.3909869192841793	2.4691270660000000
O	3.7848433366743173	8.9840516952841796	2.4691270660000000
O	6.7548444356743147	8.9840516952841796	2.4691270660000000
O	9.7248455356743158	8.9840516952841796	2.4691270660000000
O	12.694845307674317	8.9840516952841796	2.4691270660000000
O	0.81484489167431562	5.6875193072841803	3.2807229809999998
O	15.664845080674315	5.6875193072841803	3.2807229809999998
O	3.7848433366743173	5.6875193072841803	3.2807229809999998
O	6.7548444356743147	5.6875193072841803	3.2807229809999998
O	9.7248455356743158	5.6875193072841803	3.2807229809999998
O	12.694845307674317	5.6875193072841803	3.2807229809999998
O	0.81484489167431562	12.280582905284181	3.2807229809999998
O	15.664845080674315	12.280582905284181	3.2807229809999998
O	3.7848433366743173	12.280582905284181	3.2807229809999998
O	6.7548444356743147	12.280582905284181	3.2807229809999998
O	9.7248455356743158	12.280582905284181	3.2807229809999998
O	12.694845307674317	12.280582905284181	3.2807229809999998
O	0.81098130367431587	2.3585450762841802	9.2476675959999994
O	15.660978836674317	2.3585450762841802	9.2476675959999994
O	3.7850358516743157	2.3681981692841791	9.2374430440000008
O	6.7608588876743170	2.3558052392841802	9.2342578510000006
O	9.7320044586743180	2.3542325442841801	9.2338515529999992
O	12.687683729674315	2.3537597922841798	9.2429375240000002
O	0.80753593967431669	8.9846435192841803	9.2502490089999991
O	15.657534799674316	8.9846435192841803	9.2502490089999991
O	3.7828717116743178	8.9843487862841798	9.2480616110000007
O	6.7577653616743163	8.9929078302841781	9.2623415900000001
O	9.7343571316743152	8.9817126952841804	9.2743236200000005
O	12.689614195674316	8.9775015512841811	9.2549044360000003
O	0.68385598467431663	5.6853889782841804	6.0591044820000004
O	15.533854845674316	5.6853889782841804	6.0591044820000004
O	3.7852150906743169	5.6866692972841797	6.1277552489999998
O	6.8837183276743161	5.6871585542841814	6.0554714159999996
O	9.7459611726743169	5.6864193642841805	5.9794685339999996
O	12.670293600674317	5.6854043042841802	5.9789389279999998
O	0.81221473167431668	12.278142516284181	5.9642157999999998
O	15.662213591674316	12.278142516284181	5.9642157999999998
O	3.7855908276743158	12.278571647284181	5.9654186329999996
O	6.7582685576743167	12.277463451284181	5.9652707590000000
O	9.7257696106743161	12.277329053284181	5.9642016269999996
O	12.692513878674315	12.277553050284180	5.9629884000000004
O	0.80487656967431676	2.3695008892841791	6.5848256129999996
O	15.654876757674316	2.3695008892841791	6.5848256129999996
O	3.7822397296743162	2.3700373032841799	6.5842879759999997
O	6.7633921266743151	2.3690257792841791	6.5774215290000004
O	9.7291021206743160	2.3702082482841789	6.5726210639999998

O	12.695761416674316	2.3707069362841793	6.5775179069999998		
O	0.80495755867431562	8.9640782342841803	6.5886849769999998		
O	15.654956419674317	8.9640782342841803	6.5886849769999998		
O	3.7867220226743150	8.9617203712841800	6.5942200870000001		
O	6.7686179276743168	8.9627342522841786	6.5992024430000003		
O	9.7246145176743148	8.9625291182841806	6.5965100049999998		
O	12.687359772674316	8.9643894722841786	6.5885144259999997		
Ag	7.6621343696743160	6.7415007622841792	21.663115185999999		
Ag	7.5139345726743159	7.2128376602841797	14.725851498999999		
Ag	6.9926834556743174	11.837580451284179	14.955029436000000		
Ag	7.2794360296743150	10.306044412284180	17.254567218999998		
Ag	7.4819929236743157	8.5113849622841791	19.449891294000000		
Ag	5.7712971026743176	9.4048147812841805	15.028298307000000		
Ag	6.0038811116743176	7.6665860762841795	17.203375481999998		
Ag	6.2538114346743150	5.9247816722841797	19.443500122000000		
Ag	4.6469731056743164	6.9100113662841807	14.877226084000000		
Ag	4.9782339456743152	5.0076140632841799	17.129584092000002		
Ag	3.6735441226743148	4.3198264822841814	14.835954646999999		
Ag	8.7574164006743160	9.7344983182841780	14.898096604999999		
Ag	8.9475235136743159	7.9859657512841800	17.148426900000000		
Ag	9.1380528326743153	6.2175752572841780	19.439565640000001		
Ag	7.8071397366743156	5.2192983262841786	17.194964155000001		
Ag	6.4550114266743179	4.5318891812841784	14.827975131000001		
Ag	10.409159977674317	7.5579545932841796	14.852546749000000		
Ag	10.679956331674315	5.6891061492841786	17.164603253999999		
Ag	9.3393364686743148	4.9105938782841783	14.891262284000000		
Ag	12.182881406674316	5.4419808422841811	14.934436710000000		
H	1.5427014332470872	2.9576218691707972	11.336322752628101	nuclear_charge=	0.66666666666666663
H	14.940268423876525	2.9551124061323133	11.323648177998550	nuclear_charge=	0.66666666666666663
H	1.5358801568594469	9.5511153656990508	11.323836829789085	nuclear_charge=	0.66666666666666663
H	14.940566428751843	9.5488894324527465	11.325124409010806	nuclear_charge=	0.66666666666666663
H	1.5185794398347880	5.0280320974800148	8.0579345359354324	nuclear_charge=	0.66666666666666663
H	14.914290720075872	5.0211445034322537	8.0639511926789638	nuclear_charge=	0.66666666666666663
H	1.5407374704931129	11.626165887624513	8.0695336677819753	nuclear_charge=	0.66666666666666663
H	14.937505133077448	11.623770153122980	8.0708797480706522	nuclear_charge=	0.66666666666666663
H	1.5433001288180801	3.0273523854489603	4.4162961203403226	nuclear_charge=	0.66666666666666663
H	14.936388812785083	3.0273527168030014	4.4162961746972185	nuclear_charge=	0.66666666666666663
H	1.5433004229946494	9.6204179967859353	4.4162961624977584	nuclear_charge=	0.66666666666666663
H	14.936388518608695	9.6204183281398912	4.4162962168545903	nuclear_charge=	0.66666666666666663
H	2.2985592444683540	0.10201649057496631	11.519221873347682	nuclear_charge=	0.66666666666666663
H	1.5392247179509457	1.7489162355320538	11.308520273040960	nuclear_charge=	0.66666666666666663
H	1.5392252952969336	14.935044159318693	11.308520407461399	nuclear_charge=	0.66666666666666663
H	3.0637549439499239	14.936674649555620	11.301008656146722	nuclear_charge=	0.66666666666666663
H	5.2599585391503076	0.10178909127736269	11.504159321070572	nuclear_charge=	0.66666666666666663
H	4.5049512203231821	14.935820801166905	11.297215828905280	nuclear_charge=	0.66666666666666663
H	6.0323146192729915	14.932025157901425	11.294313151091764	nuclear_charge=	0.66666666666666663
H	8.2500989634512152	9.9640956556616089E-002	11.504473319332300	nuclear_charge=	0.66666666666666663
H	7.4798292555270649	14.929613045515215	11.299060254038913	nuclear_charge=	0.66666666666666663
H	9.0083599531551855	14.929937002507824	11.299952629368214	nuclear_charge=	0.66666666666666663
H	11.213766812890519	9.8777169405039444E-002	11.535244260590682	nuclear_charge=	0.66666666666666663
H	10.448278694168536	14.929910161931520	11.309230100205271	nuclear_charge=	0.66666666666666663
H	11.973629655338680	14.927629978510854	11.316732133157217	nuclear_charge=	0.66666666666666663
H	14.183248630374621	0.10023367984520704	11.526093249165234	nuclear_charge=	0.66666666666666663
H	14.945403660630291	1.7486816493769091	11.313095521026286	nuclear_charge=	0.66666666666666663
H	14.945403083532518	14.934809572328668	11.313095659739604	nuclear_charge=	0.66666666666666663
H	13.419905509470329	14.930974243674747	11.310958829909984	nuclear_charge=	0.66666666666666663
H	1.5388651139706226	8.3429698422247398	11.314392963072233	nuclear_charge=	0.66666666666666663
H	14.937559749484272	8.3382699372456450	11.323221997793977	nuclear_charge=	0.66666666666666663
H	1.5200527232845662	6.3055252994263107	8.0718198532569136	nuclear_charge=	0.66666666666666663
H	14.914179064708460	6.3112232067161038	8.0735282710899590	nuclear_charge=	0.66666666666666663
H	1.5417378572250939	-0.29330691257643871	8.0795665826820215	nuclear_charge=	0.66666666666666663
H	3.0582688882838305	-0.29224575025006949	8.0732536358774976	nuclear_charge=	0.66666666666666663
H	2.2977314199506189	14.563695469385888	7.9144398862589531	nuclear_charge=	0.66666666666666663
H	1.5417378572250939	12.892820282423560	8.0795665826820215	nuclear_charge=	0.66666666666666663
H	4.5123214184286482	-0.29147402524782251	8.0725325731290329	nuclear_charge=	0.66666666666666663
H	6.0292675293528326	-0.29359076251985705	8.0759084944270256	nuclear_charge=	0.66666666666666663
H	5.2705521645095637	14.563487447780393	7.9100396754895739	nuclear_charge=	0.66666666666666663
H	7.4817774650165561	-0.29254892096479601	8.0749571682974928	nuclear_charge=	0.66666666666666663

H	9.0004149780710172	-0.29371233196095403	8.0744112218634445	nuclear_charge=	0.66666666666666663
H	8.2422462375477004	14.561797867903675	7.9091643769373867	nuclear_charge=	0.66666666666666663
H	10.450802906532378	-0.29328660929940931	8.0723052964595841	nuclear_charge=	0.66666666666666663
H	11.968908656733255	-0.29306504186091509	8.0785607307098743	nuclear_charge=	0.66666666666666663
H	11.211622772088834	14.561772380696677	7.9073035577194055	nuclear_charge=	0.66666666666666663
H	14.937974166830983	-0.29239028376468745	8.0796007160466292	nuclear_charge=	0.66666666666666663
H	13.419722761436883	-0.29428666388329283	8.0823835535722921	nuclear_charge=	0.66666666666666663
H	14.178454502007362	14.561985019242215	7.9173798827359096	nuclear_charge=	0.66666666666666663
H	14.937973870300635	12.893737747319452	8.0796006899518567	nuclear_charge=	0.66666666666666663
H	2.2998447776743163	8.7781709410732489E-002	4.6196010196637642	nuclear_charge=	0.66666666666666663
H	1.5433004229946494	1.7546206177824235	4.4162961624977584	nuclear_charge=	0.66666666666666663
H	1.5432998346414344	14.940749483455974	4.4162960781828753	nuclear_charge=	0.66666666666666663
H	3.0563894236762366	14.940749814560792	4.4162960238668143	nuclear_charge=	0.66666666666666663
H	5.2698445496743176	8.7781709410732489E-002	4.6196010196637642	nuclear_charge=	0.66666666666666663
H	4.5132993096109217	14.940749152351362	4.4162961324989034	nuclear_charge=	0.66666666666666663
H	6.0263894927071995	14.940749483455978	4.4162960781828753	nuclear_charge=	0.66666666666666663
H	8.2398443226743154	8.7781709410732489E-002	4.6196010196637642	nuclear_charge=	0.66666666666666663
H	7.4832993794175984	14.940749483206464	4.4162960782238070	nuclear_charge=	0.66666666666666663
H	8.9963895627377113	14.940749152351362	4.4162961324989034	nuclear_charge=	0.66666666666666663
H	11.209846749674316	8.7781709410732489E-002	4.6196010196637642	nuclear_charge=	0.66666666666666663
H	10.453301509387082	14.940749152101848	4.4162961325398351	nuclear_charge=	0.66666666666666663
H	11.966391395452398	14.940749814810303	4.4162960238258826	nuclear_charge=	0.66666666666666663
H	14.179843866674318	8.7781709410732489E-002	4.6196010196637642	nuclear_charge=	0.66666666666666663
H	14.936388518608695	1.7546202864284677	4.4162962168545903	nuclear_charge=	0.66666666666666663
H	14.936389106961553	14.940749152101851	4.4162961325398342	nuclear_charge=	0.66666666666666663
H	13.423299220672398	14.940749814560792	4.4162960238668143	nuclear_charge=	0.66666666666666663
H	1.5433004227451370	8.3476853944909344	4.4162961624620012	nuclear_charge=	0.66666666666666663
H	14.936388518858209	8.3476850631369786	4.4162962168188340	nuclear_charge=	0.66666666666666663
H	4.4676516601695582E-002	5.6449225724569274	12.097000655593396	nuclear_charge=	0.66666666666666663
H	16.448230187696261	5.6495741066033318	12.086875815980020	nuclear_charge=	0.66666666666666663
H	2.5898133001916790E-002	12.244130871665242	12.105975161971280	nuclear_charge=	0.66666666666666663
H	16.457377422140375	12.245607307728079	12.102088497953138	nuclear_charge=	0.66666666666666663
H	8.6436508657943278E-002	5.6440182741376930	10.824780163427414	nuclear_charge=	0.66666666666666663
H	0.80342072959534683	5.6547992276350989	9.1032912045267285	nuclear_charge=	0.66666666666666663
H	16.392352135399474	5.6483936964930166	10.803546089885476	nuclear_charge=	0.66666666666666663
H	15.653420918098348	5.6547992276350953	9.1032912045271051	nuclear_charge=	0.66666666666666663
H	9.4174804841458126E-002	12.238942171004048	10.807985390295274	nuclear_charge=	0.66666666666666663
H	0.81640655775640347	12.248436418201603	9.0891078081636145	nuclear_charge=	0.66666666666666663
H	16.385179367591515	12.240302376118422	10.807667756804182	nuclear_charge=	0.66666666666666663
H	15.666406103858893	12.248436418189439	9.0891078094433038	nuclear_charge=	0.66666666666666663
H	3.0720793337668866	2.3909869192841793	1.7529737052179830	nuclear_charge=	0.66666666666666663
H	4.4976079795308834	2.3909869192841793	1.7529743421389861	nuclear_charge=	0.66666666666666663
H	6.0420801127921031	2.3909869192841793	1.7529740236785538	nuclear_charge=	0.66666666666666663
H	7.4676087587976525	2.3909869192841793	1.7529740239185398	nuclear_charge=	0.66666666666666663
H	9.0120808928177496	2.3909869192841793	1.7529743421389861	nuclear_charge=	0.66666666666666663
H	10.437610178772008	2.3909869192841793	1.7529743423789714	nuclear_charge=	0.66666666666666663
H	11.982081305008013	2.3909869192841793	1.7529737049779972	nuclear_charge=	0.66666666666666663
H	13.407609310581748	2.3909869192841793	1.7529737052179830	nuclear_charge=	0.66666666666666663
H	3.0720793337668866	8.9840516952841796	1.7529737052179830	nuclear_charge=	0.66666666666666663
H	4.4976079795308834	8.9840516952841796	1.7529743421389861	nuclear_charge=	0.66666666666666663
H	6.0420801127921031	8.9840516952841796	1.7529740236785538	nuclear_charge=	0.66666666666666663
H	7.4676087587976525	8.9840516952841796	1.7529740239185398	nuclear_charge=	0.66666666666666663
H	9.0120808928177496	8.9840516952841796	1.7529743421389861	nuclear_charge=	0.66666666666666663
H	10.437610178772008	8.9840516952841796	1.7529743423789714	nuclear_charge=	0.66666666666666663
H	11.982081305008013	8.9840516952841796	1.7529737049779972	nuclear_charge=	0.66666666666666663
H	13.407609310581748	8.9840516952841796	1.7529737052179830	nuclear_charge=	0.66666666666666663
H	8.7663592089814557E-002	5.6875193072841803	3.9822324374826583	nuclear_charge=	0.66666666666666663
H	0.81484489167431562	5.6875193072841803	2.2703229809999996	nuclear_charge=	0.66666666666666663
H	16.392025753556347	5.6875193072841803	3.9822330871188178	nuclear_charge=	0.66666666666666663
H	15.664845080674315	5.6875193072841803	2.2703229809999996	nuclear_charge=	0.66666666666666663
H	3.7848433366743173	5.6875193072841803	2.2703229809999996	nuclear_charge=	0.66666666666666663
H	6.7548444356743147	5.6875193072841803	2.2703229809999996	nuclear_charge=	0.66666666666666663
H	9.7248455356743158	5.6875193072841803	2.2703229809999996	nuclear_charge=	0.66666666666666663
H	12.694845307674317	5.6875193072841803	2.2703229809999996	nuclear_charge=	0.66666666666666663
H	8.7663592089814557E-002	12.280582905284181	3.9822324374826583	nuclear_charge=	0.66666666666666663
H	0.81484489167431562	12.280582905284181	2.2703229809999996	nuclear_charge=	0.66666666666666663
H	16.392025753556347	12.280582905284181	3.9822330871188178	nuclear_charge=	0.66666666666666663
H	15.664845080674315	12.280582905284181	2.2703229809999996	nuclear_charge=	0.66666666666666663

H	3.7848433366743173	12.280582905284181	2.2703229809999996	nuclear_charge= 0.66666666666666663
H	6.7548444356743147	12.280582905284181	2.2703229809999996	nuclear_charge= 0.66666666666666663
H	9.7248455356743158	12.280582905284181	2.2703229809999996	nuclear_charge= 0.66666666666666663
H	12.694845307674317	12.280582905284181	2.2703229809999996	nuclear_charge= 0.66666666666666663
H	8.2537206232835558E-002	2.3639684974956925	8.5474905159659329	nuclear_charge= 0.66666666666666663
H	0.81338377636524406	2.3553272055837020	10.258059615680047	nuclear_charge= 0.66666666666666663
H	16.388617843727552	2.3691460473735937	8.5467130647880651	nuclear_charge= 0.66666666666666663
H	15.663383436019728	2.3553272055998136	10.258059610621180	nuclear_charge= 0.66666666666666663
H	7.9387783398120249E-002	8.9734109294601900	8.5498332385911500	nuclear_charge= 0.66666666666666663
H	0.81068162866475113	8.9660907861078449	10.260473766532927	nuclear_charge= 0.66666666666666663
H	16.386843576239460	8.9693060435902012	8.5511198147416074	nuclear_charge= 0.66666666666666663
H	15.660681197469643	8.9660907861483707	10.260473764326310	nuclear_charge= 0.66666666666666663
H	-3.2772710382737102E-002	5.6865164827274910	5.3468192847969735	nuclear_charge= 0.66666666666666663
H	0.74312125770208937	5.6759928334547176	7.0677211088088630	nuclear_charge= 0.66666666666666663
H	16.310266441545838	5.6864125077453913	5.4125040287186277	nuclear_charge= 0.66666666666666663
H	15.593120778474795	5.6759928338158403	7.0677210700444464	nuclear_charge= 0.66666666666666663
H	4.0017941296117243E-002	12.279413763823225	5.3125880988858585	nuclear_charge= 0.66666666666666663
H	0.81497497334541613	12.268538649420279	6.9745663860874121	nuclear_charge= 0.66666666666666663
H	16.435546278910827	12.279411133160451	5.3139365480914247	nuclear_charge= 0.66666666666666663
H	15.664974448921431	12.268538649436266	6.9745663844056445	nuclear_charge= 0.66666666666666663
H	3.0857927505680394E-002	2.3695416961230951	7.2342894687381172	nuclear_charge= 0.66666666666666663
H	0.80930762629886210	2.3790517259813377	5.5744804703603297	nuclear_charge= 0.66666666666666663
H	16.433810295675443	2.3750803831401921	7.2283623816662157	nuclear_charge= 0.66666666666666663
H	15.659307814743368	2.3790517259813200	5.5744804703622792	nuclear_charge= 0.66666666666666663
H	2.9556954366142918E-002	8.9628745603132778	7.2364971519454020	nuclear_charge= 0.66666666666666663
H	0.80934519255169768	8.9729417199311357	5.5783333811230280	nuclear_charge= 0.66666666666666663
H	16.433941824048972	8.9584563167660107	7.2321585924767131	nuclear_charge= 0.66666666666666663
H	15.659344642858024	8.9729417199086861	5.5783333836821711	nuclear_charge= 0.66666666666666663
H	3.1576071675545698	8.2061162673558208	6.3614431611798476	nuclear_charge= 0.66666666666666663
H	6.0810277182325194	8.2552350390937939	6.3810907119657223	nuclear_charge= 0.66666666666666663
H	8.9894152171045718	8.3001645655729916	6.3923939895738036	nuclear_charge= 0.66666666666666663

Table S3. Cartesian coordinate of cluster Au_{20}-TiO_2.

Ti	2.2716366026743167	5.6514935122841798	11.518376129000000
Ti	5.2308395726743164	5.6399894962841799	11.465591323000000
Ti	8.2172522906743168	5.6387539762841783	11.452915751000001
Ti	11.224018549674316	5.6588960242841786	11.471519504000000
Ti	14.215824358674315	5.6721035962841810	11.519197230000000
Ti	2.2823895926743170	12.241383425284180	11.554433715000000
Ti	5.2369323576743163	12.248862568284181	11.528235012000000
Ti	8.2451843056743179	12.250197119284181	11.535218624000001
Ti	11.202894946674316	12.223242024284179	11.576314312999999
Ti	14.178779056674315	12.219103974284181	11.561365358000000
Ti	2.2998461056743160	5.6875193072841803	4.7132988659999997
Ti	5.2698445496743176	5.6875193072841803	4.7132988659999997
Ti	8.2398443226743154	5.6875193072841803	4.7132988659999997
Ti	11.209846749674316	5.6875193072841803	4.7132988659999997
Ti	14.179843866674318	5.6875193072841803	4.7132988659999997
Ti	2.2998461056743160	12.280582905284181	4.7132988659999997
Ti	5.2698445496743176	12.280582905284181	4.7132988659999997
Ti	8.2398443226743154	12.280582905284181	4.7132988659999997
Ti	11.209846749674316	12.280582905284181	4.7132988659999997
Ti	14.179843866674318	12.280582905284181	4.7132988659999997
Ti	2.2949362956743151	2.3692827862841792	7.8009175580000001
Ti	5.2651431876743153	2.3667268622841799	7.7894438739999998
Ti	8.2382949036743156	2.3656328142841794	7.7935781989999997
Ti	11.208882844674317	2.3668023142841790	7.8080443199999996
Ti	14.176081183674317	2.3714260842841792	7.8036425940000003
Ti	2.2867842576743165	8.9640027832841795	7.8115087240000003
Ti	5.2684664046743173	8.9661484382841792	7.8104244740000004
Ti	8.2500330416743175	8.9679333412841800	7.8215424100000002
Ti	11.219716822674318	8.9663441412841784	7.8286105890000002
Ti	14.163388433674317	8.9616661402841800	7.8108737640000001
Ti	3.7703223536743167	2.3502430392841802	11.102589957999999
Ti	6.7450739396743167	2.3455391022841798	11.097713429000001
Ti	9.7256209086743155	2.3517579662841790	11.111902700000000
Ti	12.692906875674318	2.3489969082841800	11.121449773000000

Ti	3.7784504926743168	8.9527180482841793	11.130984562000000
Ti	6.7441777466743176	8.9436780002841800	11.139770061000000
Ti	9.7163217436743174	8.9498579602841808	11.165183568000000
Ti	12.678283659674317	8.9419190342841794	11.168974429000000
Ti	3.7832912626743180	5.6648979662841796	8.1098632540000004
Ti	6.7514282806743147	5.6653577492841798	8.0954491019999999
Ti	9.7208252806743154	5.6644405402841791	8.1048610560000007
Ti	12.684861053674318	5.6692576552841807	8.0780500719999999
Ti	3.7795710656743147	12.258178486284180	8.1347986510000005
Ti	6.7504696866743181	12.258411915284178	8.1467107599999995
Ti	9.7213298036743154	12.261639830284178	8.1468241460000002
Ti	12.691847376674318	12.253427391284180	8.1589743640000005
Ti	3.7848446636743169	2.3909869192841793	4.3119036970000000
Ti	6.7548431086743150	2.3909869192841793	4.3119036970000000
Ti	9.7248455356743158	2.3909869192841793	4.3119036970000000
Ti	12.694845307674317	2.3909869192841793	4.3119036970000000
Ti	3.7848446636743169	8.9840516952841796	4.3119036970000000
Ti	6.7548431086743150	8.9840516952841796	4.3119036970000000
Ti	9.7248455356743158	8.9840516952841796	4.3119036970000000
Ti	12.694845307674317	8.9840516952841796	4.3119036970000000
O	2.2960117266743154	3.5922829652841806	11.478850831999999
O	5.2610366316743153	3.5926213182841806	11.449650244000001
O	8.2250564726743178	3.5934642552841787	11.436329318000000
O	11.207709163674316	3.6021671292841795	11.457954798999999
O	14.169163900674317	3.5984747142841798	11.480065948000000
O	2.3154491586743156	10.191454608284179	11.501409854000000
O	5.2681251866743182	10.193032018284178	11.507477876999999
O	8.1992846156743155	10.195137590284180	11.508111892000001
O	11.105131567674317	10.211345543284178	11.560304257000000
O	14.263082278674318	10.211649708284181	11.530296742000001
O	2.3175960396743172	4.3576949472841804	8.0120208759999993
O	5.2728278776743167	4.3645138882841792	7.9763346280000000
O	8.2326853996743168	4.3641248412841804	7.9830281610000000
O	11.189695018674318	4.3588939212841780	8.0324633660000000
O	14.172512343674317	4.3661596772841804	7.9851172920000000
O	2.2995380806743171	10.967719835284178	7.9823525719999999
O	5.2691607876743163	10.966432442284180	7.9830763500000002
O	8.2342295066743176	10.968552161284180	7.9917352360000002
O	11.197682422674315	10.972220996284179	7.9820246980000000
O	14.179448213674316	10.971230694284181	7.9735160489999997
O	2.2998461056743160	3.6882550802841791	4.5247139409999999
O	5.2698445496743176	3.6882550802841791	4.5247139409999999
O	8.2398443226743154	3.6882550802841791	4.5247139409999999
O	11.209846749674316	3.6882550802841791	4.5247139409999999
O	14.179843866674318	3.6882550802841791	4.5247139409999999
O	2.2998461056743160	10.281319856284181	4.5247139409999999
O	5.2698445496743176	10.281319856284181	4.5247139409999999
O	8.2398443226743154	10.281319856284181	4.5247139409999999
O	11.209846749674316	10.281319856284181	4.5247139409999999
O	14.179843866674318	10.281319856284181	4.5247139409999999
O	2.2934081206743180	1.1154087442841796	11.460458731999999
O	2.2934081206743180	14.301535939284179	11.460458731999999
O	5.2569831836743148	1.1165204772841797	11.462665028000000
O	5.2569831836743148	14.302648850284179	11.462665028000000
O	8.2329469546743148	1.1202235012841797	11.471044229000000
O	8.2329469546743148	14.306350696284181	11.471044229000000
O	11.201506179674315	1.1144655992841805	11.479807996000000
O	11.201506179674315	14.300592793284181	11.479807996000000
O	14.172116690674315	1.1101884342841792	11.459873850999999
O	14.172116690674315	14.296315629284180	11.459873850999999
O	2.3231112776743181	7.6949252372841812	11.492056482000001
O	5.2600275846743152	7.6842629792841812	11.466573999000000
O	8.2042820536743157	7.6764961762841786	11.484243265000000
O	11.100604133674317	7.6520828592841781	11.563123781000000
O	14.261932496674316	7.6700898612841790	11.535935789000000
O	2.3174778746743172	6.9680690372841809	8.0349219450000007
O	5.2724574506743167	6.9634299412841791	8.0024634090000006
O	8.2332443576743159	6.9612100132841803	8.0076186770000000

O	11.183229151674315	6.9628664122841784	8.0359830460000001
O	14.178810920674316	6.9554403212841791	7.9850776070000000
O	2.2996708496743175	0.37398684328417886	7.9926319269999997
O	2.2996708496743175	13.560115217284181	7.9926319269999997
O	5.2678437156743172	0.37216893028417886	7.9872782339999997
O	5.2678437156743172	13.558297304284181	7.9872782339999997
O	8.2322114126743173	0.37341977728418030	7.9901638989999997
O	8.2322114126743173	13.559546972284181	7.9901638989999997
O	11.206046890674315	0.37313211828417892	8.0053405699999995
O	11.206046890674315	13.559259312284180	8.0053405699999995
O	14.172804435674315	0.37453504628417988	8.0053793100000004
O	14.172804435674315	13.560662241284181	8.0053793100000004
O	2.2998461056743160	1.0937175792841796	4.5247139409999999
O	2.2998461056743160	14.279845953284180	4.5247139409999999
O	5.2698445496743176	1.0937175792841796	4.5247139409999999
O	5.2698445496743176	14.279845953284180	4.5247139409999999
O	8.2398443226743154	1.0937175792841796	4.5247139409999999
O	8.2398443226743154	14.279845953284180	4.5247139409999999
O	11.209846749674316	1.0937175792841796	4.5247139409999999
O	11.209846749674316	14.279845953284180	4.5247139409999999
O	14.179843866674318	1.0937175792841796	4.5247139409999999
O	14.179843866674318	14.279845953284180	4.5247139409999999
O	2.2998461056743160	7.6867811772841783	4.5247139409999999
O	5.2698445496743176	7.6867811772841783	4.5247139409999999
O	8.2398443226743154	7.6867811772841783	4.5247139409999999
O	11.209846749674316	7.6867811772841783	4.5247139409999999
O	14.179843866674318	7.6867811772841783	4.5247139409999999
O	0.82662551567431564	5.6050070562841796	12.729756428000000
O	15.676624376674315	5.6050070562841796	12.729756428000000
O	3.7898261696743170	5.6029168102841780	12.731433591000000
O	6.7333968766743162	5.5713886392841800	12.687944520000000
O	9.6916717876743164	5.5322516432841802	12.725141631000000
O	12.685729364674316	5.6339498292841803	12.764782203999999
O	0.82299692967431604	12.289953054284180	12.734768074000000
O	15.672997117674317	12.289953054284180	12.734768074000000
O	3.7918920606743178	12.263785486284181	12.726129975999999
O	6.7350193176743147	12.337308383284181	12.767470389000000
O	9.6813025016743168	12.275346090284181	12.750694980000000
O	12.685886032674315	12.246320791284180	12.737889014000000
O	0.81511441367431559	5.6419500602841808	10.088793625999999
O	15.665113274674315	5.6419500602841808	10.088793625999999
O	3.7764191216743157	5.6418805032841810	10.081712691000000
O	6.7411625556743147	5.6459843642841783	10.068206569000001
O	9.7113933456743169	5.6451249232841789	10.094598972000000
O	12.692136813674317	5.6795674132841789	10.112718000999999
O	0.81590970167431642	12.251559964284180	10.088948586000001
O	15.665909890674317	12.251559964284180	10.088948586000001
O	3.7827668236743150	12.249560495284179	10.085681189000001
O	6.7452027266743180	12.253318930284181	10.100514869000000
O	9.7079758626743171	12.259817201284179	10.099679593999999
O	12.679688359674316	12.218408404284180	10.103137857000000
O	3.7848446636743169	2.3909869192841793	2.4691270660000000
O	6.7548431086743150	2.3909869192841793	2.4691270660000000
O	9.7248455356743158	2.3909869192841793	2.4691270660000000
O	12.694845307674317	2.3909869192841793	2.4691270660000000
O	3.7848446636743169	8.9840516952841796	2.4691270660000000
O	6.7548431086743150	8.9840516952841796	2.4691270660000000
O	9.7248455356743158	8.9840516952841796	2.4691270660000000
O	12.694845307674317	8.9840516952841796	2.4691270660000000
O	0.81484489167431562	5.6875193072841803	3.2807229809999998
O	15.664845080674315	5.6875193072841803	3.2807229809999998
O	3.7848446636743169	5.6875193072841803	3.2807229809999998
O	6.7548431086743150	5.6875193072841803	3.2807229809999998
O	9.7248455356743158	5.6875193072841803	3.2807229809999998
O	12.694845307674317	5.6875193072841803	3.2807229809999998
O	0.81484489167431562	12.280582905284181	3.2807229809999998
O	15.664845080674315	12.280582905284181	3.2807229809999998
O	3.7848446636743169	12.280582905284181	3.2807229809999998

O	6.7548431086743150	12.280582905284181	3.2807229809999998		
O	9.7248455356743158	12.280582905284181	3.2807229809999998		
O	12.694845307674317	12.280582905284181	3.2807229809999998		
O	0.81160266467431619	2.3710429312841796	9.2162314209999998		
O	15.661602852674317	2.3710429312841796	9.2162314209999998		
O	3.7869583516743148	2.3612766612841796	9.2047218310000005		
O	6.7494818826743170	2.3617423392841790	9.2013306549999996		
O	9.7145691886743180	2.3565597552841790	9.2136934719999992		
O	12.692067773674317	2.3663024472841805	9.2181977170000007		
O	0.80799399367431590	8.9641890542841800	9.2162522080000002		
O	15.657994181674315	8.9641890542841800	9.2162522080000002		
O	3.7866636036743166	8.9757921002841812	9.2264540830000001		
O	6.7501364356743174	8.9669383232841788	9.2324106100000005		
O	9.7210111576743152	8.9703336462841783	9.2399753229999995		
O	12.698313243674317	8.9657641072841798	9.1738459700000003		
O	0.93440235867431554	5.6785830052841781	6.0396593129999996		
O	15.784401218674315	5.6785830052841781	6.0396593129999996		
O	3.8113852556743169	5.6843161502841788	5.9670665050000000		
O	6.7562557746743153	5.6845283572841794	5.9632260380000002		
O	9.6990829726743151	5.6804527912841785	5.9704935859999999		
O	12.573032090674317	5.6785818262841801	6.0438224570000001		
O	0.81719889267431611	12.282490416284180	5.9566690390000003		
O	15.667197752674316	12.282490416284180	5.9566690390000003		
O	3.7874137506743182	12.277329053284181	5.9578340770000002		
O	6.7540783566743166	12.276185489284181	5.9576862029999997		
O	9.7203632426743170	12.280924795284179	5.9588077759999996		
O	12.691512797674317	12.280863490284180	5.9574055740000000		
O	0.81321979467431582	2.3705748952841805	6.5616670639999999		
O	15.663219983674317	2.3705748952841805	6.5616670639999999		
O	3.7820419036743154	2.3712905072841792	6.5509677090000000		
O	6.7538951356743162	2.3721664532841800	6.5472207850000004		
O	9.7239320826743167	2.3731249252841788	6.5566454959999998		
O	12.686953498674317	2.3716170712841791	6.5636669029999997		
O	0.82019284067431641	8.9665068342841785	6.5629908410000004		
O	15.670193029674316	8.9665068342841785	6.5629908410000004		
O	3.7865281796743169	8.9657617492841801	6.5661283170000004		
O	6.7572130416743157	8.9658301272841783	6.5694188630000001		
O	9.7172006766743166	8.9655071002841780	6.5710024830000000		
O	12.682800473674316	8.9671788252841793	6.5503275519999997		
Au	7.8316702006743171	7.1784093182841779	21.549385586000000		
Au	7.5791097156743170	7.6496118182841784	15.006333614000001		
Au	6.7477346376743164	11.930921190284181	14.987823404000000		
Au	7.0448737576743170	10.601869031284181	17.328866953999999		
Au	7.4155192966743151	8.9593082772841797	19.487178169000000		
Au	5.5468319386743161	9.5573968392841806	15.157986938000001		
Au	5.8896450396743170	7.9359766882841782	17.433236567000002		
Au	6.5203804136743173	6.0772423002841798	19.445372875000000		
Au	4.7220448756743174	7.0590778502841793	15.117479910000000		
Au	5.2886938136743176	5.0923639272841790	17.305811868999999		
Au	4.0407150886743182	4.4687915772841791	15.025564767000001		
Au	8.7575119946743172	10.159519705284179	15.026029648000000		
Au	9.0699474696743181	8.5893241382841801	17.343641106000000		
Au	9.3111760906743157	6.6624427812841809	19.366792831000001		
Au	8.1030321516743165	5.4012817612841779	17.234089768000000		
Au	6.7403845266743154	4.9257985602841785	14.897129992000000		
Au	10.469580650674317	8.1431527162841810	15.042035925000000		
Au	10.828211891674318	6.2336134442841811	17.158580585999999		
Au	9.3945393066743179	5.4038023172841783	14.864965314000001		
Au	12.133870931674316	6.0218395832841800	14.845748334000000		
H	1.5311388915156865	2.9582482094017024	11.294792052341311	nuclear_charge=	0.66666666666666663
H	14.929672213939618	2.9595362703851293	11.294882443437029	nuclear_charge=	0.66666666666666663
H	1.5488782927438418	9.5598061553296780	11.316214013369637	nuclear_charge=	0.66666666666666663
H	14.996130157041907	9.5477151365789013	11.323560551372175	nuclear_charge=	0.66666666666666663
H	1.5567936596287559	5.0217948118339919	8.0445672801320871	nuclear_charge=	0.66666666666666663
H	14.935279126806172	5.0271822442719589	8.0314223725373015	nuclear_charge=	0.66666666666666663
H	1.5388985465748704	11.627371231587240	8.0671800116666574	nuclear_charge=	0.66666666666666663
H	14.938469637578326	11.632093935703306	8.0632624372044113	nuclear_charge=	0.66666666666666663
H	1.5433008653870832	3.0273518814665099	4.4162961325398351	nuclear_charge=	0.66666666666666663

H	14.936389106961549	3.0273518814665099	4.4162961325398342	nuclear_charge=	0.66666666666666663
H	1.5433008653870832	9.6204166574665102	4.4162961325398351	nuclear_charge=	0.66666666666666663
H	14.936389106961549	9.6204166574665102	4.4162961325398351	nuclear_charge=	0.66666666666666663
H	2.2880097869088125	0.10607272066509132	11.506500120091200	nuclear_charge=	0.66666666666666663
H	1.5275483308385009	1.7507667009088355	11.285281081409577	nuclear_charge=	0.66666666666666663
H	1.5275483308385009	14.936893895908835	11.285281081409577	nuclear_charge=	0.66666666666666663
H	3.0555106716672249	14.938722815303020	11.275794856811991	nuclear_charge=	0.66666666666666663
H	5.2471242837635756	0.10668310688628146	11.494905490782170	nuclear_charge=	0.66666666666666663
H	4.4926095744864654	14.936972461958479	11.277530749030351	nuclear_charge=	0.66666666666666663
H	6.0224682303623851	14.934864643063257	11.274931188062485	nuclear_charge=	0.66666666666666663
H	8.2389573923252755	0.11033313568507808	11.502563811950225	nuclear_charge=	0.66666666666666663
H	7.4672218099567615	14.936952203049646	11.278911716362476	nuclear_charge=	0.66666666666666663
H	8.9992369552536644	14.938580827201470	11.286672711209398	nuclear_charge=	0.66666666666666663
H	11.202180932023655	0.10515438437986191	11.526696973144244	nuclear_charge=	0.66666666666666663
H	10.440957984638079	14.938190709864266	11.290220285955652	nuclear_charge=	0.66666666666666663
H	11.966843378226891	14.934113777408353	11.295910483276097	nuclear_charge=	0.66666666666666663
H	14.175353529527889	0.10099749640957612	11.509182407924902	nuclear_charge=	0.66666666666666663
H	14.933889929634638	1.7503621861319996	11.284410863880254	nuclear_charge=	0.66666666666666663
H	14.933889929634638	14.936489381131999	11.284410863880254	nuclear_charge=	0.66666666666666663
H	13.409136031837342	14.935297196087497	11.285313742366096	nuclear_charge=	0.66666666666666663
H	1.5569498704819900	8.3287300333158996	11.312623099776218	nuclear_charge=	0.66666666666666663
H	14.990858422665337	8.3380460386868300	11.327585844224942	nuclear_charge=	0.66666666666666663
H	1.5556964055164393	6.3046243690731067	8.0558581688706479	nuclear_charge=	0.66666666666666663
H	14.942018656193454	6.2949439797154767	8.0316253805032218	nuclear_charge=	0.66666666666666663
H	1.5406947830911442	-0.28825127306015652	8.0720307154960569	nuclear_charge=	0.66666666666666663
H	3.0563216520020191	-0.29167389565202306	8.0653196424391211	nuclear_charge=	0.66666666666666663
H	2.2972843101349625	14.565880458730170	7.8959947523304272	nuclear_charge=	0.66666666666666663
H	1.5406947833419036	12.897877100649310	8.0720307154698236	nuclear_charge=	0.66666666666666663
H	4.5090147299041305	-0.29072562958349124	8.0624948069931985	nuclear_charge=	0.66666666666666663
H	6.0251182286037590	-0.29176795877162043	8.0687109020048151	nuclear_charge=	0.66666666666666663
H	5.2664823670509904	14.563762565092802	7.8875490204415897	nuclear_charge=	0.66666666666666663
H	7.4753615074163449	-0.29117974074566710	8.0701255244555572	nuclear_charge=	0.66666666666666663
H	8.9915146084925226	-0.28838518192563711	8.0700451407627103	nuclear_charge=	0.66666666666666663
H	8.2352818784077151	14.565058751732675	7.8909429655711074	nuclear_charge=	0.66666666666666663
H	10.447211736461746	-0.29007852553471913	8.0776524684435227	nuclear_charge=	0.66666666666666663
H	11.962712268662884	-0.29188226656202509	8.0835808007067413	nuclear_charge=	0.66666666666666663
H	11.207477175449995	14.564746753367576	7.9058361976888909	nuclear_charge=	0.66666666666666663
H	14.931251093941569	-0.28904200481180808	8.0784020723552423	nuclear_charge=	0.66666666666666663
H	13.417577046386125	-0.29210173876652412	8.0837064910178800	nuclear_charge=	0.66666666666666663
H	14.174454027264389	14.565943899371334	7.9038203283138211	nuclear_charge=	0.66666666666666663
H	14.931251390199236	12.897085531939926	8.0784021008787423	nuclear_charge=	0.66666666666666663
H	2.2998461056743160	8.7782883179054139E-002	4.6196009642007017	nuclear_charge=	0.66666666666666663
H	1.5433011595635477	1.7546211217653589	4.4162961746972185	nuclear_charge=	0.66666666666666663
H	1.5433008653870814	14.940749152101848	4.4162961325398351	nuclear_charge=	0.66666666666666663
H	3.0563907514523976	14.940749814810303	4.4162960238258826	nuclear_charge=	0.66666666666666663
H	5.2698445496743176	8.7782883179054139E-002	4.6196009642007017	nuclear_charge=	0.66666666666666663
H	4.5132996066414357	14.940749483455974	4.4162960781828753	nuclear_charge=	0.66666666666666663
H	6.0263891956762343	14.940749814560792	4.4162960238668143	nuclear_charge=	0.66666666666666663
H	8.2398443226743154	8.7782883179054139E-002	4.6196009642007017	nuclear_charge=	0.66666666666666663
H	7.4832990823870809	14.940749152101848	4.4162961325398351	nuclear_charge=	0.66666666666666663
H	8.9963895627377113	14.940749152351362	4.4162961324989034	nuclear_charge=	0.66666666666666663
H	11.209846749674316	8.7782883179054139E-002	4.6196009642007017	nuclear_charge=	0.66666666666666663
H	10.453301509387082	14.940749152101848	4.4162961325398351	nuclear_charge=	0.66666666666666663
H	11.966391395452398	14.940749814810303	4.4162960238258826	nuclear_charge=	0.66666666666666663
H	14.179843866674318	8.7782883179054139E-002	4.6196009642007017	nuclear_charge=	0.66666666666666663
H	14.936388812785086	1.7546211217653589	4.4162961746972176	nuclear_charge=	0.66666666666666663
H	14.936389106961553	14.940749152101851	4.4162961325398342	nuclear_charge=	0.66666666666666663
H	13.423299220672398	14.940749814560792	4.4162960238668143	nuclear_charge=	0.66666666666666663
H	1.5433014534904252	8.3476850631369786	4.4162962168188340	nuclear_charge=	0.66666666666666663
H	14.936388518858209	8.3476850631369786	4.4162962168188340	nuclear_charge=	0.66666666666666663
H	4.9129150296540303E-002	5.6407184938167241	12.085448850011266	nuclear_charge=	0.66666666666666663
H	16.450698020305573	5.6299092461006808	12.080835758597514	nuclear_charge=	0.66666666666666663
H	2.8891199061931161E-002	12.252300142793263	12.111160419408726	nuclear_charge=	0.66666666666666663
H	16.458346355275495	12.263816071364648	12.099589679276821	nuclear_charge=	0.66666666666666663
H	9.6062188404056670E-002	5.6569104640164838	10.798475416420223	nuclear_charge=	0.66666666666666663
H	0.81786624165522070	5.6532277925060583	9.0784603145632765	nuclear_charge=	0.66666666666666663
H	16.386203639480854	5.6466748020946156	10.796546776892310	nuclear_charge=	0.66666666666666663
H	15.667865768765765	5.6532277924858079	9.0784603163775444	nuclear_charge=	0.66666666666666663

H	9.7992113405451775E-002	12.235891722100671	10.799762909604340	nuclear_charge= 0.66666666666666663
H	0.81258715656348279	12.255904951770475	9.0785633912461741	nuclear_charge= 0.66666666666666663
H	16.380604334480765	12.246600390047892	10.803158229323016	nuclear_charge= 0.66666666666666663
H	15.662587345042787	12.255904951770468	9.0785633912478847	nuclear_charge= 0.66666666666666663
H	3.0720806610080125	2.3909869192841793	1.7529737049779972	nuclear_charge= 0.66666666666666663
H	4.4976089865565285	2.3909869192841793	1.7529740236785547	nuclear_charge= 0.66666666666666663
H	6.0420791057668879	2.3909869192841793	1.7529737052179819	nuclear_charge= 0.66666666666666663
H	7.4676077517720074	2.3909869192841793	1.7529743423789714	nuclear_charge= 0.66666666666666663
H	9.0120808928177496	2.3909869192841793	1.7529743421389861	nuclear_charge= 0.66666666666666663
H	10.437610178772008	2.3909869192841793	1.7529743423789714	nuclear_charge= 0.66666666666666663
H	11.982081305008013	2.3909869192841793	1.7529737049779972	nuclear_charge= 0.66666666666666663
H	13.407609310581748	2.3909869192841793	1.7529737052179830	nuclear_charge= 0.66666666666666663
H	3.0720806610080125	8.9840516952841796	1.7529737049779972	nuclear_charge= 0.66666666666666663
H	4.4976089865565285	8.9840516952841796	1.7529740236785547	nuclear_charge= 0.66666666666666663
H	6.0420791057668879	8.9840516952841796	1.7529737052179819	nuclear_charge= 0.66666666666666663
H	7.4676077517720074	8.9840516952841796	1.7529743423789714	nuclear_charge= 0.66666666666666663
H	9.0120808928177496	8.9840516952841796	1.7529743421389861	nuclear_charge= 0.66666666666666663
H	10.437610178772008	8.9840516952841796	1.7529743423789714	nuclear_charge= 0.66666666666666663
H	11.982081305008013	8.9840516952841796	1.7529737049779972	nuclear_charge= 0.66666666666666663
H	13.407609310581748	8.9840516952841796	1.7529737052179830	nuclear_charge= 0.66666666666666663
H	8.7663592089814557E-002	5.6875193072841803	3.9822324374826583	nuclear_charge= 0.66666666666666663
H	0.81484489167431562	5.6875193072841803	2.2703229809999996	nuclear_charge= 0.66666666666666663
H	16.392025753556347	5.6875193072841803	3.9822330871188178	nuclear_charge= 0.66666666666666663
H	15.664845080674315	5.6875193072841803	2.2703229809999996	nuclear_charge= 0.66666666666666663
H	3.7848446636743169	5.6875193072841803	2.2703229809999996	nuclear_charge= 0.66666666666666663
H	6.7548431086743150	5.6875193072841803	2.2703229809999996	nuclear_charge= 0.66666666666666663
H	9.7248455356743158	5.6875193072841803	2.2703229809999996	nuclear_charge= 0.66666666666666663
H	12.694845307674317	5.6875193072841803	2.2703229809999996	nuclear_charge= 0.66666666666666663
H	8.7663592089814557E-002	12.280582905284181	3.9822324374826583	nuclear_charge= 0.66666666666666663
H	0.81484489167431562	12.280582905284181	2.2703229809999996	nuclear_charge= 0.66666666666666663
H	16.392025753556347	12.280582905284181	3.9822330871188178	nuclear_charge= 0.66666666666666663
H	15.664845080674315	12.280582905284181	2.2703229809999996	nuclear_charge= 0.66666666666666663
H	3.7848446636743169	12.280582905284181	2.2703229809999996	nuclear_charge= 0.66666666666666663
H	6.7548431086743150	12.280582905284181	2.2703229809999996	nuclear_charge= 0.66666666666666663
H	9.7248455356743158	12.280582905284181	2.2703229809999996	nuclear_charge= 0.66666666666666663
H	12.694845307674317	12.280582905284181	2.2703229809999996	nuclear_charge= 0.66666666666666663
H	7.9394247649085870E-002	2.3712317862218573	8.5199707238676563	nuclear_charge= 0.66666666666666663
H	0.80613527574595167	2.3616006194258716	10.226572507170941	nuclear_charge= 0.66666666666666663
H	16.392627592139000	2.3701754868245146	8.5187285623458564	nuclear_charge= 0.66666666666666663
H	15.656135463745951	2.3616006194258716	10.226572507170941	nuclear_charge= 0.66666666666666663
H	7.1899528201905483E-002	8.9629465172239904	8.5241022574816601	nuclear_charge= 0.66666666666666663
H	0.80766854996478799	8.9561013526935049	10.226619786245349	nuclear_charge= 0.66666666666666663
H	16.390560139894117	8.9640967789988153	8.5203676857243753	nuclear_charge= 0.66666666666666663
H	15.657668737964787	8.9561013526935049	10.226619786245349	nuclear_charge= 0.66666666666666663
H	0.15563368411332590	5.6829202178465863	5.3959134779768068	nuclear_charge= 0.66666666666666663
H	0.87802470137111044	5.6715653692829520	7.0484608160430691	nuclear_charge= 0.66666666666666663
H	16.509150711567262	5.6833262154320217	5.3356537487400928	nuclear_charge= 0.66666666666666663
H	15.728024217193337	5.6715653690280057	7.0484608526922177	nuclear_charge= 0.66666666666666663
H	4.1991015708891410E-002	12.281496223680637	5.3086258930044377	nuclear_charge= 0.66666666666666663
H	0.81366334466861723	12.272079345828963	6.9670092141451221	nuclear_charge= 0.66666666666666663
H	16.441393119126619	12.281494368480992	5.3074166208662223	nuclear_charge= 0.66666666666666663
H	15.663662816806589	12.272079345806898	6.9670092162868027	nuclear_charge= 0.66666666666666663
H	3.7700533698087568E-002	2.3710187772229112	7.2093381524574900	nuclear_charge= 0.66666666666666663
H	0.81394961809715483	2.3797418393021452	5.5513089124046644	nuclear_charge= 0.66666666666666663
H	16.438275130902561	2.3698990198847838	7.2098932760181862	nuclear_charge= 0.66666666666666663
H	15.663949807097154	2.3797418393021452	5.5513089124046644	nuclear_charge= 0.66666666666666663
H	4.2010101547077738E-002	8.9640068787300891	7.2074545985121317	nuclear_charge= 0.66666666666666663
H	0.81779249454719150	8.9743815801264262	5.5526243794048078	nuclear_charge= 0.66666666666666663
H	16.439559859648444	8.9651932211272047	7.2179573404150750	nuclear_charge= 0.66666666666666663
H	15.667792683547191	8.9743815801264262	5.5526243794048078	nuclear_charge= 0.66666666666666663

Table S4. Cartesian coordinate of cluster Cu_{20}-TiO_2.

Ti	2.2622719386743171	5.6123360812841803	11.570243378000001
Ti	5.2648838446743156	5.5897433292841789	11.454555743000000
Ti	8.2509838916743163	5.6065136322841802	11.387963855000001
Ti	11.227166731674316	5.6162103452841805	11.430326515000001
Ti	14.197686295674316	5.6355194792841807	11.549588770000000

Ti	2.2874343846743166	12.245964361284180	11.580206780999999
Ti	5.2523826156743176	12.235567362284179	11.589704578999999
Ti	8.2384498016743173	12.231961010284181	11.464906373000000
Ti	11.258703434674317	12.243509825284178	11.511075055999999
Ti	14.197160529674317	12.236193375284181	11.560828624000001
Ti	2.2998445016743148	5.6875189142841798	4.7132990990000003
Ti	5.2698451036743172	5.6875189142841798	4.7132990990000003
Ti	8.2398445436743160	5.6875189142841798	4.7132990990000003
Ti	11.209845643674317	5.6875189142841798	4.7132990990000003
Ti	14.179843423674317	5.6875189142841798	4.7132990990000003
Ti	2.2998445016743148	12.280582512284180	4.7132990990000003
Ti	5.2698451036743172	12.280582512284180	4.7132990990000003
Ti	8.2398445436743160	12.280582512284180	4.7132990990000003
Ti	11.209845643674317	12.280582512284180	4.7132990990000003
Ti	14.179843423674317	12.280582512284180	4.7132990990000003
Ti	2.3152369496743148	2.3598502032841804	7.8271696390000001
Ti	5.2507455696743150	2.3627357862841798	7.8181145860000001
Ti	8.2253766676743147	2.3718943172841804	7.8023423080000001
Ti	11.218158995674315	2.3725008772841800	7.8009515560000002
Ti	14.200783804674316	2.3637527622841805	7.8182512820000003
Ti	2.3039188606743153	8.9605333832841794	7.8145679579999996
Ti	5.2774663956743169	8.9551662972841797	7.8394666690000001
Ti	8.2374931986743150	8.9437748692841801	7.8599346609999996
Ti	11.206114160674318	8.9447074042841805	7.8245845469999997
Ti	14.182793558674316	8.9580641112841803	7.8182480170000002
Ti	3.7808435496743158	2.3138627292841800	11.174197627000000
Ti	6.7481822916743148	2.3208859202841801	11.130091977999999
Ti	9.7303583406743179	2.3244278732841792	11.105346758000000
Ti	12.709823902674316	2.3417329702841805	11.120923556999999
Ti	3.7777460406743160	8.9255603742841814	11.130255267000001
Ti	6.7560847226743164	8.9366847742841813	11.201074929000001
Ti	9.7430205526743165	8.9183789112841794	11.178527107000001
Ti	12.709383108674317	8.9411800402841806	11.131829369000000
Ti	3.7788148336743177	5.6552456592841800	8.1249025939999999
Ti	6.7412490766743147	5.6602617182841790	8.0452812100000006
Ti	9.7289217756743156	5.6587532752841803	7.9979872350000001
Ti	12.712373074674318	5.6596463162841779	8.0691726639999999
Ti	3.7879281216743159	12.256753944284181	8.1613196820000002
Ti	6.7570725266743175	12.253299674284179	8.1292470029999997
Ti	9.7268970436743167	12.252729071284179	8.1158256140000002
Ti	12.695977387674315	12.253177065284181	8.1222125310000006
Ti	3.7848445536743149	2.3909869682841798	4.3119035480000001
Ti	6.7548446576743153	2.3909869682841798	4.3119035480000001
Ti	9.7248457576743164	2.3909869682841798	4.3119035480000001
Ti	12.694844865674316	2.3909869682841798	4.3119035480000001
Ti	3.7848445536743149	8.9840518922841781	4.3119035480000001
Ti	6.7548446576743153	8.9840518922841781	4.3119035480000001
Ti	9.7248457576743164	8.9840518922841781	4.3119035480000001
Ti	12.694844865674316	8.9840518922841781	4.3119035480000001
O	2.2246773036743157	3.6109350402841791	11.585599037000000
O	5.3524004176743176	3.6050153292841784	11.516644597000001
O	8.2597214416743157	3.5733409712841784	11.441599026000000
O	11.207914513674318	3.5802951942841794	11.472109733000000
O	14.163966865674315	3.5923889712841799	11.518901712000000
O	2.3053945916743181	10.171753087284181	11.494792851000000
O	5.2505142186743150	10.172656148284180	11.477920941000001
O	8.2740153896743180	10.185935635284181	11.424842817000000
O	11.218597134674315	10.183740464284181	11.502158566000000
O	14.182241238674315	10.174592544284181	11.495501989999999
O	2.2957966956743157	4.3659656442841808	8.0103775729999995
O	5.2474791116743162	4.3581811582841787	8.0309817950000006
O	8.2165740606743149	4.3505629022841781	8.0346954440000005
O	11.238817241674315	4.3499398362841788	8.0342820899999996
O	14.207428910674317	4.3562032062841780	8.0471081760000001
O	2.3073645566743153	10.956736514284181	8.0074924969999994
O	5.2712142866743150	10.957816416284178	8.0188564499999995
O	8.2410939026743151	10.956425276284179	8.0153032900000003
O	11.209797182674315	10.956438245284179	7.9968050259999996

O	14.181141907674316	10.956533738284179	7.9924316910000002
O	2.2998445016743148	3.6882561612841798	4.5247138380000003
O	5.2698451036743172	3.6882561612841798	4.5247138380000003
O	8.2398445436743160	3.6882561612841798	4.5247138380000003
O	11.209845643674317	3.6882561612841798	4.5247138380000003
O	14.179843423674317	3.6882561612841798	4.5247138380000003
O	2.2998445016743148	10.281320642284179	4.5247138380000003
O	5.2698451036743172	10.281320642284179	4.5247138380000003
O	8.2398445436743160	10.281320642284179	4.5247138380000003
O	11.209845643674317	10.281320642284179	4.5247138380000003
O	14.179843423674317	10.281320642284179	4.5247138380000003
O	2.2023537146743166	1.0643617132841801	11.579336220000000
O	2.2023537146743166	14.250489694284180	11.579336220000000
O	5.3791248946743160	1.0512389692841797	11.618704633000000
O	5.3791248946743160	14.237366949284180	11.618704633000000
O	8.2570740216743168	1.0818827012841794	11.536185110000000
O	8.2570740216743168	14.268010682284181	11.536185110000000
O	11.220004489674317	1.0987553882841805	11.493131040000000
O	11.220004489674317	14.284883368284181	11.493131040000000
O	14.164385088674315	1.0985932852841795	11.508545483000001
O	14.164385088674315	14.284721265284180	11.508545483000001
O	2.2995706646743166	7.6811142482841781	11.505697730000000
O	5.2684818936743163	7.6650630932841786	11.486735725000001
O	8.2502682646743182	7.6639442872841812	11.494033326000000
O	11.237158952674317	7.6684448592841790	11.517040222000000
O	14.177104392674316	7.6907378682841809	11.513180080000000
O	2.3020929506743180	6.9631796152841794	8.0388065839999996
O	5.2415775146743151	6.9652810602841804	8.0655112669999998
O	8.2185496686743171	6.9640402352841804	8.0527542289999996
O	11.239749946674316	6.9655569302841798	8.0454491640000008
O	14.204926208674316	6.9699071882841785	8.0623276060000002
O	2.2994800496743153	0.35282855628418019	8.0014181620000002
O	2.2994800496743153	13.538956536284179	8.0014181620000002
O	5.2741840056743179	0.35196204128417996	7.9922161500000000
O	5.2741840056743179	13.538090021284180	7.9922161500000000
O	8.2406079676743147	0.35842730228417885	7.9995958610000004
O	8.2406079676743147	13.544555283284179	7.9995958610000004
O	11.212266692674316	0.36157033428417940	7.9985293530000003
O	11.212266692674316	13.547698315284180	7.9985293530000003
O	14.183550343674316	0.35896725328417922	8.0063000239999997
O	14.183550343674316	13.545095234284179	8.0063000239999997
O	2.2998445016743148	1.0937170762841788	4.5247138380000003
O	2.2998445016743148	14.279845056284181	4.5247138380000003
O	5.2698451036743172	1.0937170762841788	4.5247138380000003
O	5.2698451036743172	14.279845056284181	4.5247138380000003
O	8.2398445436743160	1.0937170762841788	4.5247138380000003
O	8.2398445436743160	14.279845056284181	4.5247138380000003
O	11.209845643674317	1.0937170762841788	4.5247138380000003
O	11.209845643674317	14.279845056284181	4.5247138380000003
O	14.179843423674317	1.0937170762841788	4.5247138380000003
O	14.179843423674317	14.279845056284181	4.5247138380000003
O	2.2998445016743148	7.6867813732841803	4.5247138380000003
O	5.2698451036743172	7.6867813732841803	4.5247138380000003
O	8.2398445436743160	7.6867813732841803	4.5247138380000003
O	11.209845643674317	7.6867813732841803	4.5247138380000003
O	14.179843423674317	7.6867813732841803	4.5247138380000003
O	0.79462677667431691	5.6792475292841793	12.766652364000000
O	15.644626521674315	5.6792475292841793	12.766652364000000
O	3.8106525906743158	5.6515836022841803	12.794166025000001
O	6.7953724996743148	5.5808724572841797	12.712908956000000
O	9.7058491196743155	5.5543550412841789	12.704393690000000
O	12.665829452674316	5.5963922092841791	12.790151925000000
O	0.79864437667431609	12.177013361284178	12.753973705000000
O	15.648644122674316	12.177013361284178	12.753973705000000
O	3.7957147116743180	12.210465548284180	12.764447501999999
O	6.8220936576743156	12.021206925284179	12.813328642000000
O	9.7275257066743173	12.280786467284180	12.758445012999999
O	12.678516891674317	12.222622692284180	12.730964942000000

O	0.81643988767431708	5.6471248822841780	10.134599808000001
O	15.666439633674315	5.6471248822841780	10.134599808000001
O	3.7628625966743172	5.6072466332841806	10.133718049000001
O	6.7395071426743165	5.6470942302841785	10.090305677000000
O	9.7477491326743149	5.6491667922841806	10.093293392000000
O	12.715700273674315	5.6440726282841780	10.126579072000000
O	0.81617968967431587	12.229219994284179	10.108417648000000
O	15.666179435674316	12.229219994284179	10.108417648000000
O	3.7984939056743166	12.274868230284181	10.131252858000000
O	6.7559366856743175	12.257618101284180	10.119758274000000
O	9.7322556146743153	12.249650880284179	10.100097395000001
O	12.699787868674317	12.237636387284180	10.094179816000000
O	3.7848445536743149	2.3909869682841798	2.4691269309999999
O	6.7548446576743153	2.3909869682841798	2.4691269309999999
O	9.7248457576743164	2.3909869682841798	2.4691269309999999
O	12.694844865674316	2.3909869682841798	2.4691269309999999
O	3.7848445536743149	8.9840518922841781	2.4691269309999999
O	6.7548446576743153	8.9840518922841781	2.4691269309999999
O	9.7248457576743164	8.9840518922841781	2.4691269309999999
O	12.694844865674316	8.9840518922841781	2.4691269309999999
O	0.81484444967431635	5.6875189142841798	3.2807232549999998
O	15.664844194674316	5.6875189142841798	3.2807232549999998
O	3.7848445536743149	5.6875189142841798	3.2807232549999998
O	6.7548446576743153	5.6875189142841798	3.2807232549999998
O	9.7248457576743164	5.6875189142841798	3.2807232549999998
O	12.694844865674316	5.6875189142841798	3.2807232549999998
O	0.81484444967431635	12.280582512284180	3.2807232549999998
O	15.664844194674316	12.280582512284180	3.2807232549999998
O	3.7848445536743149	12.280582512284180	3.2807232549999998
O	6.7548446576743153	12.280582512284180	3.2807232549999998
O	9.7248457576743164	12.280582512284180	3.2807232549999998
O	12.694844865674316	12.280582512284180	3.2807232549999998
O	0.82921067867431653	2.3469531322841792	9.2389012269999995
O	15.679210423674316	2.3469531322841792	9.2389012269999995
O	3.7868696176743164	2.3350723002841800	9.1859257420000002
O	6.7446838196743180	2.3226242552841789	9.2254938339999999
O	9.7260247486743161	2.3340520822841793	9.2207426029999997
O	12.704017899674316	2.3410425582841796	9.2290917819999994
O	0.82095300167431695	8.9676057942841787	9.2427539050000007
O	15.670952747674317	8.9676057942841787	9.2427539050000007
O	3.7745041446743173	8.9621673822841785	9.2399266799999999
O	6.7474135576743173	8.9602186082841797	9.2782192539999997
O	9.7420221276743177	8.9451341772841779	9.2664634090000000
O	12.703484166674315	8.9710918962841788	9.2428929330000003
O	0.83718301867431677	5.6869427112841784	5.9800033580000003
O	15.687182764674315	5.6869427112841784	5.9800033580000003
O	3.7643589076743176	5.6855783932841781	5.9801993050000002
O	6.6308294596743167	5.6877004702841809	6.0519711730000001
O	9.7246651906743153	5.6773627122841788	6.1167206980000000
O	12.822842479674318	5.6808272982841785	6.0580651029999997
O	0.81535971567431709	12.269309567284179	5.9629009780000004
O	15.665359461674317	12.269309567284179	5.9629009780000004
O	3.7855814236743157	12.268333411284178	5.9642422770000003
O	6.7552827966743152	12.266284428284180	5.9686380059999999
O	9.7245144976743170	12.270426015284180	5.9678938759999998
O	12.695511367674317	12.272855793284180	5.9657394000000004
O	0.81922366567431659	2.3666040082841793	6.5716931030000003
O	15.669223411674317	2.3666040082841793	6.5716931030000003
O	3.7801438546743178	2.3678775492841790	6.5521586559999996
O	6.7482891706743153	2.3677061622841791	6.5680307730000003
O	9.7257253536743171	2.3687084012841790	6.5747344700000001
O	12.710259386674316	2.3662600552841795	6.5744232890000003
O	0.81889923167431711	8.9621832982841809	6.5740299960000002
O	15.668898976674317	8.9621832982841809	6.5740299960000002
O	3.7908935246743170	8.9628458582841795	6.5813224650000004
O	6.7547663236743176	8.9606335922841787	6.6044669899999997
O	9.7164733226743181	8.9603671542841781	6.6033869530000002
O	12.700759739674318	8.9614334972841796	6.5828839700000001

Cu	12.018596161674317	5.9026209002841803	14.544506836000000	
Cu	7.0922726346743161	9.0462075302841782	16.326767300000000	
Cu	8.1212224366743158	7.8583678102841787	20.389381885999999	
Cu	9.4395695786743161	7.2369057482841797	18.499431195000000	
Cu	10.720574678674318	6.4728908002841798	16.522256403000000	
Cu	6.9022511576743177	7.4871116042841805	18.375264683000001	
Cu	8.1920449246743168	6.7098106852841788	16.483442237999999	
Cu	9.4878431846743148	5.9947532342841789	14.527941908000001	
Cu	5.6574270316743167	7.0569583272841783	16.334916800999999	
Cu	6.9209630056743165	6.2672615312841806	14.457798727000000	
Cu	4.4299787616743167	6.5609499252841808	14.337131255999999	
Cu	8.3390723676743157	9.4058512582841800	18.433257326000000	
Cu	9.7016987496743177	8.7410646352841788	16.511150913000002	
Cu	10.907500637674318	8.0106622092841810	14.555456502000000	
Cu	8.3903558546743149	8.3632406002841790	14.295575709000000	
Cu	5.8813319086743157	8.5287602542841796	14.304593905000001	
Cu	8.5635873206743156	10.959593066284178	16.434841955000000	
Cu	9.9628002866743159	10.180096976284180	14.536911584000000	
Cu	7.2312754986743180	10.635421034284178	14.254673316000000	
Cu	9.0548518166743150	12.338465701284179	14.556791735999999	
H	1.4949141734995113	2.9491547693975040	11.361097796685769	nuclear_charge= 0.66666666666666663
H	14.930028239999928	2.9597782178281307	11.334886045878543	nuclear_charge= 0.66666666666666663
H	1.5418631945595926	9.5358485893781264	11.311616883245259	nuclear_charge= 0.66666666666666663
H	14.946492775132842	9.5394725074481812	11.312607791540294	nuclear_charge= 0.66666666666666663
H	1.5380970463534567	5.0313846147000021	8.0737573428780500	nuclear_charge= 0.66666666666666663
H	14.960226099016506	5.0286687326305639	8.0917698945044805	nuclear_charge= 0.66666666666666663
H	1.5465534516736810	11.617502380511763	8.0814052329541557	nuclear_charge= 0.66666666666666663
H	14.940463345290265	11.618097319085841	8.0741185500027370	nuclear_charge= 0.66666666666666663
H	1.5432998920274414	3.0273522487937345	4.4162959793738370	nuclear_charge= 0.66666666666666663
H	14.936388491513917	3.0273527595480321	4.4162960631604298	nuclear_charge= 0.66666666666666663
H	1.5432998184208984	9.6204168157824270	4.4162959688255308	nuclear_charge= 0.66666666666666663
H	14.936388565120389	9.6204173265367565	4.4162960526121484	nuclear_charge= 0.66666666666666663
H	2.2452010741529733	5.4870717002474478E-002	11.579774639747930	nuclear_charge= 0.66666666666666663
H	1.4804700818596306	1.7354301474120266	11.356941666578672	nuclear_charge= 0.66666666666666663
H	1.4804701797176030	14.921558243671097	11.356941696726301	nuclear_charge= 0.66666666666666663
H	2.9790159998923222	14.865280281845500	11.379996338977781	nuclear_charge= 0.66666666666666663
H	5.3152868233802195	4.2963461428268346E-002	11.604097754094647	nuclear_charge= 0.66666666666666663
H	4.6045090978375285	14.849304273544803	11.403271955655875	nuclear_charge= 0.66666666666666663
H	6.0958423128982631	14.902042442859543	11.362910202539233	nuclear_charge= 0.66666666666666663
H	8.2478379213831552	7.2143486803989632E-002	11.500835807427817	nuclear_charge= 0.66666666666666663
H	7.4925612899856979	14.895778925193351	11.330429244273795	nuclear_charge= 0.66666666666666663
H	9.0108487154636627	14.903732929306511	11.315755766119349	nuclear_charge= 0.66666666666666663
H	11.239154662365493	8.8575907819203437E-002	11.502010687144253	nuclear_charge= 0.66666666666666663
H	10.455067208446962	14.914269625957424	11.294002803828986	nuclear_charge= 0.66666666666666663
H	11.981947650333538	14.920583098669276	11.302771819805773	nuclear_charge= 0.66666666666666663
H	14.180543227391315	8.8651370940045382E-002	11.534321522340958	nuclear_charge= 0.66666666666666663
H	14.932183923734545	1.7304722999369861	11.329319313812700	nuclear_charge= 0.66666666666666663
H	14.932183828674496	14.916600401738723	11.329319336002429	nuclear_charge= 0.66666666666666663
H	13.411578672101967	14.928106803928653	11.307932153854946	nuclear_charge= 0.66666666666666663
H	1.5429978775520645	8.3239474864761220	11.317891431751706	nuclear_charge= 0.66666666666666663
H	14.939391170125198	8.3258900047835773	11.322369198696544	nuclear_charge= 0.66666666666666663
H	1.5434983894886898	6.2975394579716770	8.0874443544317565	nuclear_charge= 0.66666666666666663
H	14.958176128830416	6.2974716445543670	8.0991128926458895	nuclear_charge= 0.66666666666666663
H	1.5389481138259260	-0.30786000634303967	8.0788129794510404	nuclear_charge= 0.66666666666666663
H	3.0624833803740614	-0.30445014289906780	8.0833863810917421	nuclear_charge= 0.66666666666666663
H	2.3073825031687107	14.545539138664118	7.9140272253143440	nuclear_charge= 0.66666666666666663
H	1.5389480143412424	12.878268089361750	8.0788129895750060	nuclear_charge= 0.66666666666666663
H	4.5117432038392629	-0.30535582285402896	8.0789652663176845	nuclear_charge= 0.66666666666666663
H	6.0359733391534149	-0.30806041817099228	8.0626116357374915	nuclear_charge= 0.66666666666666663
H	5.2624511042409878	14.544655907297969	7.9050633179333962	nuclear_charge= 0.66666666666666663
H	7.4801167167266343	-0.30349719237729289	8.0660577342747235	nuclear_charge= 0.66666666666666663
H	9.0018889272548215	-0.30324880255240139	8.0591289851282770	nuclear_charge= 0.66666666666666663
H	8.2330011046389728	14.550112725658384	7.9010843097960484	nuclear_charge= 0.66666666666666663
H	10.452008604087975	-0.30123543662108077	8.0585652488767749	nuclear_charge= 0.66666666666666663
H	11.972119102168673	-0.30139292465265122	8.0618712276602622	nuclear_charge= 0.66666666666666663
H	11.215212661710847	14.553252148602720	7.8997317337735042	nuclear_charge= 0.66666666666666663
H	14.942896809977324	-0.30339029864329881	8.0810233638462101	nuclear_charge= 0.66666666666666663
H	13.422001371888776	-0.30241769858592349	8.0656403055308949	nuclear_charge= 0.66666666666666663

H	14.192197851780705	14.551042543479142	7.9119422382747917	nuclear_charge=	0.66666666666666663
H	14.942896909083625	12.882737797075368	8.0810233735987467	nuclear_charge=	0.66666666666666663
H	2.2998446405445065	8.7782398217347790E-002	4.6196010524314515	nuclear_charge=	0.66666666666666663
H	1.5433000419851641	1.7546211639582925	4.4162960008637722	nuclear_charge=	0.66666666666666663
H	1.5432998461169198	14.940748915141000	4.4162959727945541	nuclear_charge=	0.66666666666666663
H	3.0563892807893929	14.940748777409453	4.4162959953887038	nuclear_charge=	0.66666666666666663
H	5.2698448249276311	8.7782398217376212E-002	4.6196010524314488	nuclear_charge=	0.66666666666666663
H	4.5133002623327556	14.940748708044666	4.4162960067676389	nuclear_charge=	0.66666666666666663
H	6.0263896970051682	14.940748984505817	4.4162959614156145	nuclear_charge=	0.66666666666666663
H	8.2398444324775326	8.7782398217344237E-002	4.6196010524314524	nuclear_charge=	0.66666666666666663
H	7.4832997766464011	14.940748790883191	4.4162959931784069	nuclear_charge=	0.66666666666666663
H	8.9963895085734435	14.940748570313168	4.4162960293617797	nuclear_charge=	0.66666666666666663
H	11.209846200161387	8.7782398217489899E-002	4.6196010524314381	nuclear_charge=	0.66666666666666663
H	10.453300876646402	14.940748790883191	4.4162959931784069	nuclear_charge=	0.66666666666666663
H	11.966390311318879	14.940748901667263	4.4162959750048518	nuclear_charge=	0.66666666666666663
H	14.179843646571037	8.7782398217362001E-002	4.6196010524314506	nuclear_charge=	0.66666666666666663
H	14.936388341556334	1.7546206532040625	4.4162960846503161	nuclear_charge=	0.66666666666666663
H	14.936388537424396	14.940748404386685	4.4162960565811629	nuclear_charge=	0.66666666666666663
H	13.423299102528862	14.940749287914606	4.4162959116429699	nuclear_charge=	0.66666666666666663
H	1.5433001615022022	8.3476856005804798	4.4162960179913568	nuclear_charge=	0.66666666666666663
H	14.936388222039408	8.3476850898263031	4.4162961017778599	nuclear_charge=	0.66666666666666663
H	2.1595464591859681E-002	5.6558854500563740	12.116433198030382	nuclear_charge=	0.66666666666666663
H	16.427291902502407	5.6435652430718122	12.128632116590973	nuclear_charge=	0.66666666666666663
H	1.8494202026177575E-002	12.208821922593078	12.112676519271000	nuclear_charge=	0.66666666666666663
H	16.441578486325565	12.213737073921319	12.128822024308718	nuclear_charge=	0.66666666666666663
H	8.8797055753932597E-002	5.6413751446719935	10.835606947530172	nuclear_charge=	0.66666666666666663
H	0.81804479948016962	5.6548184329436459	9.1242303738144912	nuclear_charge=	0.66666666666666663
H	16.383317807605284	5.6298759666037803	10.846425853216981	nuclear_charge=	0.66666666666666663
H	15.668044098558941	5.6548184329490496	9.1242303731047247	nuclear_charge=	0.66666666666666663
H	9.7673090800640239E-002	12.232630912018177	10.818801201452073	nuclear_charge=	0.66666666666666663
H	0.81661009896243009	12.240525887656602	9.0980809954317099	nuclear_charge=	0.66666666666666663
H	16.380487466105233	12.237349728300643	10.822984723288753	nuclear_charge=	0.66666666666666663
H	15.666610759159635	12.240525887652240	9.0980809958215278	nuclear_charge=	0.66666666666666663
H	3.0720802431568615	2.3909869447653538	1.7529738763723044	nuclear_charge=	0.66666666666666663
H	4.4976089172395497	2.3909869447653556	1.7529739291690738	nuclear_charge=	0.66666666666666663
H	6.0420802671029463	2.3909869447653556	1.7529739560474258	nuclear_charge=	0.66666666666666663
H	7.4676089414267466	2.3909869447653520	1.7529738497339338	nuclear_charge=	0.66666666666666663
H	9.0120810471286674	2.3909869447653662	1.7529742745078412	nuclear_charge=	0.66666666666666663
H	10.437610361401170	2.3909869447653627	1.7529741681943949	nuclear_charge=	0.66666666666666663
H	11.982080955426852	2.3909869447653413	1.7529734779965636	nuclear_charge=	0.66666666666666663
H	13.407608989318817	2.3909869447653467	1.7529736903836581	nuclear_charge=	0.66666666666666663
H	3.0720802431568650	8.9840517977288989	1.7529738763723071	nuclear_charge=	0.66666666666666663
H	4.4976089172395461	8.9840517977289061	1.7529739291690767	nuclear_charge=	0.66666666666666663
H	6.0420802671029463	8.9840517977289096	1.7529739560474287	nuclear_charge=	0.66666666666666663
H	7.4676089414267466	8.9840517977288954	1.7529738497339364	nuclear_charge=	0.66666666666666663
H	9.0120810471286710	8.9840517977289522	1.7529742745078440	nuclear_charge=	0.66666666666666663
H	10.437610361401166	8.9840517977289380	1.7529741681943978	nuclear_charge=	0.66666666666666663
H	11.982080955426856	8.9840517977288457	1.7529734779965664	nuclear_charge=	0.66666666666666663
H	13.407608989318813	8.9840517977288741	1.7529736903836612	nuclear_charge=	0.66666666666666663
H	8.7663244390052242E-002	5.6875191067300150	3.9822328092337882	nuclear_charge=	0.66666666666666663
H	0.81484469437964080	5.6875191318615386	2.2703232550000529	nuclear_charge=	0.66666666666666663
H	16.392025086725447	5.6875191067301039	3.9822331339295034	nuclear_charge=	0.66666666666666663
H	15.664844685192229	5.6875191318615386	2.2703232550001422	nuclear_charge=	0.66666666666666663
H	3.7848446145738315	5.6875191318615386	2.2703232550000250	nuclear_charge=	0.66666666666666663
H	6.7548445347680222	5.6875191318615386	2.2703232550000307	nuclear_charge=	0.66666666666666663
H	9.7248456347680232	5.6875191318615386	2.2703232550000307	nuclear_charge=	0.66666666666666663
H	12.694845110379642	5.6875191318615386	2.2703232550000529	nuclear_charge=	0.66666666666666663
H	8.7663244390052242E-002	12.280582704730016	3.9822328092337882	nuclear_charge=	0.66666666666666663
H	0.81484469437964080	12.280582729861539	2.2703232550000529	nuclear_charge=	0.66666666666666663
H	16.392025086725447	12.280582704730104	3.9822331339295034	nuclear_charge=	0.66666666666666663
H	15.664844685192229	12.280582729861539	2.2703232550001422	nuclear_charge=	0.66666666666666663
H	3.7848446145738315	12.280582729861539	2.2703232550000250	nuclear_charge=	0.66666666666666663
H	6.7548445347680222	12.280582729861539	2.2703232550000307	nuclear_charge=	0.66666666666666663
H	9.7248456347680232	12.280582729861539	2.2703232550000307	nuclear_charge=	0.66666666666666663
H	12.694845110379642	12.280582729861539	2.2703232550000529	nuclear_charge=	0.66666666666666663
H	0.10068038044395244	2.3552313847683219	8.5388417758844621	nuclear_charge=	0.66666666666666663
H	0.82788424331135957	2.3449665513479339	10.249298403394219	nuclear_charge=	0.66666666666666663
H	16.411733770555099	2.3533106028708328	8.5430008829547344	nuclear_charge=	0.66666666666666663

H	15.677883522290088	2.3449665513491382	10.249298402782328	nuclear_charge= 0.66666666666666663
H	9.1060614461619949E-002	8.9629255318970671	8.5440813363149708	nuclear_charge= 0.66666666666666663
H	0.82158713207809875	8.9509970558941383	10.253017191320810	nuclear_charge= 0.66666666666666663
H	16.398724752872614	8.9641354624106420	8.5418658788172586	nuclear_charge= 0.66666666666666663
H	15.671586406564668	8.9509970558892746	10.253017191616584	nuclear_charge= 0.66666666666666663
H	6.3651152794390597E-002	5.6872386070840868	5.3299598787511151	nuclear_charge= 0.66666666666666663
H	0.82894383084829748	5.6754116532086769	6.9903039620202225	nuclear_charge= 0.66666666666666663
H	16.450972618125487	5.6872438051208789	5.3185404874565823	nuclear_charge= 0.66666666666666663
H	15.678943161751928	5.6754116532473091	6.9903039586353914	nuclear_charge= 0.66666666666666663
H	4.2158069398663400E-002	12.275177267991218	5.3124911500568563	nuclear_charge= 0.66666666666666663
H	0.81612268823036871	12.260910567594838	6.9732657807703156	nuclear_charge= 0.66666666666666663
H	16.438338405967514	12.275179655386776	5.3122265173477903	nuclear_charge= 0.66666666666666663
H	15.666123251057716	12.260910567599968	6.9732657801532039	nuclear_charge= 0.66666666666666663
H	4.8943006780817200E-002	2.3651081860429226	7.2255842476490297	nuclear_charge= 0.66666666666666663
H	0.81726594026834931	2.3775054716472379	5.5613538107779377	nuclear_charge= 0.66666666666666663
H	16.443186231272275	2.3631099008663501	7.2212140842572108	nuclear_charge= 0.66666666666666663
H	15.667265884330433	2.3775054716513768	5.5613538103942188	nuclear_charge= 0.66666666666666663
H	4.4178456519254894E-002	8.9600355174905886	7.2226525885611919	nuclear_charge= 0.66666666666666663
H	0.81708841019873901	8.9719505508885611	5.5636788284834058	nuclear_charge= 0.66666666666666663
H	16.444333178835670	8.9613222744033685	7.2218024998593879	nuclear_charge= 0.66666666666666663
H	15.667088353505282	8.9719505508919966	5.5636788281280394	nuclear_charge= 0.66666666666666663

Table S5. Cartesian coordinate of cluster Ag_{37}-TiO_2 z

Ti	2.9611419328818371	5.2712851236899425	8.9072525776076663
Ti	2.9608485118818351	11.871647486689941	8.9075454746076659
Ti	5.9135670268818359	5.2759772716899427	8.9009007486076666
Ti	5.9129523048818342	11.866552143689940	8.8995551356076668
Ti	8.8944167098818347	5.2691465416899419	8.8761564346076653
Ti	8.8942241938818363	11.874493427689941	8.8750841116076664
Ti	11.898502354881835	5.2677011706899428	8.8922118676076654
Ti	11.898531564881836	11.874311872689940	8.8915231296076662
Ti	14.853605406881837	5.2676398666899402	8.9047206696076664
Ti	14.853289415881836	11.875099398689940	8.9048861136076667
Ti	2.9673103958818352	5.2718156426899405	2.0399487386076665
Ti	2.9669081048818349	11.870841097689940	2.0398139316076662
Ti	5.9353624418818356	5.2771078666899420	2.0354504966076661
Ti	5.9351181468818375	11.865616072689942	2.0351337016076663
Ti	8.9091607448818344	5.2791438826899402	2.0375479596076662
Ti	8.9090956788818375	11.863630751689943	2.0372777346076658
Ti	11.880461656881835	5.2749409906899416	2.0340901776076663
Ti	11.880435102881837	11.868094186689941	2.0338168886076664
Ti	14.851000472881836	5.2698692266899414	2.0391527686076660
Ti	14.850838493881835	11.872930164689940	2.0394885586076663
Ti	2.9301933988818369	1.9782624146899419	5.3382649106076663
Ti	2.9794402038818362	8.5711031946899396	5.3285968466076667
Ti	5.9249214608818370	1.9782164366899408	5.3440125626076664
Ti	5.9733291638818358	8.5714639476899421	5.3188209366076666
Ti	8.9175092818818342	1.9781669216899420	5.3412588366076665
Ti	8.9021810608818370	8.5711303096899414	5.3446081616076668
Ti	11.914352359881835	1.9781598476899411	5.3384364826076656
Ti	11.849675100881836	8.5711161626899397	5.2973757016076668
Ti	14.846303092881836	1.9780419546899406	5.3823196176076653
Ti	14.842930751881838	8.5711833616899398	5.3289228326076667
Ti	4.4524935038818363	5.2747122776899431	5.4699449556076658
Ti	4.4525598888818365	11.868103618689940	5.4692317076076655
Ti	7.4252429288818362	5.2704362926899400	5.4590048096076664
Ti	7.4252190308818342	11.872038892689943	5.4579753786076655
Ti	10.391134817881834	5.2719299986899415	5.4652573716076667
Ti	10.391509227881837	11.870334156689943	5.4647316266076658
Ti	13.362297649881835	5.2757638846899404	5.4648308936076653
Ti	13.362470249881834	11.866488481689942	5.4649191306076652
Ti	4.4539128078818351	1.9781999316899412	8.6001600906076661
Ti	4.4355773618818368	8.5719072256899409	8.5687122266076656
Ti	7.4170191958818350	1.9787186616899408	8.5985093266076653
Ti	7.3907189168818377	8.5704712866899406	8.5874061866076659
Ti	10.385462911881834	1.9784451496899411	8.5982372626076664
Ti	10.450737631881836	8.5696024136899425	8.5458993196076651

Ti	13.353776512881836	1.9780608176899417	8.6276140116076654
Ti	13.388360271881837	8.5717421756899412	8.5608027716076656
Ti	4.4531533678818356	1.9783508346899410	2.0591059426076663
Ti	4.4534720138818358	8.5713495906899411	2.0507975836076664
Ti	7.4237638788818359	1.9784368966899422	2.0594405076076656
Ti	7.4210142258818372	8.5714545156899398	2.0542890676076659
Ti	10.394061053881835	1.9784710856899412	2.0592830286076662
Ti	10.396437625881838	8.5713743486899396	2.0526799706076657
Ti	13.360380459881835	1.9784616546899407	2.0695479176076663
Ti	13.365481457881835	8.5714745576899425	2.0464127016076663
O	2.9727725268818368	6.5887542786899402	5.4161535546076660
O	2.9704092328818348	1.3681825310058571E-002	5.4230200986076653
O	2.9704092328818348	13.172445369689942	5.4230200986076653
O	5.9427935418818372	6.5773481146899400	5.4230066186076655
O	5.9382568138818357	3.4109723100588241E-003	5.4214355116076653
O	5.9382568138818357	13.182716222689940	5.4214355116076653
O	8.9048364478818343	6.5521519866899425	5.4256953916076665
O	8.9068093998818370	2.1005336899406757E-003	5.4218190966076651
O	8.9068093998818370	13.188226549689940	5.4218190966076651
O	11.871637805881836	6.5805559876899409	5.4158569806076660
O	11.874481725881836	1.0384353310058714E-002	5.4248718476076654
O	11.874481725881836	13.175744020689940	5.4248718476076654
O	14.846334957881837	6.5937494126899416	5.4173410756076663
O	14.845841055881834	3.8487727310059228E-002	5.4250005266076666
O	14.845841055881834	13.147640645689940	5.4250005266076666
O	2.9704530468818362	3.9701076246899412	5.4229551466076664
O	2.9727897868818367	10.553783388689940	5.4160677686076664
O	5.9381784798818344	3.9598320566899421	5.4212100176076667
O	5.9427391068818345	10.565426518689943	5.4226058756076654
O	8.9067310658818357	3.9541932256899415	5.4216573296076653
O	8.9048351198818345	10.590363282689943	5.4250495466076654
O	11.874495002881837	3.9666639656899410	5.4245470866076655
O	11.871637805881836	10.561849639689942	5.4155003566076658
O	14.845795913881837	3.9948144966899406	5.4247309136076662
O	14.846494280881835	10.548622025689941	5.4172969576076664
O	2.9628891768818342	7.3324762756899418	8.9173606006076653
O	2.9836237658818376	0.74048079068994177	8.9545316066076666
O	2.9836237658818376	13.926607984689941	8.9545316066076666
O	5.9154868708818356	7.3330339106899416	8.9203238886076655
O	5.9474298478818355	0.73733186368994197	8.9550303896076660
O	5.9474298478818355	13.923457879689941	8.9550303896076660
O	8.9242884838818348	7.3322900046899413	8.8898355996076663
O	8.8962263558818364	0.73656555868994111	8.9649080166076658
O	8.8962263558818364	13.922693931689942	8.9649080166076658
O	11.908776046881837	7.3255665566899424	8.9039534986076667
O	11.853243940881836	0.73906489368994066	8.9667732466076657
O	11.853243940881836	13.925193266689941	8.9667732466076657
O	14.856019153881835	7.3315001206899417	8.9136705836076668
O	14.843111317881835	0.74016129968994093	8.9397127146076656
O	14.843111317881835	13.926289673689940	8.9397127146076656
O	2.9837525518818353	3.2162385636899415	8.9543367506076663
O	2.9625413218818366	9.8105070286899405	8.9176081536076666
O	5.9472944228818356	3.2192978906899405	8.9561676656076656
O	5.9161573568818362	9.8098586166899402	8.9200775606076661
O	8.8963684188818348	3.2203200246899417	8.9660134286076651
O	8.9247027248818362	9.8096864926899414	8.8934042906076662
O	11.853291737881836	3.2172276866899416	8.9679963076076668
O	11.907841350881835	9.8163380246899408	8.9041446786076666
O	14.842981203881838	3.2164625606899406	8.9396759496076665
O	14.856231584881836	9.8111955246899427	8.9139046566076665
O	2.9672108188818349	7.2946537886899421	2.0374315366076656
O	2.9797495558818348	0.69967206968994056	2.0258271626076656
O	2.9797495558818348	13.885800442689941	2.0258271626076656
O	5.9277056338818355	7.2913563166899422	2.0174072826076657
O	5.9412042928818352	0.70204407968994076	2.0448532376076658
O	5.9412042928818352	13.888170095689940	2.0448532376076658
O	8.9074931618818347	7.2934430256899425	2.0318720006076658
O	8.9055773008818342	0.70169865268994158	2.0470315846076659

O	8.9055773008818342	13.887825847689943	2.0470315846076659
O	11.890273310881835	7.2918267106899428	2.0164048136076662
O	11.867179411881835	0.69931367368994124	2.0243847356076659
O	11.867179411881835	13.885442047689942	2.0243847356076659
O	14.852856587881835	7.2941279846899398	2.0372728326076661
O	14.848554860881837	0.70165856968994156	2.0399756996076661
O	14.848554860881837	13.887785763689941	2.0399756996076661
O	2.9798093018818363	3.2568975596899410	2.0259288806076663
O	2.9672055088818361	9.8481196656899428	2.0375896276076659
O	5.9411737558818345	3.2545090446899412	2.0449813036076661
O	5.9276631478818373	9.8515008426899406	2.0175574076076659
O	8.9055427808818344	3.2549169546899410	2.0472190886076662
O	8.9075396318818356	9.8494400696899405	2.0319449186076657
O	11.867171445881837	3.2574811316899410	2.0245342476076660
O	11.890225514881838	9.8511070786899424	2.0165604536076662
O	14.848626556881836	3.2550572476899422	2.0401344036076656
O	14.852875175881834	9.8486997006899415	2.0374137666076662
O	1.4813253618818365	5.2768296396899430	10.066927669607667
O	16.331325549881836	5.2768296396899430	10.066927669607667
O	1.4811846258818360	11.865972109689942	10.067458316607667
O	16.331184814881837	11.865972109689942	10.067458316607667
O	4.4588491728818376	5.2906938766899430	10.076737893607666
O	4.4590589478818359	11.852072504689943	10.076525879607667
O	7.4204924418818372	5.3670745036899419	10.132448445607665
O	7.4201645018818354	11.778792467689943	10.132061184607664
O	10.378442068881835	5.3044272516899404	10.124106385607664
O	10.378149975881836	11.840896232689943	10.123219114607664
O	13.352677182881838	5.2819603496899425	10.070773326607666
O	13.352231077881836	11.860454709689943	10.070503713607666
O	1.4853031308818352	5.2733682956899415	7.4556666416076656
O	16.335303319881834	5.2733682956899415	7.4556666416076656
O	1.4853097698818356	11.869310844689942	7.4560208146076654
O	16.335311285881836	11.869310844689942	7.4560208146076654
O	4.4626238048818365	5.2702240846899429	7.4599032366076656
O	4.4623184358818371	11.872335982689940	7.4597377926076653
O	7.4287878708818376	5.2564635936899400	7.4814158756076665
O	7.4290919128818373	11.885273579689940	7.4810911146076666
O	10.378938625881837	5.2613349396899416	7.4818693146076658
O	10.378481899881837	11.880230109689940	7.4815077886076669
O	13.348221443881837	5.2665281336899419	7.4603052046076659
O	13.348303760881837	11.875446004689941	7.4602868216076654
O	1.4844029548818352	5.2743809976899421	0.72913124460766632
O	16.334403142881836	5.2743809976899421	0.72913124460766632
O	1.4843591408818355	11.868438434689942	0.72916126960766636
O	16.334360656881834	11.868438434689942	0.72916126960766636
O	4.4544226428818376	5.2727198836899412	0.72826787160766582
O	4.4543376698818342	11.870114875689943	0.72827828760766611
O	7.4241289948818370	5.2711766616899425	0.73112147560766605
O	7.4240612818818370	11.871645128689941	0.73109145060766600
O	10.390067351881836	5.2753288596899424	0.72948603060766626
O	10.390032831881836	11.867511794689943	0.72929485060766641
O	13.366511748881837	5.2711342196899409	0.72838000560766591
O	13.366522369881835	11.871680496689940	0.72825622860766615
O	1.4860678828818354	5.2747429296899426	3.3723628176076659
O	16.336068070881836	5.2747429296899426	3.3723628176076659
O	1.4860479668818360	11.868058818689942	3.3723928436076669
O	16.336046827881837	11.868058818689942	3.3723928436076669
O	4.4553201628818364	5.2755646456899399	3.3754345636076657
O	4.4552803328818342	11.867351459689942	3.3754370146076660
O	7.4214231548818361	5.2750011166899426	3.3784860886076657
O	7.4214005848818374	11.867806527689943	3.3783604736076658
O	10.393713198881837	5.2745979216899421	3.3761269786076653
O	10.393668056881836	11.868240374689940	3.3759094496076667
O	13.360476053881836	5.2754656156899422	3.3727733646076654
O	13.360414979881835	11.867422195689940	3.3725821846076656
O	1.5416570788818351	1.9782576996899408	6.7661751786076660
O	16.391655939881836	1.9782576996899408	6.7661751786076660
O	1.4863719248818352	8.5712647076899415	6.6984644126076667

O 16.336373440881836 8.5712647076899415 6.6984644126076667
O 4.4635292918818372 1.9783366876899411 6.7215089416076665
O 4.4456372948818377 8.5714887046899406 6.6948283186076658
O 7.4220777078818365 1.9784569386899413 6.7192638036076655
O 7.3679184368818369 8.5717822586899430 6.7315654936076665
O 10.379735241881836 1.9783425826899421 6.7229673006076656
O 10.452492842881835 8.5715205356899418 6.7001507176076665
O 13.300371371881837 1.9782518046899416 6.7690061116076663
O 13.371105567881838 8.5713967486899421 6.6875095606076655
O 4.4592023388818376 1.9784592966899410 5.3340226076663555E-003
O 4.4510343688818352 8.5715476516899400 2.2720816076660100E-003
O 7.4248964008818348 1.9784616546899407 8.5883706076659294E-003
O 7.4233230848818366 8.5715700516899425 3.5693223923338735E-003
O 10.390038142881835 1.9784805176899418 5.4430936076661496E-003
O 10.396190674881836 8.5715452936899403 3.9375883923336019E-003
O 13.362932286881836 1.9784899486899405 5.9460766621555194E-007
O 13.368869731881837 8.5715606196899401 1.5000086076657482E-003
O 1.5408259428818365 1.9781999316899412 4.0411504796076656
O 16.390824803881834 1.9781999316899412 4.0411504796076656
O 1.4870596698818357 8.5712293396899426 4.0775083586076661
O 16.337058529881837 8.5712293396899426 4.0775083586076661
O 4.4539871588818372 1.9781103326899405 4.0898002446076660
O 4.4524616398818360 8.5712882866899420 4.0735952986076658
O 7.4208217098818352 1.9780749646899416 4.0918670706076661
O 7.3594889118818365 8.5712941806899430 4.0189049856076657
O 10.391639340881834 1.9780749646899416 4.0893253586076668
O 10.447612241881835 8.5711562456899415 4.0143656886076666
O 13.303342749881836 1.9780631756899414 4.0404200746076668
O 13.372932473881836 8.5712364136899417 4.0663372036076666
Ag 9.0708419418818345 8.5900462686899424 19.444163523607667
Ag 9.0956007688818374 8.5909446146899420 22.289152984607664
Ag 9.0215314068818344 8.5883556806899399 16.598176495607667
Ag 9.0971063728818358 8.5881081056899404 25.153775148607664
Ag 8.9057499008818368 8.5872993576899397 13.664727124607666
Ag 9.0910096048818367 8.5885065836899415 28.010103047607664
Ag 9.8803897388818349 10.929942772689941 23.642544767607667
Ag 7.0783551368818358 10.025588972689942 23.674160526607665
Ag 7.0866027678818355 7.1374845076899405 23.674167880607666
Ag 9.8862846978818375 6.2497819386899423 23.640782480607665
Ag 11.558809332881836 8.5930997016899404 18.152515489607666
Ag 9.8891604818818344 10.911063359689940 17.951894264607667
Ag 7.0549014318818344 10.029340332689941 18.017843970607665
Ag 7.0590756998818343 7.1412040366899419 18.020576861607665
Ag 9.8932657098818346 6.2693698896899406 17.950553554607666
Ag 11.585264952881836 8.5921093996899423 23.717649047607665
Ag 9.8659391238818372 10.891120550689941 26.476583052607666
Ag 7.1215131398818343 10.004674723689941 26.521228456607666
Ag 7.1204523128818344 7.1708518116899427 26.521726014607665
Ag 9.8576596278818371 6.2805084356899421 26.474791353607664
Ag 11.538535454881835 8.5905319886899427 15.380095950607664
Ag 9.8543111848818370 10.847608539689940 15.027459759607666
Ag 7.0472207258818358 10.048391869689940 15.158501312607665
Ag 7.0521451408818372 7.1211822396899400 15.160565074607668
Ag 9.8587430258818358 6.3304456256899400 15.023545473607665
Ag 11.528232553881836 8.5846750556899423 26.565511108607666
Ag 9.8996917468818353 10.927893788689943 20.832624558607666
Ag 7.0769995618818342 10.023499905689942 20.873853235607665
Ag 7.0816053298818353 7.1503643366899396 20.875703758607667
Ag 9.9006662738818356 6.2528695606899412 20.832403966607664
Ag 11.582904313881837 8.5939508906899427 20.936994067607667
Ag 9.1116207178818343 8.5914114716899412 11.041073718607667
Ag 11.522411945881835 8.5845430156899418 12.594344065607665
Ag 10.152896162881834 10.977276880689942 12.186942192607667
Ag 7.3249184298818371 10.437396892689943 12.122255989607666
Ag 7.3272870348818344 6.7374140306899406 12.114217855607667
Ag 10.148963534881837 6.1969198196899420 12.175415025607666
H 2.2143538343399491 5.9218706808022858 5.4473867688430033 nuclear_charge= 0.66666666666666663
H 2.2105353169862632 -0.67905277287937160 5.4509717367035293 nuclear_charge= 0.66666666666666663

H	3.7287100054271249	-0.68101166350322551	5.4466629709859919	nuclear_charge= 0.66666666666666663
H	2.9500319648296021	14.181726675347047	5.3800762632198396	nuclear_charge= 0.66666666666666663
H	2.2105348707661303	12.507074932407512	5.4509717531175479	nuclear_charge= 0.66666666666666663
H	5.1817767282720659	-0.67277915803044408	5.4457721510887644	nuclear_charge= 0.66666666666666663
H	6.6961050624847012	-0.67141349965603858	5.4400584960342648	nuclear_charge= 0.66666666666666663
H	5.9314626728860134	14.192323094605914	5.3819898042598062	nuclear_charge= 0.66666666666666663
H	8.1515551774329573	-0.66883874664569909	5.4402500915843568	nuclear_charge= 0.66666666666666663
H	9.6622791390723037	-0.66849386215025142	5.4436546051938581	nuclear_charge= 0.66666666666666663
H	8.9122758239029203	14.197773164781839	5.3806619567056710	nuclear_charge= 0.66666666666666663
H	11.116214459018973	-0.67785984423985290	5.4452527458301105	nuclear_charge= 0.66666666666666663
H	12.632892092634691	-0.67769630500094458	5.4452834798868164	nuclear_charge= 0.66666666666666663
H	11.894717239735215	14.184988411621674	5.3810033697229294	nuclear_charge= 0.66666666666666663
H	15.601209251700148	5.9228317168841151	5.4480397072858029	nuclear_charge= 0.66666666666666663
H	15.610360451950505	-0.69854527863221350	5.4522453789176453	nuclear_charge= 0.66666666666666663
H	14.081320573236674	-0.69878562214445950	5.4455743374667964	nuclear_charge= 0.66666666666666663
H	14.846072511755636	14.157814375984106	5.4036196694745673	nuclear_charge= 0.66666666666666663
H	15.610360602241336	12.487583268665009	5.4522453842734961	nuclear_charge= 0.66666666666666663
H	2.2105538741931330	4.6354528797646175	5.4508316402292998	nuclear_charge= 0.66666666666666663
H	2.2143388655375666	11.220623177101306	5.4474533784234858	nuclear_charge= 0.66666666666666663
H	15.610335938234705	4.6548470227730494	5.4520031646124423	nuclear_charge= 0.66666666666666663
H	15.601297261035359	11.219613849476406	5.4481286549834600	nuclear_charge= 0.66666666666666663
H	2.2002104379907514	7.9718062585155174	8.7427568368917665	nuclear_charge= 0.66666666666666663
H	2.9724290599946421	-0.26959319502593715	8.9314365434958827	nuclear_charge= 0.66666666666666663
H	2.2162997363647481	1.3758572165480896	8.7859528677661984	nuclear_charge= 0.66666666666666663
H	2.2162997366098125	14.561984410858333	8.7859528678200380	nuclear_charge= 0.66666666666666663
H	3.7437842207213095	14.566527085314824	8.7713164665299814	nuclear_charge= 0.66666666666666663
H	5.9305028740771562	-0.27255911271129385	8.9277934048044028	nuclear_charge= 0.66666666666666663
H	5.1829287138445288	14.558634326760043	8.7733794684895123	nuclear_charge= 0.66666666666666663
H	6.7063775808204724	14.564555569259269	8.7709101813301089	nuclear_charge= 0.66666666666666663
H	8.8952402700022866	-0.27286372574063122	8.9206394344232098	nuclear_charge= 0.66666666666666663
H	8.1360157423872828	14.561074512104902	8.7766044912044201	nuclear_charge= 0.66666666666666663
H	9.6587079998858520	14.558528917235936	8.7771747651239238	nuclear_charge= 0.66666666666666663
H	11.875535206059492	-0.27040989665155024	8.9297339814720829	nuclear_charge= 0.66666666666666663
H	11.095073521449923	14.565386272100138	8.7764086203708214	nuclear_charge= 0.66666666666666663
H	12.620799319165442	14.558966903897748	8.7932858463496171	nuclear_charge= 0.66666666666666663
H	15.619022073502908	7.9709859281403013	8.7410652339823365	nuclear_charge= 0.66666666666666663
H	14.848124183732324	-0.27008066078133552	8.9225600915103556	nuclear_charge= 0.66666666666666663
H	15.611294841203545	1.3764242927618291	8.7785555300199221	nuclear_charge= 0.66666666666666663
H	15.611295131192875	14.562552301237769	8.7785554691830736	nuclear_charge= 0.66666666666666663
H	14.075974693963811	14.563915312868009	8.7789548080947544	nuclear_charge= 0.66666666666666663
H	2.2162575810790681	2.5810482103591230	8.7858349855488740	nuclear_charge= 0.66666666666666663
H	2.2000992142966531	9.1709261838747658	8.7428897071671958	nuclear_charge= 0.66666666666666663
H	15.611349487110665	2.5804241816941555	8.7785129210546042	nuclear_charge= 0.66666666666666663
H	15.619100031968468	9.1715803779155607	8.7411841990572814	nuclear_charge= 0.66666666666666663
H	2.2014661819819032	7.9538107365533186	2.0448766116529651	nuclear_charge= 0.66666666666666663
H	2.9733098394912183	-0.31068306740428042	2.0328405070399267	nuclear_charge= 0.66666666666666663
H	2.2122237023969529	1.3564009241394075	2.0486183004860878	nuclear_charge= 0.66666666666666663
H	2.2122237023969529	14.542529297139410	2.0486183004860878	nuclear_charge= 0.66666666666666663
H	3.7427434878476546	14.547957097536127	2.0430603928982531	nuclear_charge= 0.66666666666666663
H	5.9381632461678997	-0.30833967731579648	2.0399977650399350	nuclear_charge= 0.66666666666666663
H	5.1742848592642758	14.545960931754834	2.0521988709206642	nuclear_charge= 0.66666666666666663
H	6.7068970027332178	14.547386354090420	2.0523870773272339	nuclear_charge= 0.66666666666666663
H	8.9073335212317453	-0.30868809089837868	2.0421629019243879	nuclear_charge= 0.66666666666666663
H	8.1401307724213865	14.547339823630566	2.0534415461394371	nuclear_charge= 0.66666666666666663
H	9.6724782910102043	14.545648914444353	2.0533438097261234	nuclear_charge= 0.66666666666666663
H	11.873818387124892	-0.31105347139655137	2.0291087315318102	nuclear_charge= 0.66666666666666663
H	11.104382750541788	14.547803627737519	2.0424554501855550	nuclear_charge= 0.66666666666666663
H	12.634317454362009	14.542609465790807	2.0475875019585610	nuclear_charge= 0.66666666666666663
H	15.618046389808434	7.9539279598488832	2.0448040880001379	nuclear_charge= 0.66666666666666663
H	14.849700044627792	-0.30874075180486926	2.0397314108120543	nuclear_charge= 0.66666666666666663
H	15.615076994650423	1.3597689078819357	2.0555564957258596	nuclear_charge= 0.66666666666666663
H	15.615076694323506	14.545896451826653	2.0555564896212317	nuclear_charge= 0.66666666666666663
H	14.081801478670322	14.545634727619326	2.0552122190342601	nuclear_charge= 0.66666666666666663
H	2.2122827290369731	2.6001677041544138	2.0486668845945095	nuclear_charge= 0.66666666666666663
H	2.2014847576237422	9.1889340517254894	2.0449528765540563	nuclear_charge= 0.66666666666666663
H	15.615083502953343	2.5968690458144721	2.0556328141931770	nuclear_charge= 0.66666666666666663
H	15.618024246228721	9.1888516604566988	2.0448719130019928	nuclear_charge= 0.66666666666666663
H	0.68713684542302822	5.2718906719022733	9.4423090924566591	nuclear_charge= 0.66666666666666663

H	17.126610922193660	5.2738498997676651	9.4436937987880203	nuclear_charge= 0.66666666666666663
H	0.68705534679616242	11.870876548423144	9.4427641557740785	nuclear_charge= 0.66666666666666663
H	17.126376489585279	11.869022135161202	9.4441052402320409	nuclear_charge= 0.66666666666666663
H	0.76293205933623298	5.2705755189967363	8.1621228648744726	nuclear_charge= 0.66666666666666663
H	1.4822905530218193	5.2745209324387474	6.4452717901742460	nuclear_charge= 0.66666666666666663
H	17.055658344337161	5.2723515030255363	8.1641832362437015	nuclear_charge= 0.66666666666666663
H	16.332290741511009	5.2745209324387474	6.4452717901757683	nuclear_charge= 0.66666666666666663
H	0.76281588502652120	11.872132799412316	8.1623513200498561	nuclear_charge= 0.66666666666666663
H	1.4822682446947670	11.868043876619257	6.4456261867931453	nuclear_charge= 0.66666666666666663
H	17.055608505758851	11.870451498067712	8.1645959863059936	nuclear_charge= 0.66666666666666663
H	16.332269082389320	11.868043876621819	6.4456261888352273	nuclear_charge= 0.66666666666666663
H	0.72705735908908409	5.2720775260292143	1.3979589522810478	nuclear_charge= 0.66666666666666663
H	1.4846844734344451	5.2746959674856519	-0.28126866708142906	nuclear_charge= 0.66666666666666663
H	17.091437892516893	5.2730713646242897	1.3983134564948472	nuclear_charge= 0.66666666666666663
H	16.334684661905637	5.2746959674856519	-0.28126866708129761	nuclear_charge= 0.66666666666666663
H	0.72706394811220676	11.870731335103585	1.3980460830215335	nuclear_charge= 0.66666666666666663
H	1.4846612999634701	11.868285118438003	-0.28123867358018639	nuclear_charge= 0.66666666666666663
H	17.091356959295286	11.869665246538379	1.3983871306329645	nuclear_charge= 0.66666666666666663
H	16.334662190702169	11.868285118437978	-0.28123867376697653	nuclear_charge= 0.66666666666666663
H	4.4546948866656138	5.2738179965313954	-0.28213149499521784	nuclear_charge= 0.66666666666666663
H	4.4546499667875672	11.869171258879536	-0.28212122350561497	nuclear_charge= 0.66666666666666663
H	7.4245390047755393	5.2729997898329941	-0.27927679640969139	nuclear_charge= 0.66666666666666663
H	7.4245031741729406	11.869982368063916	-0.27930708460420917	nuclear_charge= 0.66666666666666663
H	10.392391679269430	5.2751972278198060	-0.28091128736939908	nuclear_charge= 0.66666666666666663
H	10.392373630540042	11.867795078108891	-0.28110239820695071	nuclear_charge= 0.66666666666666663
H	13.365799571079659	5.2729796843173311	-0.28201805805986080	nuclear_charge= 0.66666666666666663
H	13.365805144203428	11.869998865158955	-0.28214211744339490	nuclear_charge= 0.66666666666666663
H	0.73420336349557935	5.2722754521691790	2.6973799911277929	nuclear_charge= 0.66666666666666663
H	1.4828704049264481	5.2751661527848093	4.3827576696417285	nuclear_charge= 0.66666666666666663
H	17.087268912691609	5.2732583770867585	2.6966385755030498	nuclear_charge= 0.66666666666666663
H	16.332870592926447	5.2751661527848093	4.3827576696417285	nuclear_charge= 0.66666666666666663
H	0.73407379883517443	11.870525222223264	2.6975321709913551	nuclear_charge= 0.66666666666666663
H	1.4828361155361289	11.867469300249351	4.3827875667129206	nuclear_charge= 0.66666666666666663
H	17.087120031217029	11.869469953525360	2.6965265758221761	nuclear_charge= 0.66666666666666663
H	16.332835613342656	11.867469300248167	4.3827875687370046	nuclear_charge= 0.66666666666666663
H	0.78894857353182957	1.9781526149791215	6.0921291453644137	nuclear_charge= 0.66666666666666663
H	1.5127118438189999	1.9784574463021833	7.7761604722968602	nuclear_charge= 0.66666666666666663
H	17.096059633399822	1.9782606897128261	6.0417971029115005	nuclear_charge= 0.66666666666666663
H	16.362710704276402	1.9784574463021816	7.7761604722813100	nuclear_charge= 0.66666666666666663
H	0.74168891515369317	8.5712241456794800	6.0155622429106028	nuclear_charge= 0.66666666666666663
H	1.4857263530664842	8.5712311370292795	7.7088642058133274	nuclear_charge= 0.66666666666666663
H	17.080890471875161	8.5711835814839183	6.0153812961573898	nuclear_charge= 0.66666666666666663
H	16.335727155563305	8.5712311370292937	7.7088642053571981	nuclear_charge= 0.66666666666666663
H	3.6836767581299981	1.9784899942751615	-0.64232964987765762	nuclear_charge= 0.66666666666666663
H	5.2329206421729602	1.9784900965566052	-0.64448760384352877	nuclear_charge= 0.66666666666666663
H	3.6764769548192682	8.5715655319967041	-0.64654913567873340	nuclear_charge= 0.66666666666666663
H	5.2272942042301374	8.5715654758395523	-0.64451136569701628	nuclear_charge= 0.66666666666666663
H	6.6511298719338967	1.9784911427244527	-0.64117583109193355	nuclear_charge= 0.66666666666666663
H	8.1987075580472109	1.9784911403124159	-0.64112268222811686	nuclear_charge= 0.66666666666666663
H	6.6467698517851517	8.5715762234987807	-0.65000047582554643	nuclear_charge= 0.66666666666666663
H	8.2005935420362377	8.5715762152633879	-0.64913790619811085	nuclear_charge= 0.66666666666666663
H	9.6165121185265363	1.9785002347762450	-0.64460740385873816	nuclear_charge= 0.66666666666666663
H	11.165698214777461	1.9785001574942758	-0.64205950289766722	nuclear_charge= 0.66666666666666663
H	9.6189293001481708	8.5715644073669637	-0.64951710710540311	nuclear_charge= 0.66666666666666663
H	11.172942784882675	8.5715644255057057	-0.65012975736625478	nuclear_charge= 0.66666666666666663
H	12.587381568282865	1.9785047465348278	-0.64763297668218289	nuclear_charge= 0.66666666666666663
H	14.139369156729412	1.9785047222540655	-0.64657031946366850	nuclear_charge= 0.66666666666666663
H	12.592432342943290	8.5715716863220770	-0.64507028218508644	nuclear_charge= 0.66666666666666663
H	14.143646529206535	8.5715717203645525	-0.64705922091887391	nuclear_charge= 0.66666666666666663
H	0.77790938183399838	1.9781218990115033	4.7036204599009945	nuclear_charge= 0.66666666666666663
H	1.5124498308512209	1.9782301387068273	3.0311490165624075	nuclear_charge= 0.66666666666666663
H	17.129384539703267	1.9782337731260089	4.7306703672672832	nuclear_charge= 0.66666666666666663
H	16.362449371327649	1.9782301387073993	3.0311489974726644	nuclear_charge= 0.66666666666666663
H	0.71245932435627957	8.5712055033463841	4.7262783210315717	nuclear_charge= 0.66666666666666663
H	1.4855729088116778	8.5712852048616526	3.0671094540058235	nuclear_charge= 0.66666666666666663
H	17.111368585019687	8.5711632787384922	4.7266247535233781	nuclear_charge= 0.66666666666666663
H	16.335572431217482	8.5712852048617059	3.0671094530313372	nuclear_charge= 0.66666666666666663

Table S6. Cartesian coordinate of cluster Ag_{37}-TiO_2 x.

Ti	8.2116172556743159	5.6861522382841798	11.580963256000000
Ti	11.200229198674318	5.6872265392841790	11.525903599999999
Ti	14.175905373674315	5.6866591782841809	11.498403496000000
Ti	17.146494093674317	5.6860720702841796	11.480571418000000
Ti	20.117772881674320	5.6854003742841783	11.487780734999999
Ti	23.095258105674318	5.6855038252841794	11.509230336000000
Ti	26.087567028674314	5.6860311022841792	11.571841807000000
Ti	8.2398455956743177	5.6875189142841798	4.7132988539999996
Ti	11.209846014674316	5.6875189142841798	4.7132988539999996
Ti	14.179845968674318	5.6875189142841798	4.7132988539999996
Ti	17.149846386674316	5.6875189142841798	4.7132988539999996
Ti	20.119846340674314	5.6875189142841798	4.7132988539999996
Ti	23.089847223674319	5.6875189142841798	4.7132988539999996
Ti	26.059845318674313	5.6875189142841798	4.7132988539999996
Ti	8.2435776596743153	2.4004977052841792	7.8087472629999999
Ti	8.2430785796743180	8.9742673482841795	7.8092725439999997
Ti	11.206365465674317	2.3912028602841797	7.8139317589999999
Ti	11.206386841674316	8.9826212582841798	7.8136682310000003
Ti	14.176678622674316	2.3939693712841805	7.8089078640000000
Ti	14.176916545674317	8.9799209152841790	7.8091039569999996
Ti	17.148924436674317	2.3933224322841795	7.8186309029999999
Ti	17.149201393674318	8.9786134802841779	7.8200692170000004
Ti	20.121173502674317	2.3936333752841801	7.8065316730000003
Ti	20.120989484674318	8.9795849202841787	7.8077055719999997
Ti	23.092377010674319	2.3908911802841804	7.8110320609999997
Ti	23.091982951674318	8.9825652592841791	7.8116283270000002
Ti	26.056031120674319	2.4006668822841792	7.8073444409999997
Ti	26.055992086674316	8.9739466792841789	7.8082299659999999
Ti	9.7225611436743158	2.3878913882841797	11.132256293999999
Ti	9.7225425556743161	8.9866313942841813	11.132053988999999
Ti	12.692625689674315	2.3908281072841788	11.129570435000000
Ti	12.693081552674318	8.9828116562841807	11.129475493999999
Ti	15.662512722674318	2.3888209762841797	11.129391201000001
Ti	15.662875183674316	8.9836092032841783	11.130741671999999
Ti	18.634407693674319	2.3883482242841794	11.127390338000000
Ti	18.634376094674316	8.9833427642841812	11.129551802000000
Ti	21.604344449674315	2.3883149202841789	11.125412545000000
Ti	21.603905779674314	8.9822870312841800	11.127110839000000
Ti	24.575044230674315	2.3866607312841790	11.128581094999999
Ti	24.574200348674317	8.9862340942841783	11.129706192000000
Ti	9.7065924426743173	5.6872604332841803	8.1496250349999997
Ti	12.675222946674317	5.6871510882841783	8.0776534309999999
Ti	15.665770218674318	5.6862589312841791	8.0607272870000006
Ti	18.630896475674319	5.6858781362841810	8.0589109830000005
Ti	21.621920986674318	5.6869415322841803	8.0722533209999998
Ti	24.591815920674314	5.6867526092841807	8.1425452640000007
Ti	9.7248458056743168	2.3909869682841798	4.3119031970000004
Ti	9.7248458056743168	8.9840518922841781	4.3119031970000004
Ti	12.694845991674317	2.3909869682841798	4.3119031970000004
Ti	12.694845991674317	8.9840518922841781	4.3119031970000004
Ti	15.664846409674315	2.3909869682841798	4.3119031970000004
Ti	15.664846409674315	8.9840518922841781	4.3119031970000004
Ti	18.634846363674313	2.3909869682841798	4.3119031970000004
Ti	18.634846363674313	8.9840518922841781	4.3119031970000004
Ti	21.604846317674316	2.3909869682841798	4.3119031970000004
Ti	21.604846317674316	8.9840518922841781	4.3119031970000004
Ti	24.574847200674320	2.3909869682841798	4.3119031970000004
Ti	24.574847200674320	8.9840518922841781	4.3119031970000004
O	8.2425528956743150	3.6320255392841787	11.532368471000000
O	8.2351666046743155	10.219983183284178	11.487503009999999
O	11.209931517674317	3.6391342022841791	11.515522184000000
O	11.212031464674315	10.217622372284179	11.503260579000001
O	14.178101511674317	3.6388677642841785	11.510902011000001
O	14.178333857674318	10.217360649284181	11.497350270000000
O	17.148397474674315	3.6384286122841800	11.496051263000000
O	17.148744135674317	10.216788868284180	11.494222536000001

O	20.119051715674317	3.6379953542841790	11.503949124000000
O	20.119013610674315	10.216573123284181	11.497586291999999
O	23.086594374674320	3.6382750562841792	11.505280962000000
O	23.085023712674314	10.216483524284179	11.503778762000000
O	26.054525516674317	3.6321189692841784	11.526642721000000
O	26.061689219674314	10.219424369284180	11.487939561999999
O	8.3186392446743156	4.3439423162841813	8.0162362670000000
O	8.2379049226743177	10.983442854284181	7.9933634360000001
O	11.201772907674318	4.3787004652841794	8.0583404489999992
O	11.212123938674317	10.983036123284180	7.9915870599999996
O	14.171380660674316	4.3860782192841796	8.0188147619999999
O	14.179185175674316	10.980334011284178	7.9890990689999999
O	17.147988545674316	4.3745335312841789	8.0826418350000004
O	17.148935588674316	10.979962648284179	7.9954006770000001
O	20.125489568674318	4.3856078262841791	8.0154314860000007
O	20.118983870674313	10.980400032284180	7.9885533799999999
O	23.096087117674315	4.3780832942841812	8.0538453850000007
O	23.086475413674314	10.983053807284179	7.9908834310000003
O	25.980804802674314	4.3436228252841786	8.0135007199999997
O	26.060986603674316	10.983448749284179	7.9929082500000002
O	8.2398455956743177	3.6882561612841798	4.5247137860000004
O	8.2398455956743177	10.281320642284179	4.5247137860000004
O	11.209846014674316	3.6882561612841798	4.5247137860000004
O	11.209846014674316	10.281320642284179	4.5247137860000004
O	14.179845968674318	3.6882561612841798	4.5247137860000004
O	14.179845968674318	10.281320642284179	4.5247137860000004
O	17.149846386674316	3.6882561612841798	4.5247137860000004
O	17.149846386674316	10.281320642284179	4.5247137860000004
O	20.119846340674314	3.6882561612841798	4.5247137860000004
O	20.119846340674314	10.281320642284179	4.5247137860000004
O	23.089847223674319	3.6882561612841798	4.5247137860000004
O	23.089847223674319	10.281320642284179	4.5247137860000004
O	26.059845318674313	3.6882561612841798	4.5247137860000004
O	26.059845318674313	10.281320642284179	4.5247137860000004
O	8.2365712546743168	1.1527621762841793	11.488183569000000
O	8.2438493886743167	7.7410128212841798	11.535237113000001
O	11.211577459674317	1.1561974362841791	11.503765453000000
O	11.209916647674316	7.7352944132841799	11.517489329000000
O	14.177631242674316	1.1563254972841790	11.498187882000000
O	14.178074094674315	7.7345823382841807	11.514108714000001
O	17.148390968674317	1.1557568842841803	11.494288195999999
O	17.148503424674317	7.7335602052841814	11.500630620000001
O	20.118892790674316	1.1555014972841793	11.497209190000000
O	20.118817510674319	7.7331752832841794	11.508631408999999
O	23.086109235674314	1.1554686352841799	11.503181609000000
O	23.086841590674318	7.7332065252841780	11.509249857000000
O	26.061332335674315	1.1526073312841802	11.488242131000000
O	26.052566371674317	7.7400089612841789	11.530569026000000
O	8.3187886426743169	7.0306988022841814	8.0155388490000004
O	8.2388116546743149	0.39125819328418032	7.9926642430000001
O	11.201731549674317	6.9953886182841813	8.0582153400000003
O	11.212025887674315	0.39061567528417918	7.9913155460000000
O	14.171663193674316	6.9874597132841814	8.0182930300000006
O	14.179367334674318	0.39314212628418055	7.9885631400000001
O	17.148235761674318	6.9975920422841789	8.0829435170000004
O	17.148981128674315	0.39244066128417998	7.9940803740000002
O	20.125049040674320	6.9873453572841804	8.0151715069999998
O	20.118814722674315	0.39261868028417979	7.9876953600000000
O	23.096120575674320	6.9955230172841780	8.0537211630000005
O	23.086538611674314	0.39012641828417927	7.9904317950000001
O	25.980457212674317	7.0307011602841811	8.0130109300000001
O	26.060064653674317	0.39103419628417946	7.9922711690000003
O	8.2398455956743177	1.0937170762841788	4.5247137860000004
O	8.2398455956743177	7.6867813732841803	4.5247137860000004
O	11.209846014674316	1.0937170762841788	4.5247137860000004
O	11.209846014674316	7.6867813732841803	4.5247137860000004
O	14.179845968674318	1.0937170762841788	4.5247137860000004
O	14.179845968674318	7.6867813732841803	4.5247137860000004

O	17.149846386674316	1.0937170762841788	4.5247137860000004
O	17.149846386674316	7.6867813732841803	4.5247137860000004
O	20.119846340674314	1.0937170762841788	4.5247137860000004
O	20.119846340674314	7.6867813732841803	4.5247137860000004
O	23.089847223674319	1.0937170762841788	4.5247137860000004
O	23.089847223674319	7.6867813732841803	4.5247137860000004
O	26.059845318674313	1.0937170762841788	4.5247137860000004
O	26.059845318674313	7.6867813732841803	4.5247137860000004
O	6.7508418266743178	5.6857195702841814	12.802871836000000
O	27.540842741674318	5.6857195702841814	12.802871836000000
O	9.7382428966743149	5.6862495002841804	12.815872561999999
O	12.700224344674318	5.6854451732841795	12.786887117999999
O	15.669960817674315	5.6830767002841789	12.761814668000000
O	18.626778368674316	5.6814129322841787	12.755000201000000
O	21.595247624674315	5.6810100322841812	12.770365583000000
O	24.555452784674316	5.6808936132841801	12.797999668999999
O	6.7564803556743165	5.6883447562841809	10.194717754999999
O	27.546481270674313	5.6883447562841809	10.194717754999999
O	9.7153093806743165	5.6876963432841805	10.161842386000000
O	12.686084674674316	5.6876827862841814	10.138575593000001
O	15.651230912674315	5.6876232502841795	10.125623667999999
O	18.640681045674313	5.6872925592841810	10.121267022000000
O	21.608366829674317	5.6874408102841798	10.127841919000000
O	24.583596435674316	5.6875760922841785	10.151808792000001
O	9.7248458056743168	2.3909869682841798	2.4691267400000001
O	9.7248458056743168	8.9840518922841781	2.4691267400000001
O	12.694845991674317	2.3909869682841798	2.4691267400000001
O	12.694845991674317	8.9840518922841781	2.4691267400000001
O	15.664846409674315	2.3909869682841798	2.4691267400000001
O	15.664846409674315	8.9840518922841781	2.4691267400000001
O	18.634846363674313	2.3909869682841798	2.4691267400000001
O	18.634846363674313	8.9840518922841781	2.4691267400000001
O	21.604846317674316	2.3909869682841798	2.4691267400000001
O	21.604846317674316	8.9840518922841781	2.4691267400000001
O	24.574847200674320	2.3909869682841798	2.4691267400000001
O	24.574847200674320	8.9840518922841781	2.4691267400000001
O	6.7548456196743167	5.6875189142841798	3.2807227370000001
O	27.544846534674313	5.6875189142841798	3.2807227370000001
O	9.7248458056743168	5.6875189142841798	3.2807227370000001
O	12.694845991674317	5.6875189142841798	3.2807227370000001
O	15.664846409674315	5.6875189142841798	3.2807227370000001
O	18.634846363674313	5.6875189142841798	3.2807227370000001
O	21.604846317674316	5.6875189142841798	3.2807227370000001
O	24.574847200674320	5.6875189142841798	3.2807227370000001
O	6.7543363156743155	2.3989356212841795	9.2348139210000006
O	27.544337231674319	2.3989356212841795	9.2348139210000006
O	6.7541392866743166	8.9726610542841811	9.2360277479999997
O	27.544140201674317	8.9726610542841811	9.2360277479999997
O	9.7317502096743169	2.3561228632841793	9.2426461220000000
O	9.7316907296743160	9.0172842082841811	9.2426807269999998
O	12.697624389674317	2.3797824012841797	9.2372398009999994
O	12.697557008674316	8.9925343062841812	9.2373143340000006
O	15.657047006674315	2.3761785542841789	9.2380782999999997
O	15.657006113674317	8.9951774702841796	9.2392814790000006
O	18.640746102674314	2.3758139702841792	9.2365432700000003
O	18.640852052674319	8.9952381852841796	9.2384021640000000
O	21.600496793674317	2.3783418942841799	9.2338494250000007
O	21.600606460674314	8.9927618392841779	9.2353436389999999
O	24.566861920674313	2.3550999922841793	9.2396949609999997
O	24.566620280674314	9.0174793222841814	9.2407171310000003
O	6.7547229406743163	5.6873883482841805	5.9537143029999999
O	27.544723855674313	5.6873883482841805	5.9537143029999999
O	9.7056228606743176	5.6876798382841791	5.9679670109999998
O	12.576099804674318	5.6873683062841813	6.0511443319999998
O	15.754665938674318	5.6871319302841812	6.0566487000000002
O	18.544141130674319	5.6869907532841779	6.0559322050000004
O	21.722680312674314	5.6873747902841814	6.0490924489999998
O	24.593646809674318	5.6876362182841795	5.9673188379999997

O	6.7552938086743168	2.4009284572841789	6.5628873370000003		
O	27.545294723674317	2.4009284572841789	6.5628873370000003		
O	6.7550022916743160	8.9739331212841797	6.5633895490000000		
O	27.545003207674320	8.9739331212841797	6.5633895490000000		
O	9.7354742576743156	2.3769759542841804	6.5628212340000003		
O	9.7350899566743152	8.9967595972841785	6.5630217630000001		
O	12.691325479674315	2.3939419612841792	6.5722474699999998		
O	12.691529014674316	8.9800134612841802	6.5723690299999999		
O	15.667184743674316	2.3956903162841794	6.5765482149999999		
O	15.667773973674315	8.9770030592841792	6.5773494479999997		
O	18.629714296674315	2.3953373742841801	6.5757718279999997		
O	18.629615782674314	8.9772636032841788	6.5770273579999996		
O	21.607002491674315	2.3931099302841794	6.5701028650000000		
O	21.606703229674316	8.9805634332841784	6.5710806699999997		
O	24.564235477674316	2.3763077952841805	6.5609698810000001		
O	24.564192725674317	8.9972046432841815	6.5616859329999997		
Ag	17.120663682674316	5.7730963882841806	17.435367340999999		
Ag	20.007152776674317	5.7436189692841779	17.417685216999999		
Ag	14.233835828674316	5.7999164952841795	17.446274037999999		
Ag	22.999165224674314	5.7160873772841789	17.392425546999998		
Ag	11.244726780674316	5.8256166172841795	17.447952811000000		
Ag	26.135629268674315	5.7090509232841811	17.460941115000001		
Ag	21.536140567674316	5.6326982952841789	14.966656079000000		
Ag	21.479271470674320	7.9855916362841803	16.583912032000001		
Ag	21.462318550674318	7.2391751912841791	19.322611082000002		
Ag	21.437871995674314	4.3818261072841800	19.440524442000001		
Ag	15.645347902674317	3.4612852802841800	16.796991917000000		
Ag	15.625609614674318	5.6796256722841783	14.970301108999999		
Ag	15.689575498674316	8.0529292682841813	16.600744121999998		
Ag	15.706713366674318	7.3345637352841813	19.367641581000001		
Ag	15.674989722674315	4.4170906082841803	19.497696761000000		
Ag	21.435020640674317	3.4219540552841803	16.762097939000000		
Ag	24.491834490674314	5.6153470732841804	14.994467625000000		
Ag	24.454333787674315	7.9191281822841795	16.629173228999999		
Ag	24.354413697674318	7.1840884282841806	19.305219993000001		
Ag	24.328625109674313	4.3883550312841813	19.421834345000001		
Ag	12.738311025674317	3.5050990982841803	16.825243564000001		
Ag	12.708292340674316	5.7014603712841812	14.996586498999999		
Ag	12.782632024674317	8.0595949482841789	16.623813935000001		
Ag	12.843868768674316	7.3310210452841780	19.369386014000000		
Ag	12.812817535674316	4.4770687582841795	19.498573413999999		
Ag	24.430056380674316	3.4526277952841795	16.800880068000001		
Ag	18.612164711674318	5.6520221652841798	14.959664151000000		
Ag	18.576588765674316	8.0265294512841798	16.588646667999999		
Ag	18.598288056674313	7.2997369132841783	19.354685220000000		
Ag	18.570033809674314	4.3817356242841790	19.477966747000000		
Ag	18.529170589674315	3.4318208292841792	16.776631920000000		
Ag	8.1044397106743169	5.8438717852841791	17.544084719000001		
Ag	9.7528760706743149	3.5882677202841791	16.893464548000001		
Ag	9.7676179826743166	5.7231208842841781	15.032969367000000		
Ag	9.7855876506743158	8.0231123172841805	16.689103278000001		
Ag	9.9475739616743155	7.3236362172841787	19.375598891999999		
Ag	9.9260164006743175	4.5315937612841815	19.508527149999999		
H	7.4819644642565528	2.9994857293307202	11.326682334331748	nuclear_charge=	0.66666666666666663
H	8.2362682063174120	11.229980712873434	11.515997512933367	nuclear_charge=	0.66666666666666663
H	7.4742365404264390	9.5809378980127349	11.304432748362387	nuclear_charge=	0.66666666666666663
H	11.210224567510220	11.227770985509835	11.525725536621243	nuclear_charge=	0.66666666666666663
H	14.178019459707070	11.227465726834541	11.521759150779294	nuclear_charge=	0.66666666666666663
H	17.148769467238633	11.226887549261935	11.518896693438782	nuclear_charge=	0.66666666666666663
H	20.119375700931833	11.226684427651715	11.521735446841664	nuclear_charge=	0.66666666666666663
H	23.087079465298977	11.226641303164840	11.525805919057520	nuclear_charge=	0.66666666666666663
H	26.815686164528735	2.9993424414219287	11.323821385967307	nuclear_charge=	0.66666666666666663
H	26.061038768279907	11.229430767149999	11.516131993308694	nuclear_charge=	0.66666666666666663
H	26.822767871519069	9.5806238207968093	11.304632974670458	nuclear_charge=	0.66666666666666663
H	7.5534582336601339	5.0012604493119213	8.0740208010574790	nuclear_charge=	0.66666666666666663
H	7.4796283501122325	11.646813919491940	8.0699377747083236	nuclear_charge=	0.66666666666666663
H	8.9962437680314373	11.646691978739646	8.0703761267171306	nuclear_charge=	0.66666666666666663
H	10.451704607473580	11.643829198706296	8.0691957722068430	nuclear_charge=	0.66666666666666663

H	11.969973108966432	11.646865488801321	8.0684204418379757	nuclear_charge= 0.66666666666666663
H	13.420916097520710	11.643562370106388	8.0669727434354677	nuclear_charge= 0.66666666666666663
H	14.937373823430182	11.643759302406881	8.0660732043500651	nuclear_charge= 0.66666666666666663
H	16.390623540034920	11.643611983220268	8.0691608093697909	nuclear_charge= 0.66666666666666663
H	17.907353935303640	11.643510897601097	8.0689771156505383	nuclear_charge= 0.66666666666666663
H	19.360732203994424	11.643741591422067	8.0656283168970440	nuclear_charge= 0.66666666666666663
H	20.877397792635136	11.643468710695924	8.0663763244656650	nuclear_charge= 0.66666666666666663
H	22.328501587235891	11.646737509349126	8.0677455286011917	nuclear_charge= 0.66666666666666663
H	23.846940684628436	11.643785330491408	8.0685660282334197	nuclear_charge= 0.66666666666666663
H	26.745697515623029	5.0011569878413553	8.0726287399921262	nuclear_charge= 0.66666666666666663
H	26.819146702433237	11.646924593866473	8.0697277026515160	nuclear_charge= 0.66666666666666663
H	25.302531903707717	11.646572476544769	8.0698598610461598	nuclear_charge= 0.66666666666666663
H	7.4833010548965859	3.0273521963581107	4.4162957664441551	nuclear_charge= 0.66666666666666663
H	8.2398459076291388	11.287255328178833	4.6196009174453465	nuclear_charge= 0.66666666666666663
H	7.4833009812900393	9.6204167633467996	4.4162957558958320	nuclear_charge= 0.66666666666666663
H	11.209846092663021	11.287255328178876	4.6196009174453510	nuclear_charge= 0.66666666666666663
H	14.179846046663023	11.287255328178876	4.6196009174453510	nuclear_charge= 0.66666666666666663
H	17.149846230696905	11.287255328178869	4.6196009174453501	nuclear_charge= 0.66666666666666663
H	20.119846184696904	11.287255328178869	4.6196009174453501	nuclear_charge= 0.66666666666666663
H	23.089846600267826	11.287255328178688	4.6196009174453332	nuclear_charge= 0.66666666666666663
H	26.816390552896436	3.0273529693510444	4.4162958932498144	nuclear_charge= 0.66666666666666663
H	26.059845630629134	11.287255328178833	4.6196009174453465	nuclear_charge= 0.66666666666666663
H	26.816390626502873	9.6204175363397830	4.4162958827015286	nuclear_charge= 0.66666666666666663
H	8.2369841529994474	0.14275494521375975	11.516350618906566	nuclear_charge= 0.66666666666666663
H	7.4747846682803285	1.7904783511019335	11.304043847141832	nuclear_charge= 0.66666666666666663
H	7.4822056638441836	8.3719931126003040	11.328667830681688	nuclear_charge= 0.66666666666666663
H	11.209992906747654	0.14604298923508807	11.525983515448576	nuclear_charge= 0.66666666666666663
H	14.177660907417920	0.14621068498440870	11.522192570971161	nuclear_charge= 0.66666666666666663
H	17.148589304637664	0.14565768873099927	11.518940494182187	nuclear_charge= 0.66666666666666663
H	20.119314194338102	0.14539486083652520	11.521551856726514	nuclear_charge= 0.66666666666666663
H	23.087633552153093	0.14531639130650476	11.525503805266089	nuclear_charge= 0.66666666666666663
H	26.060856796513612	0.14259683785402011	11.516290963836139	nuclear_charge= 0.66666666666666663
H	26.823043635663893	1.7904046123436466	11.304071873517040	nuclear_charge= 0.66666666666666663
H	26.814473288064477	8.3714529957190287	11.326401344587204	nuclear_charge= 0.66666666666666663
H	7.5533994696198299	6.3736540655555167	8.0736747906207249	nuclear_charge= 0.66666666666666663
H	7.4798375106690926	-0.27127050193440816	8.0696196634945565	nuclear_charge= 0.66666666666666663
H	8.9965578957140693	-0.27262782116956252	8.0700215568290492	nuclear_charge= 0.66666666666666663
H	10.451740513717649	-0.27031627046562079	8.0690540386208180	nuclear_charge= 0.66666666666666663
H	11.970054175032221	-0.27299152790122783	8.0683009695922632	nuclear_charge= 0.66666666666666663
H	13.420864815241870	-0.26978536076098614	8.0667247350106503	nuclear_charge= 0.66666666666666663
H	14.937371632740611	-0.27046307204277120	8.0658016334244103	nuclear_charge= 0.66666666666666663
H	16.390554087514186	-0.27100094767676275	8.0685237399664054	nuclear_charge= 0.66666666666666663
H	17.907412639077872	-0.27101703561016066	8.0683345890900178	nuclear_charge= 0.66666666666666663
H	19.360720460153381	-0.27085256625625220	8.0652011929864447	nuclear_charge= 0.66666666666666663
H	20.877414067810655	-0.27018509834586091	8.0659663290272228	nuclear_charge= 0.66666666666666663
H	22.328430867075149	-0.27337629380556194	8.0675353528188438	nuclear_charge= 0.66666666666666663
H	23.846899500352066	-0.27069899820275367	8.0683371728249611	nuclear_charge= 0.66666666666666663
H	26.745680109084205	6.3735740126752809	8.0723909829256453	nuclear_charge= 0.66666666666666663
H	26.818982601342697	-0.27153341898729089	8.0694454127623967	nuclear_charge= 0.66666666666666663
H	25.302240559563806	-0.27277412131397227	8.0695325427458577	nuclear_charge= 0.66666666666666663
H	8.2398459076290962	8.7782389138180150E-002	4.6196009041793618	nuclear_charge= 0.66666666666666663
H	7.4833012048543175	1.7546212163939110	4.4162957879341267	nuclear_charge= 0.66666666666666663
H	7.4833013243713644	8.3476856530160894	4.4162958050617389	nuclear_charge= 0.66666666666666663
H	11.209846092663010	8.7782389138135741E-002	4.6196009041793662	nuclear_charge= 0.66666666666666663
H	14.179846046663013	8.7782389138135741E-002	4.6196009041793662	nuclear_charge= 0.66666666666666663
H	17.149846230696927	8.7782389138144623E-002	4.6196009041793653	nuclear_charge= 0.66666666666666663
H	20.119846184696925	8.7782389138144623E-002	4.6196009041793653	nuclear_charge= 0.66666666666666663
H	23.089846600267911	8.7782389138324035E-002	4.6196009041793484	nuclear_charge= 0.66666666666666663
H	26.059845630629091	8.7782389138180150E-002	4.6196009041793618	nuclear_charge= 0.66666666666666663
H	26.816390402938914	1.7546204434010786	4.4162959147397105	nuclear_charge= 0.66666666666666663
H	26.816390283422038	8.3476848800233370	4.4162959318672623	nuclear_charge= 0.66666666666666663
H	5.9798657260377475	5.6858850493587099	12.149799110444947	nuclear_charge= 0.66666666666666663
H	28.315853123653866	5.6859488600042241	12.154591792153102	nuclear_charge= 0.66666666666666663
H	6.0217208911783331	5.6871797183652255	10.888284862077766	nuclear_charge= 0.66666666666666663
H	6.7554383345410720	5.6880376623530715	9.1843183389840970	nuclear_charge= 0.66666666666666663
H	28.278049255244284	5.6872422239821248	10.891650483729792	nuclear_charge= 0.66666666666666663
H	27.545439553589773	5.6880376623529756	9.1843183386705789	nuclear_charge= 0.66666666666666663
H	9.0120814485719407	2.3909869447653485	1.7529737317367968	nuclear_charge= 0.66666666666666663

H	10.437610349408388	2.3909869447653556	1.7529739174854462	nuclear_charge=	0.66666666666666663
H	9.0120814485719443	8.9840517977288812	1.7529737317367999	nuclear_charge=	0.66666666666666663
H	10.437610349408384	8.9840517977289061	1.7529739174854493	nuclear_charge=	0.66666666666666663
H	11.982081578630641	2.3909869447653502	1.7529737874133993	nuclear_charge=	0.66666666666666663
H	13.407610479467120	2.3909869447653538	1.7529738618088531	nuclear_charge=	0.66666666666666663
H	11.982081578630645	8.9840517977288883	1.7529737874134024	nuclear_charge=	0.66666666666666663
H	13.407610479467117	8.9840517977288989	1.7529738618088562	nuclear_charge=	0.66666666666666663
H	14.952081884748083	2.3909869447653538	1.7529738987665917	nuclear_charge=	0.66666666666666663
H	16.377610785343418	2.3909869447653502	1.7529737502156699	nuclear_charge=	0.66666666666666663
H	14.952081884748086	8.9840517977289025	1.7529738987665946	nuclear_charge=	0.66666666666666663
H	16.377610785343414	8.9840517977288847	1.7529737502156726	nuclear_charge=	0.66666666666666663
H	17.922081838506955	2.3909869447653538	1.7529738990065771	nuclear_charge=	0.66666666666666663
H	19.347610739343416	2.3909869447653502	1.7529737502156708	nuclear_charge=	0.66666666666666663
H	17.922081838506958	8.9840517977289025	1.7529738990065800	nuclear_charge=	0.66666666666666663
H	19.347610739343416	8.9840517977288847	1.7529737502156735	nuclear_charge=	0.66666666666666663
H	20.892081792506957	2.3909869447653538	1.7529738990065771	nuclear_charge=	0.66666666666666663
H	22.317610693343418	2.3909869447653502	1.7529737502156708	nuclear_charge=	0.66666666666666663
H	20.892081792506964	8.9840517977289025	1.7529738990065800	nuclear_charge=	0.66666666666666663
H	22.317610693343418	8.9840517977288847	1.7529737502156735	nuclear_charge=	0.66666666666666663
H	23.862082451500882	2.3909869447653627	1.7529741219528967	nuclear_charge=	0.66666666666666663
H	25.287611352336988	2.3909869447653431	1.7529735272692348	nuclear_charge=	0.66666666666666663
H	23.862082451500882	8.9840517977289309	1.7529741219528996	nuclear_charge=	0.66666666666666663
H	25.287611352336988	8.9840517977288528	1.7529735272692377	nuclear_charge=	0.66666666666666663
H	6.0276643759123161	5.6875191067299689	3.9822322513479596	nuclear_charge=	0.66666666666666663
H	6.7548459623725705	5.6875191318615848	2.2703227370000816	nuclear_charge=	0.66666666666666663
H	28.272027339390661	5.6875191067300932	3.9822327064601968	nuclear_charge=	0.66666666666666663
H	27.544847220624455	5.6875191318615848	2.2703227370002566	nuclear_charge=	0.66666666666666663
H	9.7248460199299309	5.6875191318615848	2.2703227370000461	nuclear_charge=	0.66666666666666663
H	12.694846077487288	5.6875191318615848	2.2703227370000274	nuclear_charge=	0.66666666666666663
H	15.664846238048373	5.6875191318615848	2.2703227370000381	nuclear_charge=	0.66666666666666663
H	18.634847221250403	5.6875191318615848	2.2703227370003871	nuclear_charge=	0.66666666666666663
H	21.604847175250406	5.6875191318615848	2.2703227370003871	nuclear_charge=	0.66666666666666663
H	24.574847543372577	5.6875191318615848	2.2703227370000816	nuclear_charge=	0.66666666666666663
H	6.0251276177673567	2.3997839879119827	8.5354126662321068	nuclear_charge=	0.66666666666666663
H	6.7540908549126826	2.3962902676405076	10.245210428245109	nuclear_charge=	0.66666666666666663
H	28.274108678045600	2.3997009193142187	8.5359997751929075	nuclear_charge=	0.66666666666666663
H	27.544092100962757	2.3962902676402980	10.245210428325235	nuclear_charge=	0.66666666666666663
H	6.0250479251611111	8.9732905380067791	8.5365039454621119	nuclear_charge=	0.66666666666666663
H	6.7538105793757772	8.9742638626003988	10.246426423254977	nuclear_charge=	0.66666666666666663
H	28.273672495366370	8.9734477011413674	8.5369639643241939	nuclear_charge=	0.66666666666666663
H	27.543811824952932	8.9742638626005693	10.246426423362468	nuclear_charge=	0.66666666666666663
H	5.9792874897148920	5.6874567378855012	5.3059427254217466	nuclear_charge=	0.66666666666666663
H	6.7545526989274016	5.6875413451639929	6.9641142770744757	nuclear_charge=	0.66666666666666663
H	28.320211563613974	5.6874567312805162	5.3060052864205991	nuclear_charge=	0.66666666666666663
H	27.544553901202839	5.6875413451640000	6.9641142771228379	nuclear_charge=	0.66666666666666663
H	5.9799553323625148	2.4007924040001232	7.2107749728134944	nuclear_charge=	0.66666666666666663
H	6.7550929097427748	2.3964660394231565	5.5524972111246269	nuclear_charge=	0.66666666666666663
H	28.320063792521033	2.4007040377911526	7.2114557602170732	nuclear_charge=	0.66666666666666663
H	27.545094103039652	2.3964660394229114	5.5524972110693316	nuclear_charge=	0.66666666666666663
H	5.9798160639406923	8.9739397704477497	7.2114593541042833	nuclear_charge=	0.66666666666666663
H	6.7549322604443383	8.9784739899815591	5.5529997551050396	nuclear_charge=	0.66666666666666663
H	28.319722045827611	8.9741067164902653	7.2120179878423958	nuclear_charge=	0.66666666666666663
H	27.544933454230289	8.9784739899816444	5.5529997550858248	nuclear_charge=	0.66666666666666663
